# Supplementary material for: Correlational networking guides the discovery of unclustered lanthipeptide protease-encoding genes
Source: Nat Commun. 2022 Mar 28;13:1647. doi: 10.1038/s41467-022-29325-1 (PMC8960859; doi:10.1038/s41467-022-29325-1)
Supplement: Supplementary file 1 — Supplementary Information_0219_2022 [file 41467_2022_29325_MOESM1_ESM.pdf]

Supplementary Information

Correlational networking guides the discovery of unclustered lanthipeptide protease-encoding genes

Dan Xue[1,9], Ethan A. Older[1,9], Zheng Zhong[2,3,9], Zhuo Shang[1], Nanzhu Chen[2,3], Nolan Dittenhauser[1], Lukuan Hou[1], Peiyan Cai[2,3], Michael D. Walla[4], Shi-Hui Dong[5], Xiaoyu Tang[6], Hexin Chen[7], Prakash Nagarkatti[8], Mitzi Nagarkatti[8], Yong-Xin Li [2,3*] and Jie Li[1*]

[1]Department of Chemistry and Biochemistry, University of South Carolina, Columbia, South Carolina, USA. [2]Department of Chemistry and The Swire Institute of Marine Science, The University of Hong Kong, Pokfulam Road, Hong Kong, China. [3]Southern Marine Science and Engineering Guangdong Laboratory (Guangzhou), Guangzhou, China. [4]The Mass Spectrometry Center, Department of Chemistry and Biochemistry, University of South Carolina, Columbia, South Carolina, USA. [5]State Key Laboratory of Applied Organic Chemistry, College of Chemistry and Chemical Engineering, Lanzhou University, Lanzhou, China. [6]Institute of Chemical Biology, Shenzhen Bay Laboratory, Shenzhen, China. [7]Department of Biological Sciences, University of South Carolina, Columbia, South Carolina, USA. [8]Department of Pathology, Microbiology and Immunology, School of Medicine, University of South Carolina, Columbia, South Carolina, USA.

[9]These authors contributed equally to this work.

[*]To whom correspondence may be addressed.
Email: yxpli@hku.hk; li439@mailbox.sc.edu

Table of Contents

Supplementary Figures 4

Supplementary Figure 1: Lanthipeptide BGCs without proteases. 4

Supplementary Figure 2: Precursor sequences of selected class III lanthipeptide precursor groups. 5

Supplementary Figure 3: Prioritized correlation network of lanthipeptide precursor and protease groups. 6

Supplementary Figure 4: Identification of previously characterized lanthipeptide proteases to validate the correlational network. 7

Supplementary Figure 5: Class III lanthipeptide BGCs containing FlaA-like precursors 8

Supplementary Figure 6: Bacinapeptin A heterologous production and structure elucidation by MS/MS analysis 9

Supplementary Figure 7: Bacinapeptin B heterologous production and structure elucidation by MS/MS analysis 11

Supplementary Figure 8: Heterologous production of paenithopepins A-E 12

Supplementary Figure 9: High-resolution mass spectra of paenithopeptin A. 13

Supplementary Figure 10: MSMS spectrum of paenithopeptin A. 14

Supplementary Figure 11: Chemical reduction for the determination of disulfide bond presence. 15

Supplementary Figure 12: MS/MS spectrum of paenithopeptin A after DTT treatment 16

Supplementary Figure 13: Extracted ion chromatograms (EICs) of paenithopeptin A mutations. 17

Supplementary Figure 14: ^{1}H-NMR (500 MHz, DMSO-d_6) spectrum of paenithopeptin A 18

Supplementary Figure 15: HSQC NMR (500 MHz, DMSO-d_6) spectrum of paenithopeptin A 19

Supplementary Figure 16: HMBC NMR (500 MHz, DMSO-d_6) spectrum of paenithopeptin A 20

Supplementary Figure 17: ^{1}H-^{1}H COSY NMR (500 MHz, DMSO-d_6) spectrum of paenithopeptin A 21

Supplementary Figure 18: ^{1}H-^{1}H TOCSY NMR (500 MHz, DMSO-d_6) spectrum of paenithopeptin A. 22

Supplementary Figure 19: HSQC-TOCSY NMR (500 MHz, DMSO-d_6) spectrum of paenithopeptin 23

Supplementary Figure 20: ROESY NMR (500 MHz, DMSO-d_6) spectrum of paenithopeptin A 24

Supplementary Figure 21: MS/MS spectrum of paenithopeptin B 25

Supplementary Figure 22: MS/MS spectrum of paenithopeptin C 26

Supplementary Figure 23: MS/MS spectrum of paenithopeptin D 27

Supplementary Figure 24: MS/MS spectrum of paenithopeptin E 28

Supplementary Figure 25: Homology modeling of Bcn-gP1 and Bcn-gP2 29

Supplementary Figure 26: Homology modeling of PttP1 and PttP2 30

Supplementary Figure 27: Pull-down assay and in vivo proteolytic activity of PttP1/PttP2 31

Supplementary Figure 28: Different efficiencies of Prot_819/Prot_176 proteases against PttKC-modified PttA1 32

Supplementary Figure 29: Different efficiencies of Prot_819/Prot_176 proteases against BcnKC-modified BcnA1. 33

Supplementary Figure 30: Phylogenetic tree of Prot_176 in *Paenibacillus*. 34

Supplementary Figure 31: In vitro activity of PttP1/PttP2 mutations. 35

Supplementary Figure 32: PttP1/PttP2 activity is metal dependent 36

Supplementary Figure 33: PttP1/PttP2 are responsible for the processing of paenithopeptin A2........................37

Supplementary Figure 34: PttP1/PttP2 are responsible for the processing of paenithopeptin A3........................38

Supplementary Figure 35: PttP1/PttP2 are responsible for the processing of paenithopeptin A5........................39

Supplementary Figure 36: PttP1/PttP2 are responsible for the processing of paenithopeptin A7........................40

Supplementary Figure 37: In vitro production and LC-MS analysis of paenithopeptin A2 and analogs by PttP1/PttP2 41

Supplementary Figure 38: In vitro production and LC-MS analysis of paenithopeptin A3 and analogs by PttP1/PttP2 43

Supplementary Figure 39: In vitro production and LC-MS analysis of paenithopeptin A5 and analogs by PttP1/PttP2 46

Supplementary Figure 40: In vitro production and LC-MS analysis of paenithopeptin A7 and analogs by PttP1/PttP2 48

Supplementary Tables *50*

Supplementary Table 1 50

Supplementary Table 2 52

Supplementary Table 3 54

Supplementary Table 4 55

Supplementary Table 5 57

Supplementary Table 6 60

Supplementary Note *61*

Structure elucidation of bacinapeptins A and B...61

Paenithopeptin A structure elucidation..61

Networking analysis via Spearman's rank-order correlation...62

Supplementary References.. *62*

Supplementary Figures

Supplementary Figure 1: Lanthipeptide BGCs without proteases.

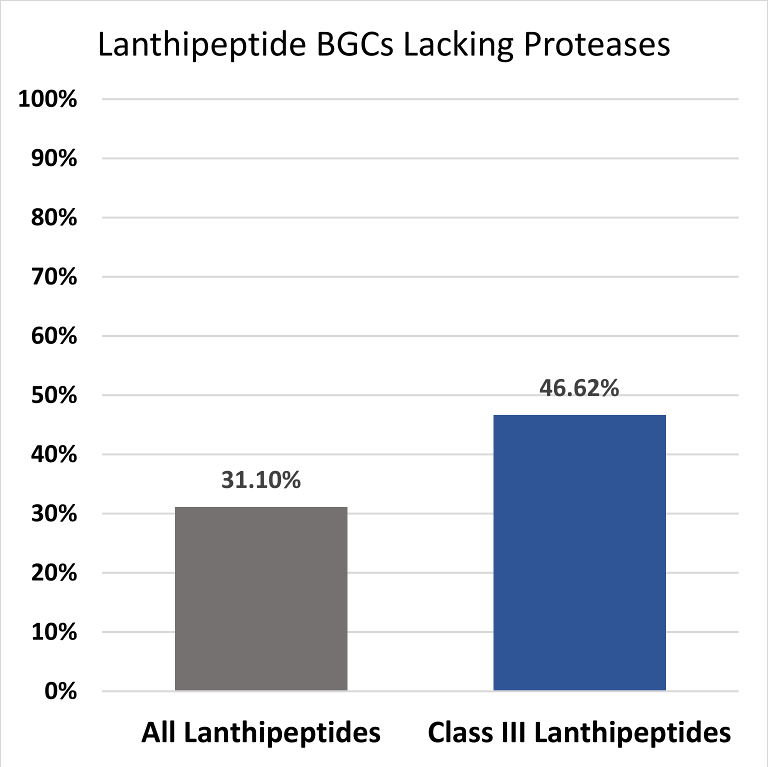

A bar graph depicting the percentage of lanthipeptide BGCs which were determined to lack a putative protease encoding gene. Overall, 31.1% of lanthipeptide BGCs did not contain putative proteases while 46.6% of class III lanthipeptides BGCs did not contain putative proteases. Genomic regions 10 kb up- and downstream of LanC-like proteins were searched for putative lanthipeptide proteases. The number of proteases retrieved this way was likely inflated due to the wide genomic range searched, which possibly included non-lanthipeptide proteases. As a result, this may lead to an underestimation of the percentage of lanthipeptide BGCs that do not harbor any proteases.

Supplementary Figure 2: Precursor sequences of selected class III lanthipeptide precursor groups.

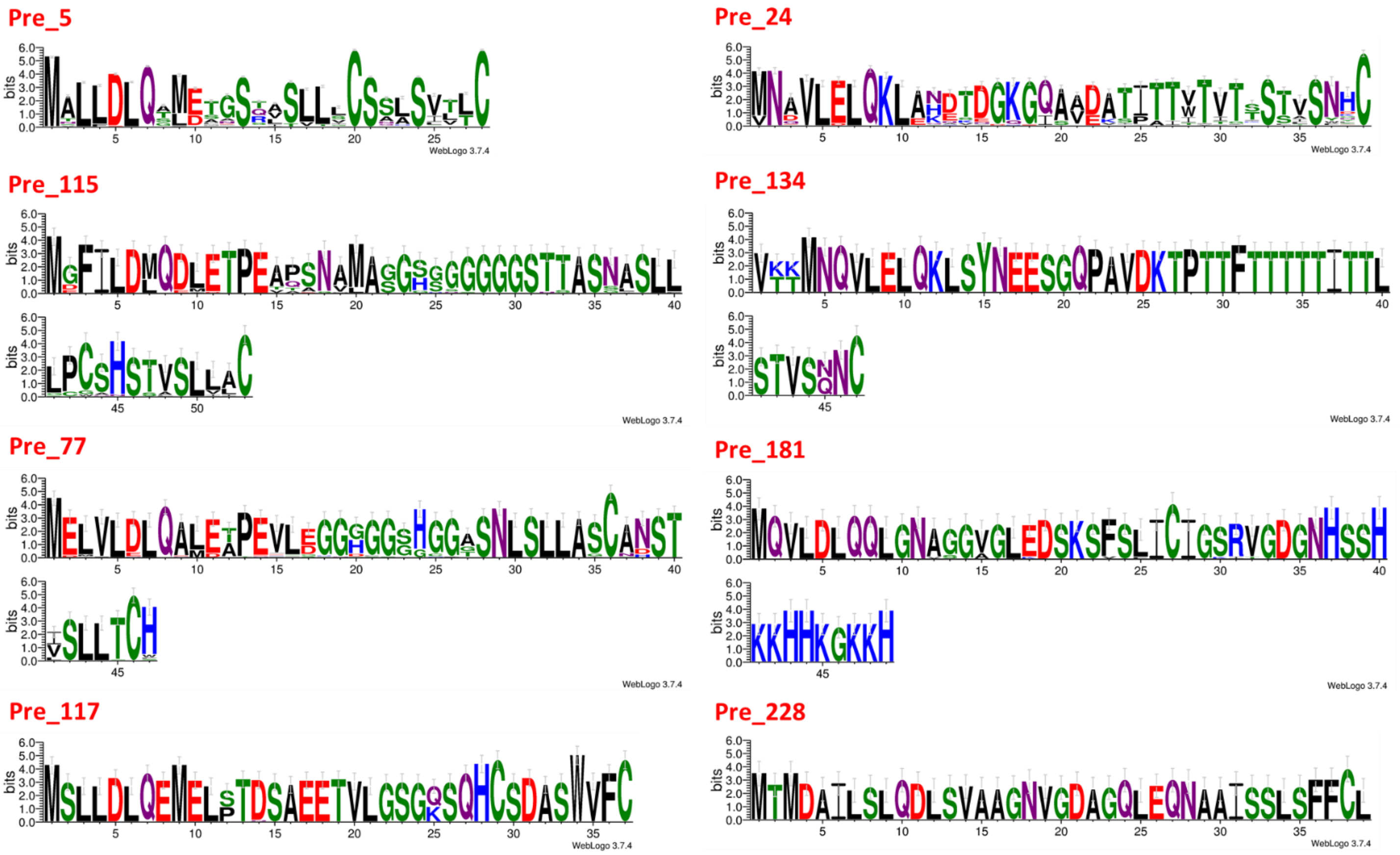

Sequence logos representing the conserved motifs in prioritized class III lanthipeptide precursor groups. The 8 precursor groups depicted were all identified through the prioritization of significant correlations as described by Spearman's ρ, adjusted p-value, and number of genomes containing the correlation (ρ>0.3, pAdj<1E-5, one-sided *t*-test, adjusted by false-discovery rate, I>=10). These precursor groups correlate with 87 groups of proteases. Precursor sequences in each group were aligned and gaps were trimmed by trimAl before calculating logos using WebLogo v3.7.4. Error bars indicate sample correction, and the total height of the error bar is twice this correction.

Supplementary Figure 3: **Prioritized correlation network of lanthipeptide precursor and protease groups.**

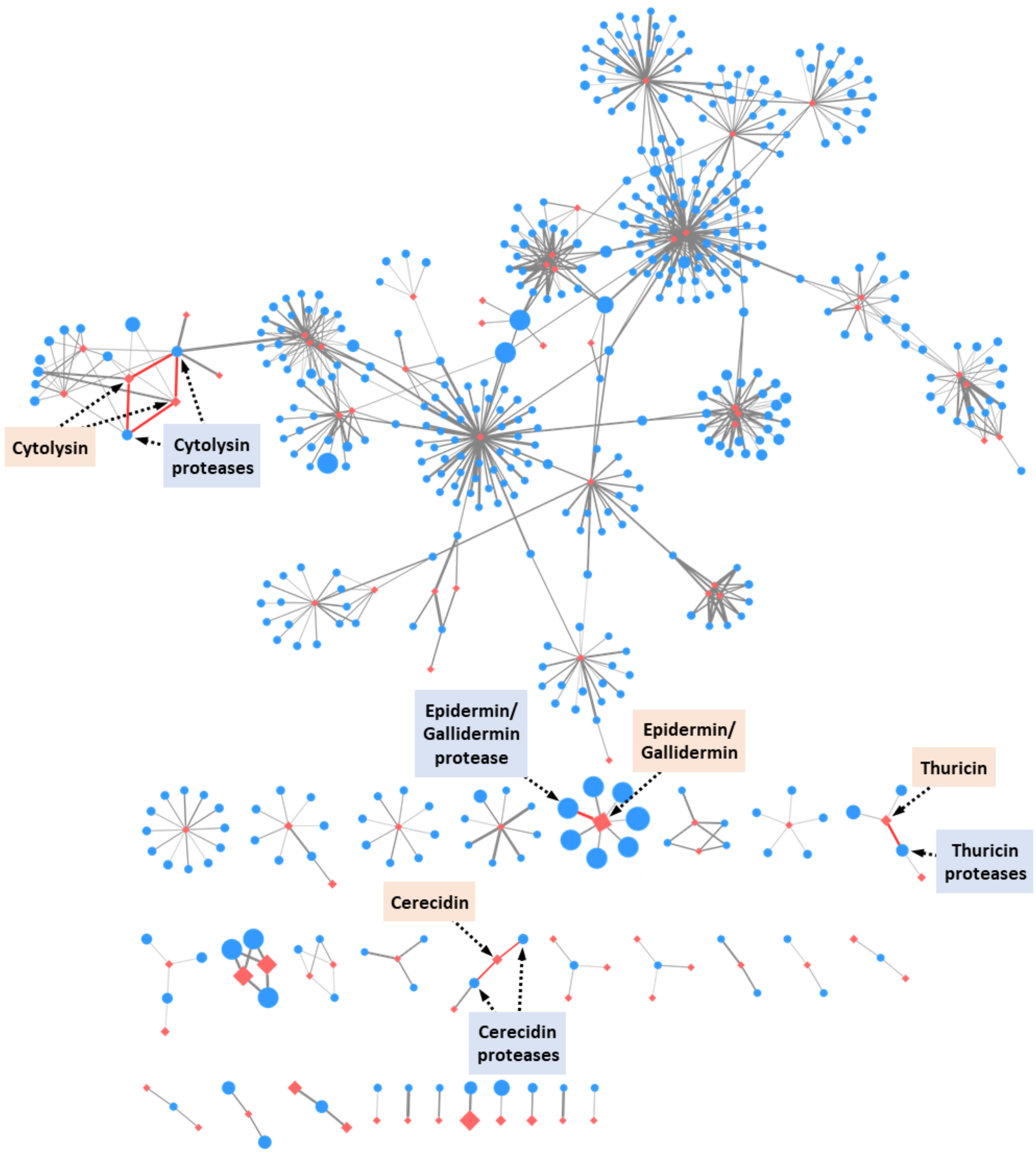

Correlational networking was performed between lanthipeptide precursor groups and protease groups. Network analysis was visualized using Cytoscape. Clusters were filtered for display using a set of thresholds: Spearman's correlation coefficient (ρ>0.5), false-discovery-rate adjusted p-value (pAdj<1E-5, one-sided *t*-test, adjusted by false-discovery rate), and number of genomes containing the correlation (I >= 10). Red diamonds represent precursor groups and blue circles represent protease groups. Shape sizes are scaled proportionally to the number of elements (proteases or precursors) contained in that group at the genus level. Groups containing more than 1000 elements were scaled individually (Supplementary Data 1: FigS3_cytoscape). Edges between circles and diamonds indicate significant correlations as described above with increasing thickness of the edge representing increasing strength of Spearman's correlation coefficient (ρ). Red edges connecting named precursor and protease groups represent literature-reported associations between the two groups.

Supplementary Figure 4: Identification of previously characterized lanthipeptide proteases to validate the correlational network.

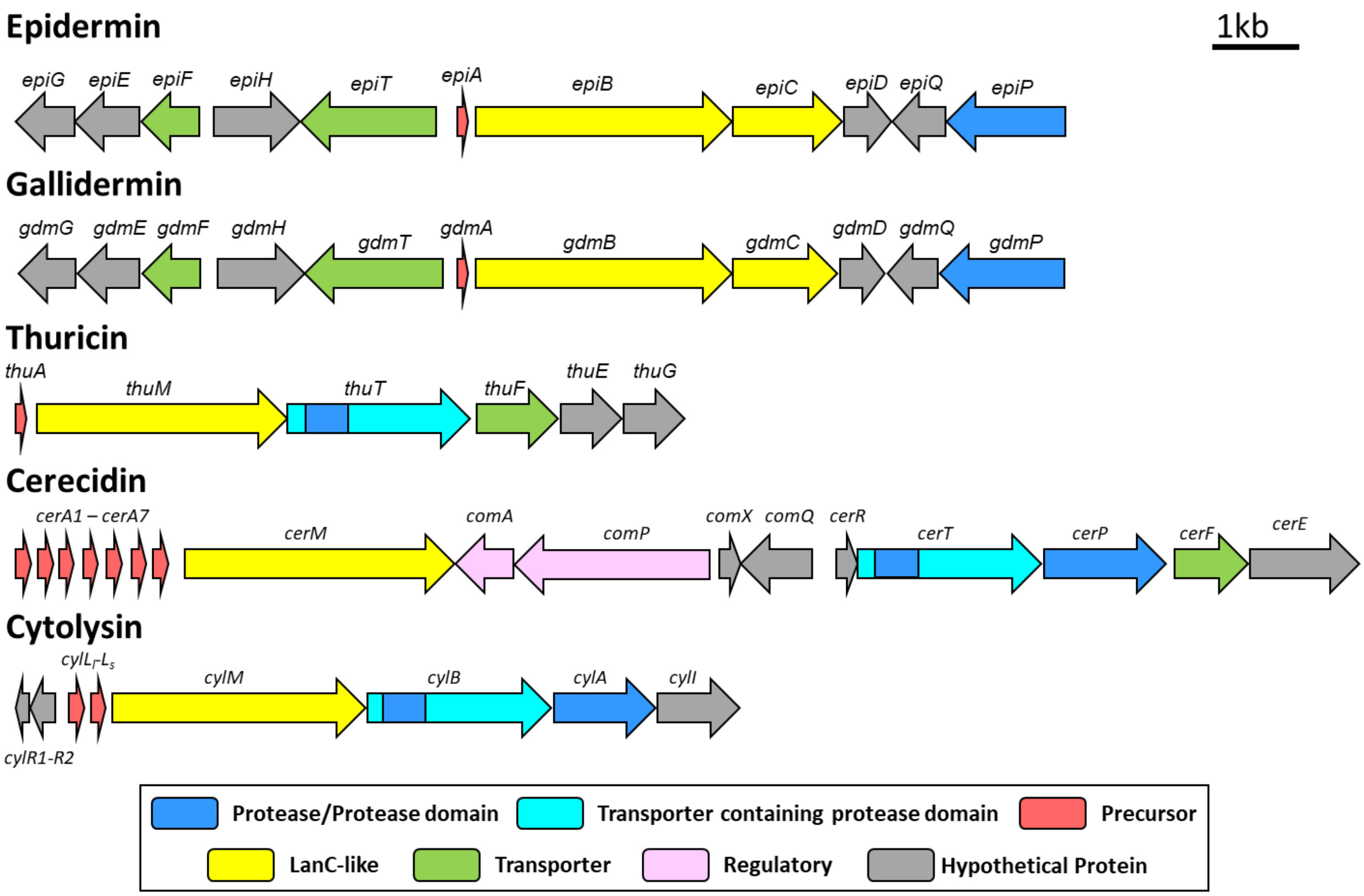

Five lanthipeptide BGCs producing epidermin, gallidermin, thuricin, cerecidin, and cytolysin, which were found to encode pathway-specific LanP and LanT-like proteases that are commonly known to be involved in class I and class II lanthipeptide biosynthesis. Genes encoding LanC-like proteins are represented in yellow, while genes encoding precursor peptides are represented in red, protease encoding genes in blue, transporter encoding genes in green, regulatory genes in pink, and genes encoding transporters which contain protease domains are represented in cyan with a small blue box to indicate the location of the protease domain.

Supplementary Figure 5: Class III lanthipeptide BGCs containing FlaA-like precursors

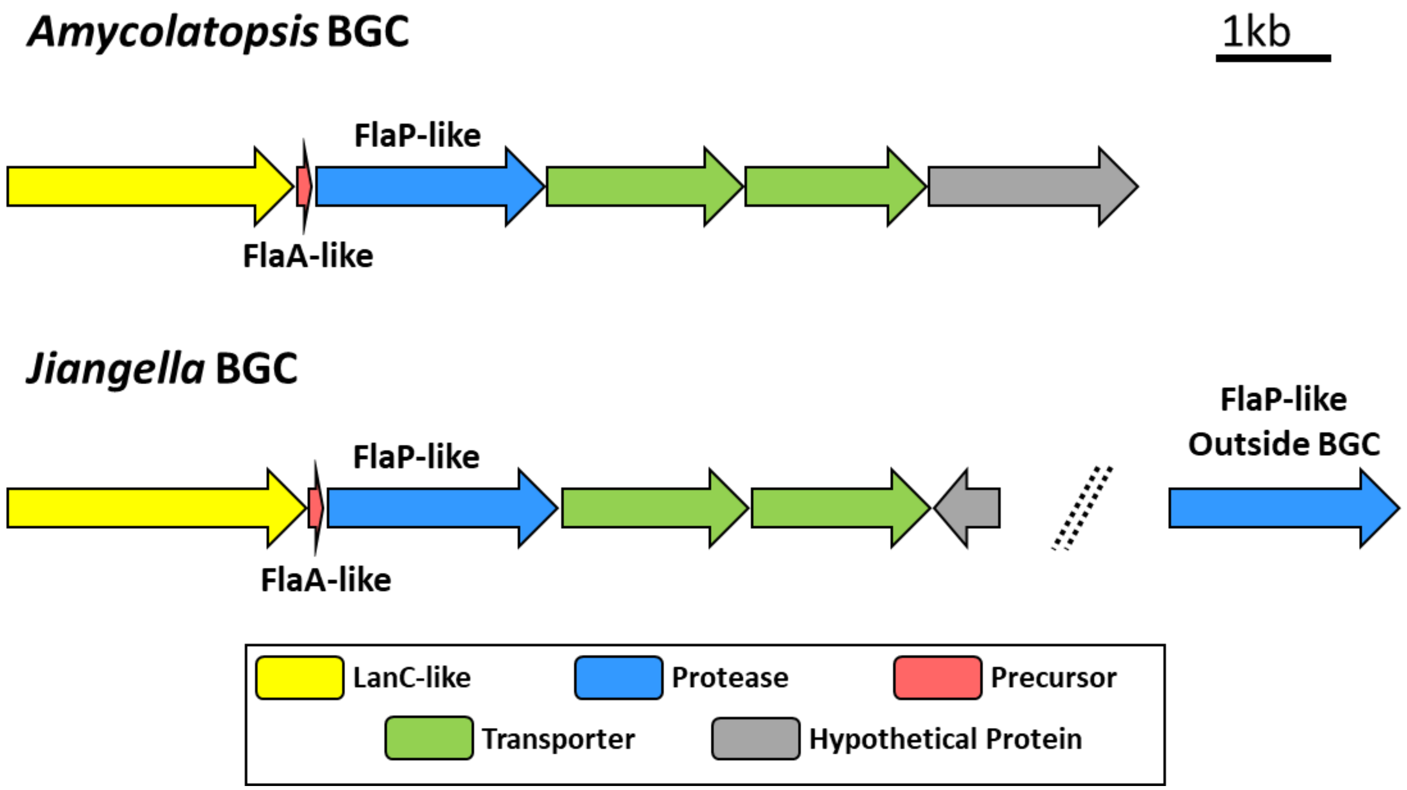

Class III lanthipeptide BGCs containing FlaA-like precursors and FlaP-like proteases were identified from the genera *Amycolatopsis* and *Jiangella*. An additional FlaP-like protease was detected outside of the BGC in the genus *Jiangella*. Genes encoding LanC-like (LanKC) proteins are represented in yellow, while genes encoding precursor peptides are represented in red, protease genes in blue, and transporter genes in green.

Supplementary Figure 6: Bacinapeptin A heterologous production and structure elucidation by MS/MS analysis

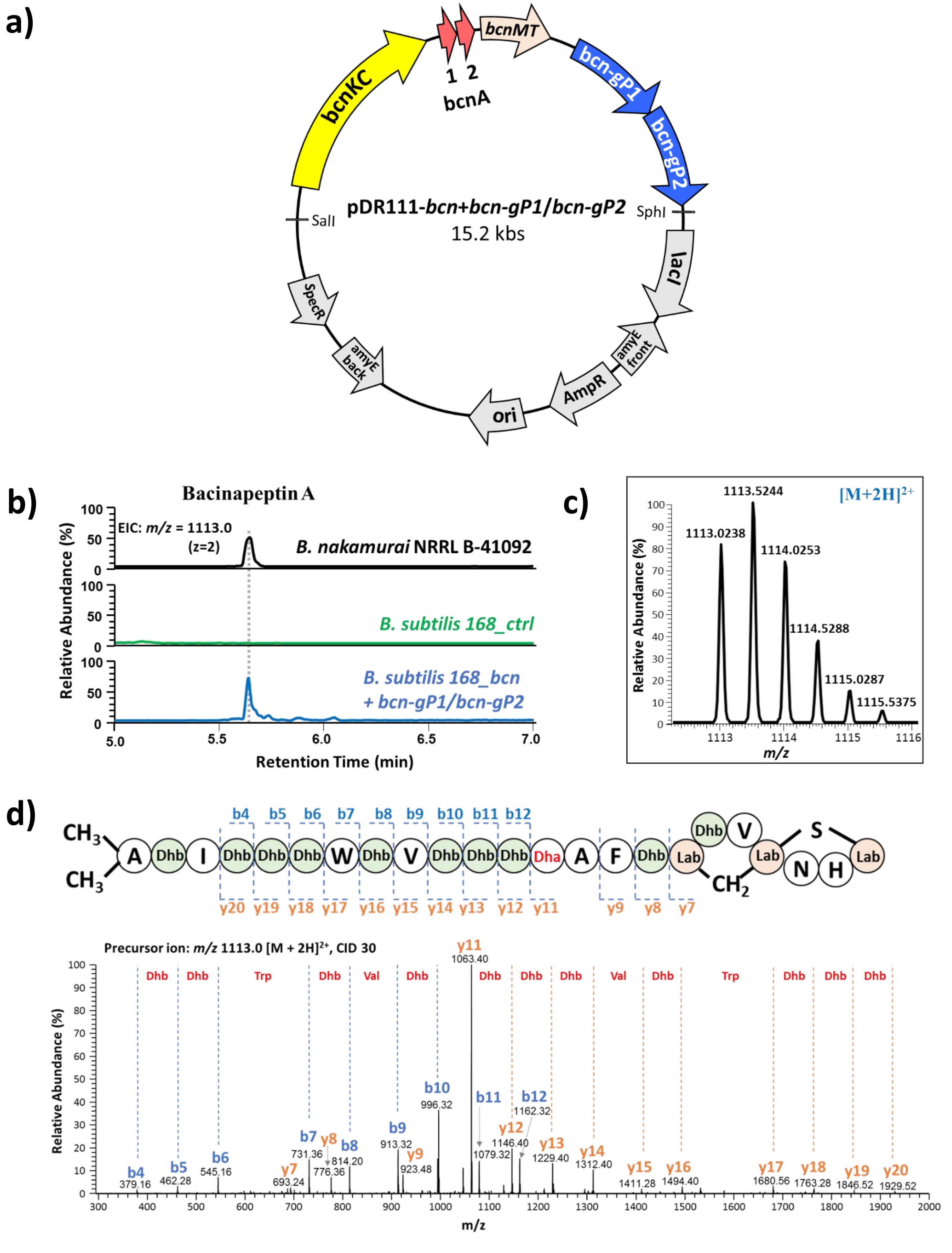

e)

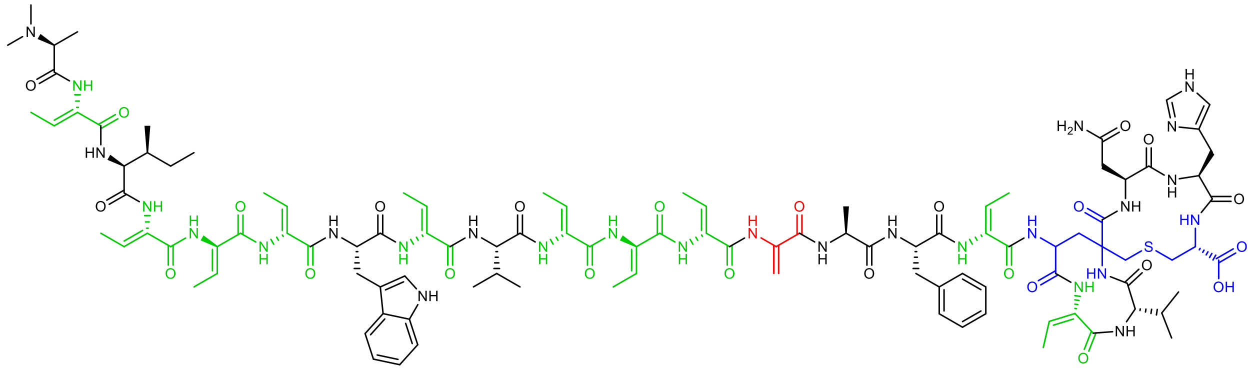

Heterologous expression in *Bacillus subtilis* 168 was used to connect the production of bacinapeptin A with the *bcn* BGC. **a)** Plasmid map of pDR111-*bcn*+*bcn-gP1*/*bcn-gP2* that was used for the heterologous expression of the *bcn* BGC in *B. subtilis* 168. Grey arrows indicate elements in the vector. Arrows with other colors show genes of *bcn* BGC and *bcn-gP1/bcn-*g*P2*. **b)** Extracted ion chromatograms (EIC) of extracts from three samples: *B. nakamurai* NRRL B-41092, *B. subtilis* 168_ctrl, and *B. subtilis* 168_*bcn*+*bcn-gP1*/*bcn-gP2* are overlaid for comparison. **c)** The doubly charged state of bacinapeptin A is provided which was detected at 1112.98 m/z. **d)** The amino acid sequence of bacinapeptin A with b and y ions marked as well as the location of the labionin ring. MS/MS was used to fragment bacinapeptin A to confirm the amino acid sequence by examination of the fragmentation patterns. The doubly charged precursor ion, *m/z* 1113.0, was selected for collision induced dissociation (CID) at 30 eV. Major fragment ions are annotated with their b or y ion identity and the amino acid residues deduced from fragment ions are labelled in red. **e)** Chemical structure of bacinapeptin A. Chemical formula: $C_{106}H_{141}N_{27}O_{25}S$. Dhb is in green, Dha is in red, and Ser and Cys involved in the labionin ring are in blue.

Supplementary Figure 7: Bacinapeptin B heterologous production and structure elucidation by MS/MS analysis

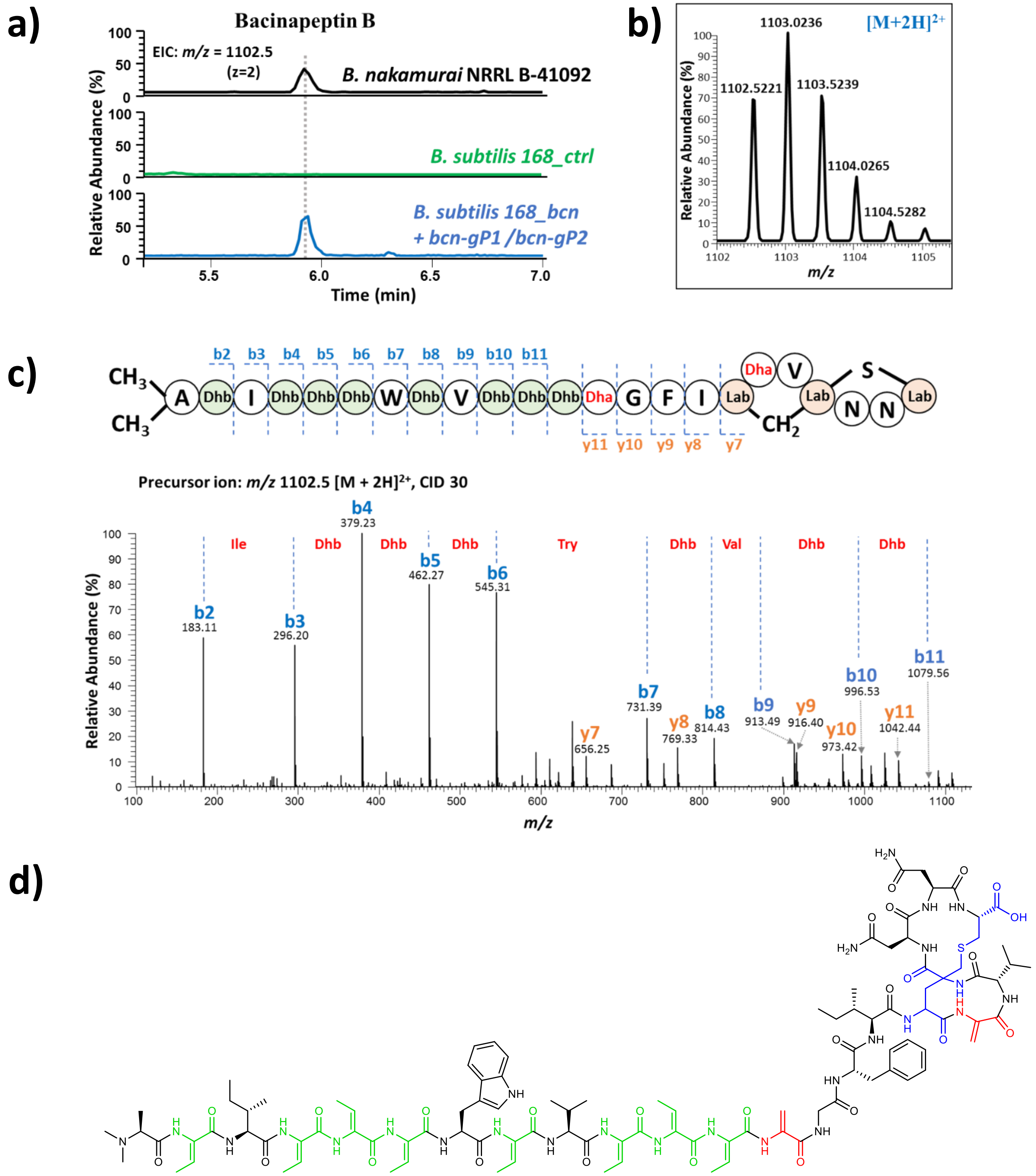

Heterologous expression in *B. subtilis* 168 was used to connect the production of bacinapeptin B with the *bcn* BGC. **a)** Extracted ion chromatograms (EIC) of extracts from three samples: *B. nakamurai* NRRL B-41092, *B. subtilis* 168_ctrl, and *B. subtilis* 168_*bcn*+*bcn-gP1*/*bcn-gP2* are overlaid for comparison. **b)** The doubly charged state of bacinapeptin B is provided which was detected at 1102.5 m/z. **c)** The amino acid sequence of bacinapeptin B with b and y ions marked as well as the location of the labionin ring. MS/MS was used to fragment bacinapeptin B to confirm the amino acid sequence by examination of the fragmentation patterns. The doubly charged precursor ion, *m/z* 1102.5, was selected for collision induced dissociation (CID) at 30 eV. Major fragment ions are annotated with their b or y ion identity and the amino acid residues deduced from fragment ions are labelled in red. **d)** Chemical structure of bacinapeptin B. Chemical formula: $C_{104}H_{142}N_{26}O_{26}S$. Dhb is in green, Dha is in red, and Ser and Cys involved in the labionin ring are in blue.

Supplementary Figure 8: Heterologous production of paenithopepins A-E

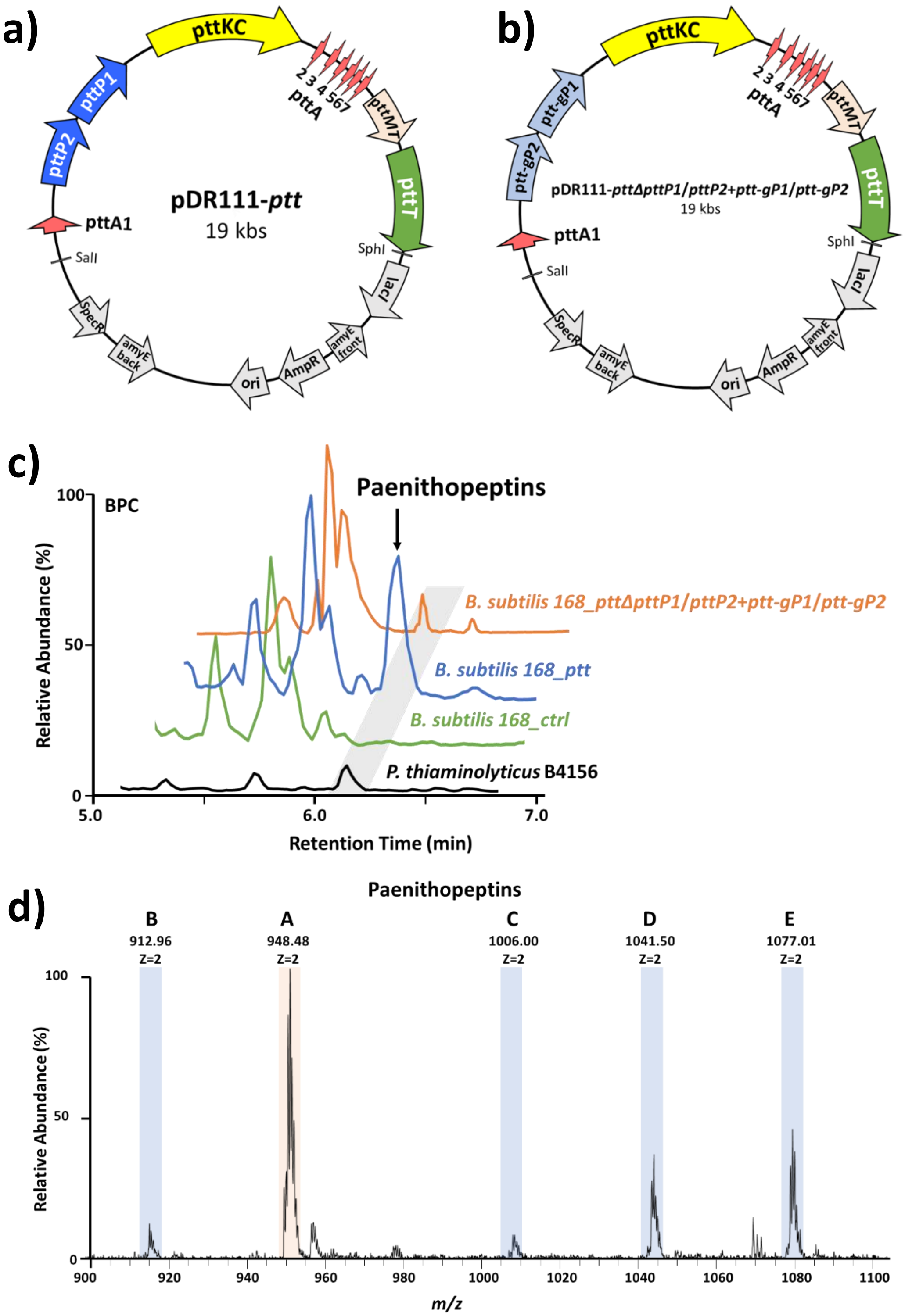

Heterologous expression in *B. subtilis* 168 was used to connect the production of paenithopeptins A-E with the *ptt* BGC. **a)** Plasmid map of pDR111-*ptt* for the heterologous expression of the *ptt* BGC in *B. subtilis* 168. Grey arrows indicate elements in the vector. Arrows in other colors show genes in the *ptt* BGC. **b)** Plasmid map of pDR111-*ptt*Δ*pttP1*/*pttP2*+*ptt-gP1*/*ptt-gP2*. The *pttP1 and pttP2 genes* were replaced by *ptt-gP1* and *ptt-gP2* genes (light blue arrows). **c)** Base peak chromatograms of extracts from four samples: *B. subtilis* 168_*ptt*, *B. subtilis* 168_ *ptt*Δ*pttP1*/*pttP2*+*ptt-gP1*/*ptt-gP2*, *B. subtilis* 168_ctrl, and *Paenibacillus thiaminolyticus* NRRL B-4156 are overlaid for comparison with the paenithopeptins peak highlighted. **d)** A selected mass spectral scan of the indicated peak (retention time 5.75-6.75 min) displays the doubly charged states of all paenithopeptins A-E.

Supplementary Figure 9: High-resolution mass spectra of paenithopeptin A.

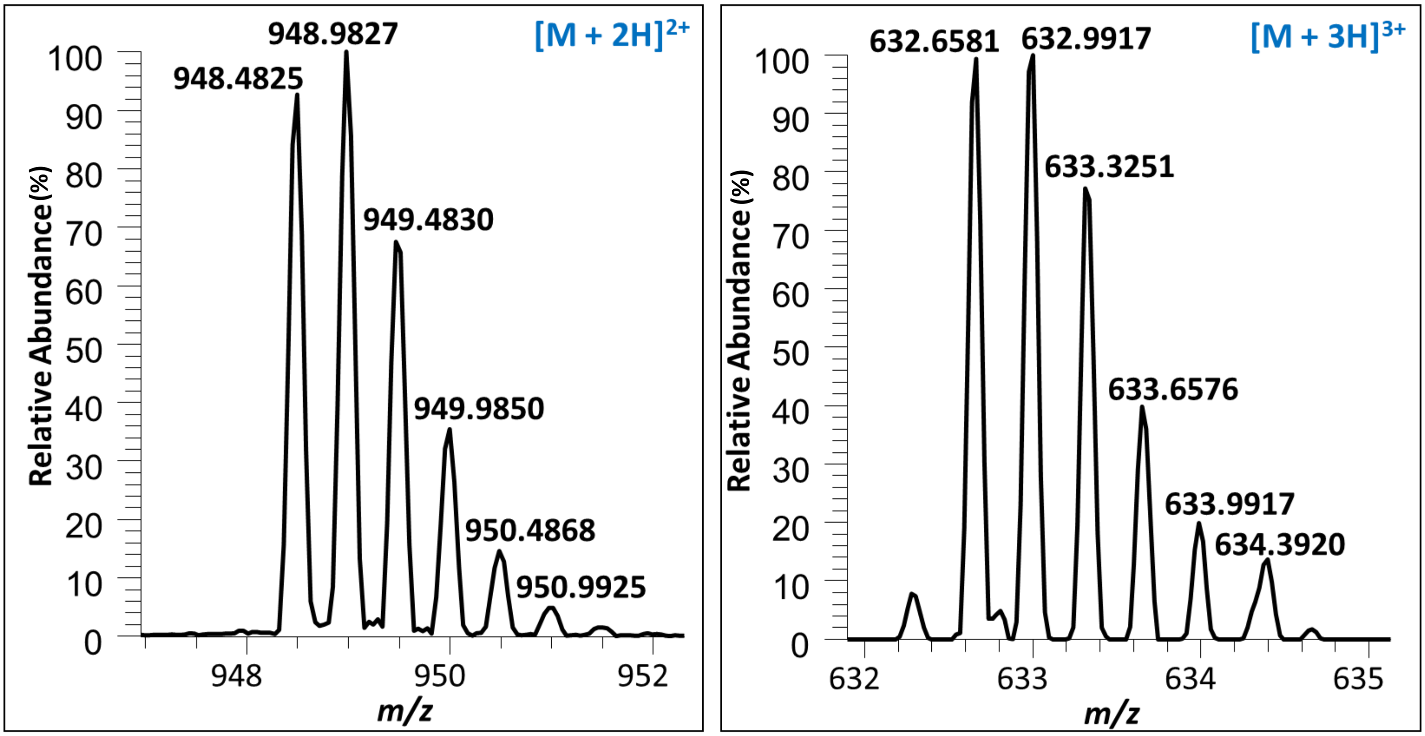

Paenithopeptin A was subjected to high-resolution mass spectrometry for accurate mass determination. The monoisotopic molecular weight of paenithopeptin A was calculated at 1894.9650 Da. The calculated mass for its doubly ($[M+2H]^{2+}$) and triply charged ($[M+3H]^{3+}$) states are 948.5 m/z and 632.67 m/z, respectively. Shown here are MS spectra representing the doubly charged and triply charged states of paenithopeptin A, found at 948.4825 m/z and 632.6581 m/z, respectively.

Supplementary Figure 10: MSMS spectrum of paenithopeptin A.

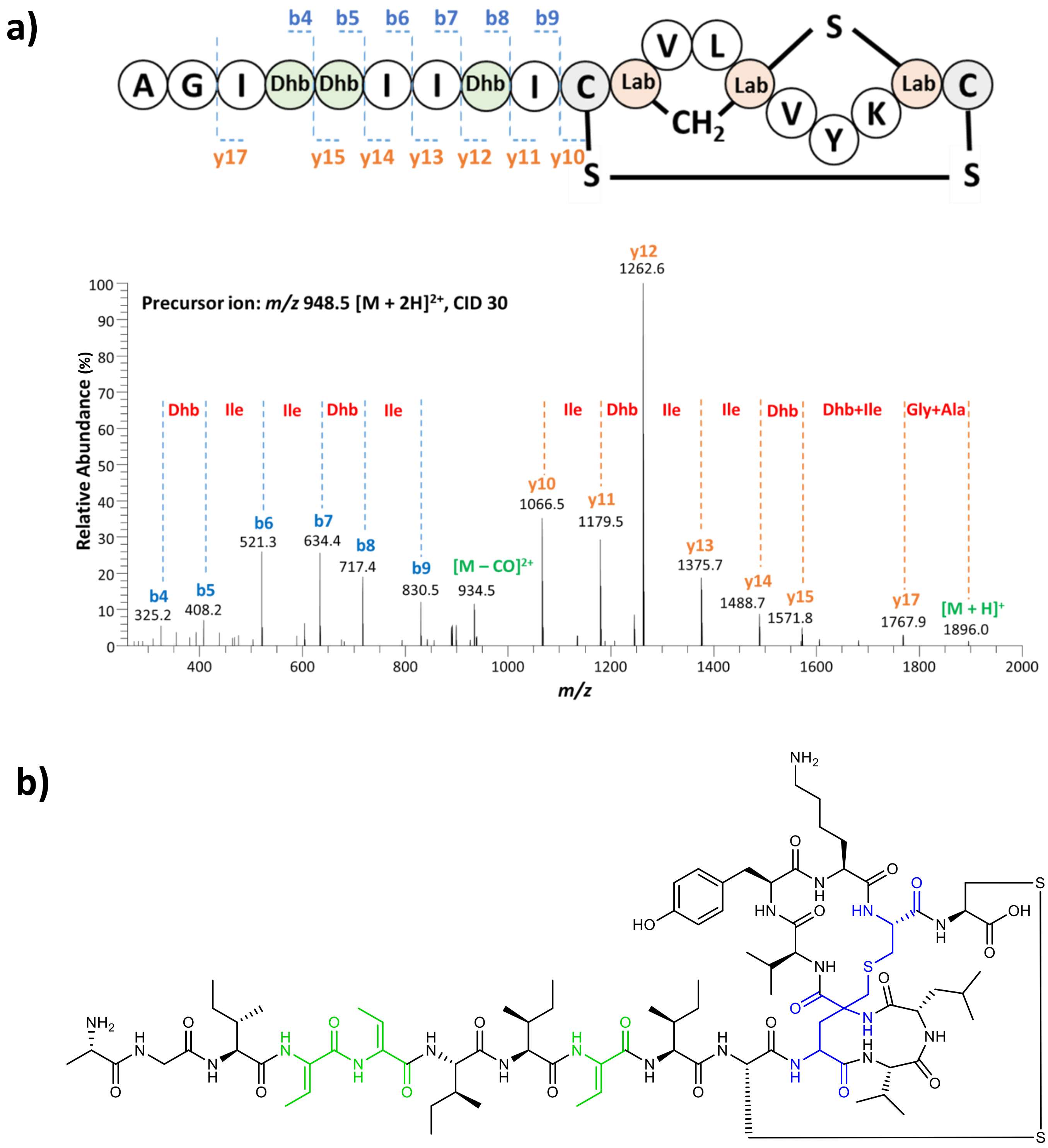

The amino acid sequence of paenithopeptin A **(a)** is presented here, with b and y ions marked as well as the location of the labionin ring and disulfide bond. MS/MS was used to fragment paenithopeptin A to confirm the amino acid sequence by examination of the fragmentation patterns. The doubly charged precursor ion, *m/z* 948.5, was selected for collision induced dissociation (CID) at 30 eV. Major fragment ions are annotated with their b or y ion identity and the amino acid residues deduced from fragment ions are labelled in red. **b)** Chemical structure of paenithopeptin A. Chemical formula: $C_{87}H_{138}N_{20}O_{21}S_3$. Dhb is in green, and Ser and Cys involved in the labionin ring are in blue.

Supplementary Figure 11: Chemical reduction for the determination of disulfide bond presence.

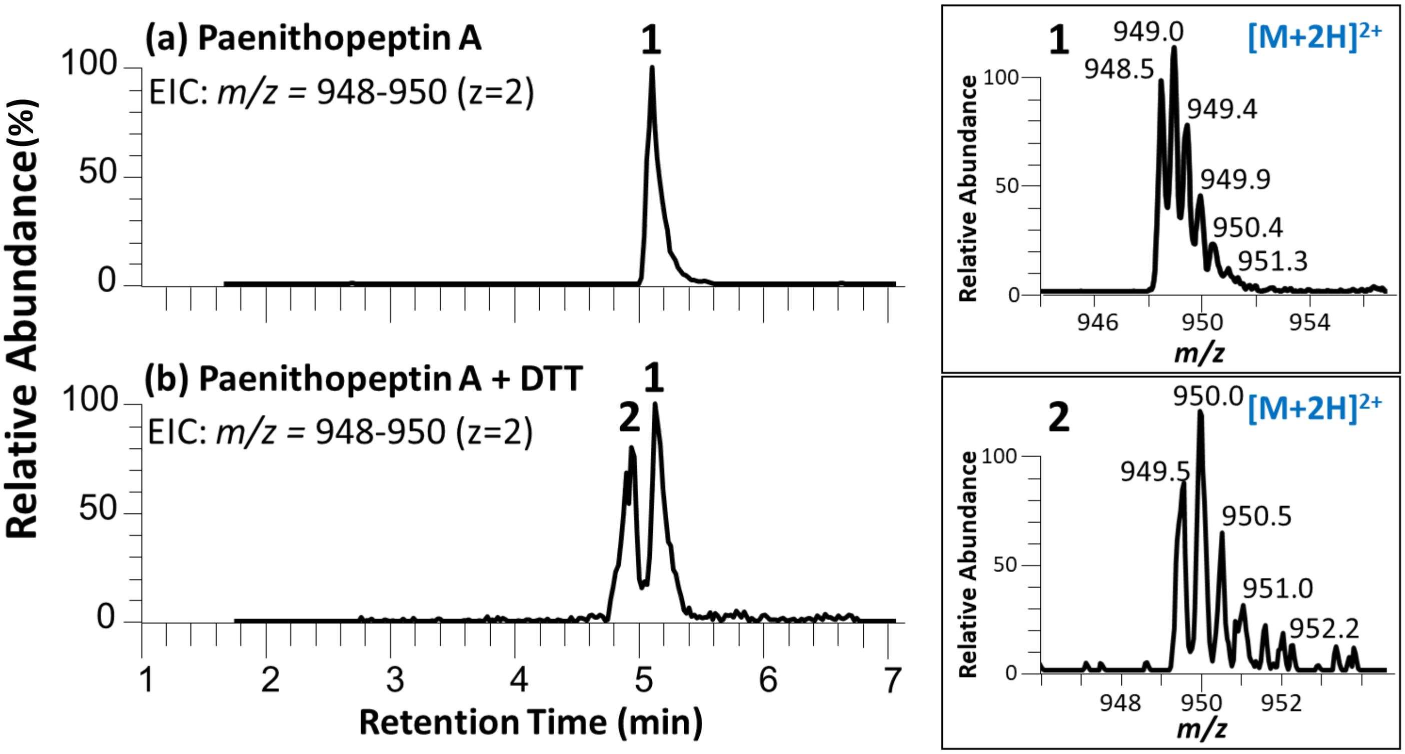

Inconsistency between observed accurate mass and predicted mass of paenithopeptin A suggested the presence of an additional structural feature. Hypothesizing the presence of a disulfide bond as seen in some other class III lanthipeptides, paenithopeptin A was incubated with dithiothreitol (DTT) for 12 h. Extracted ion chromatograms of m/z range 948 - 950 were used to detect the doubly charged state of paenithopeptin A (peak 1) **(a)** before DTT treatment, and **(b)** after DTT treatment (peak 2). Zoomed isotopic masses for labelled peaks are presented at right.

Supplementary Figure 12: MS/MS spectrum of paenithopeptin A after DTT treatment

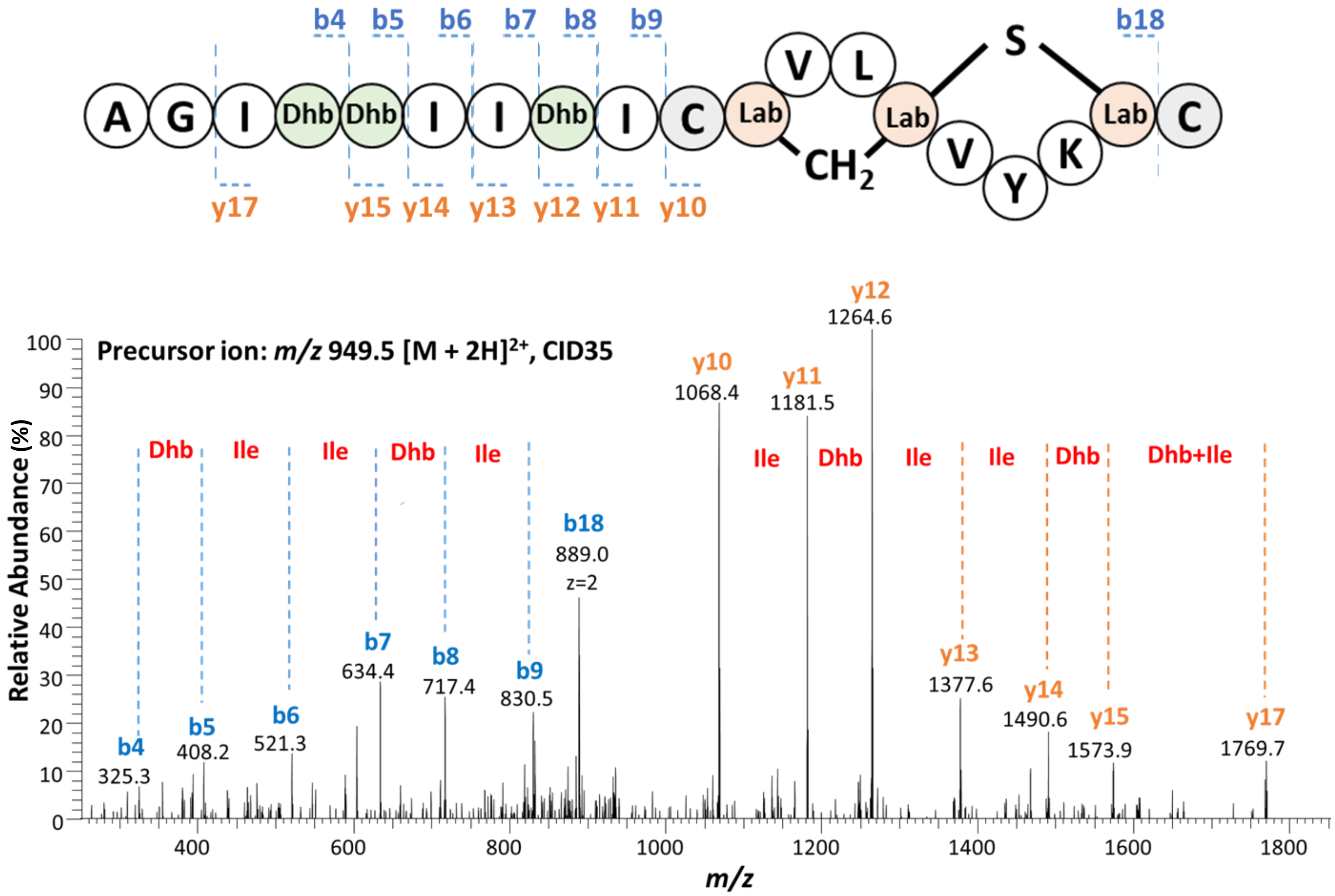

MS/MS was used to fragment paenithopeptin A after DTT treatment to confirm the presence of a disulfide bond by examination of the fragmentation patterns. The amino acid sequence of paenithopeptin A with b and y ions marked as well as the location of the labionin ring is presented above. The doubly charged precursor ion, *m/z* 948.5, was selected for collision induced dissociation (CID) at 35 eV. Major fragment ions are annotated with their b or y ion identity and the amino acid residues deduced from fragment ions are labelled in red. Appearance of b ion b18 compared to non-DTT treated paenithopeptin A indicated the reduction of the disulfide bond.

Supplementary Figure 13: Extracted ion chromatograms (EICs) of paenithopeptin A mutations.

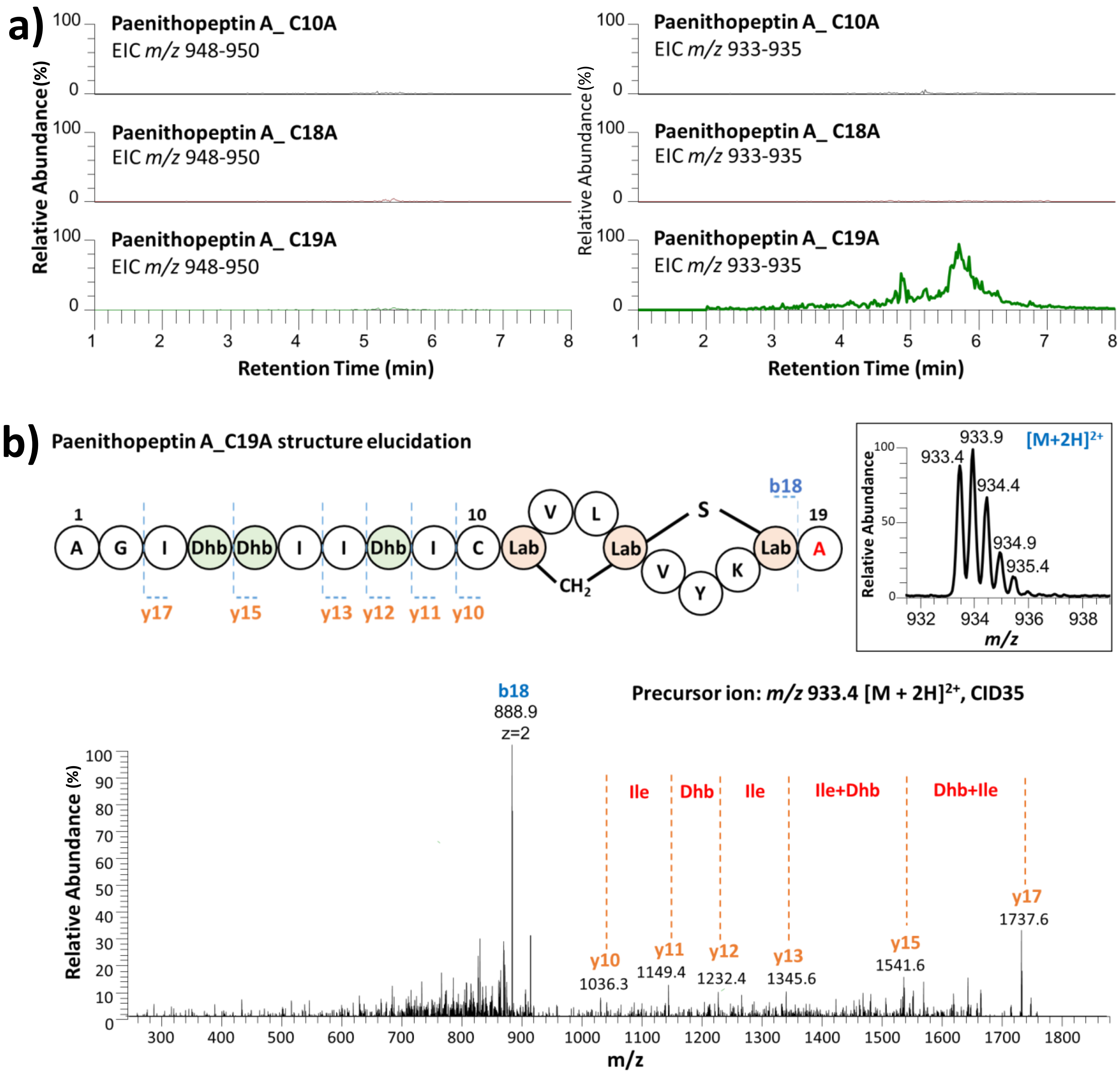

Point mutation to alanine of each cysteine residue possibly involved in disulfide bond formation was performed to provide secondary support to the presence and location of the disulfide bond. **a)** EICs in the m/z ranges of 949 - 950 and 933 - 935 are presented for each point mutation with only C19A showing a peak, representing paenithopeptin A with no disulfide bond and with one cysteine changed to alanine. The presence of this peak indicated the position of the disulfide bond as being between Cys10 and Cys19. **b)** The structure of paenithopeptin A_C19A is presented below with marked b ions, y ions, and labionin ring alongside the zoomed mass spectrum of its corresponding doubly charged state. Tandem MS/MS was used to confirm the amino acid sequence and loss of the disulfide bond. The doubly charged precursor ion *m/z* 933.4 was selected for collision induced dissociation (CID) at 35 eV. Major fragment ions are annotated with their b or y ion identity and amino acid residues deduced from fragment ion analysis are labelled in red.

Supplementary Figure 14: ^{1}H-NMR (500 MHz, DMSO-d_6) spectrum of paenithopeptin A

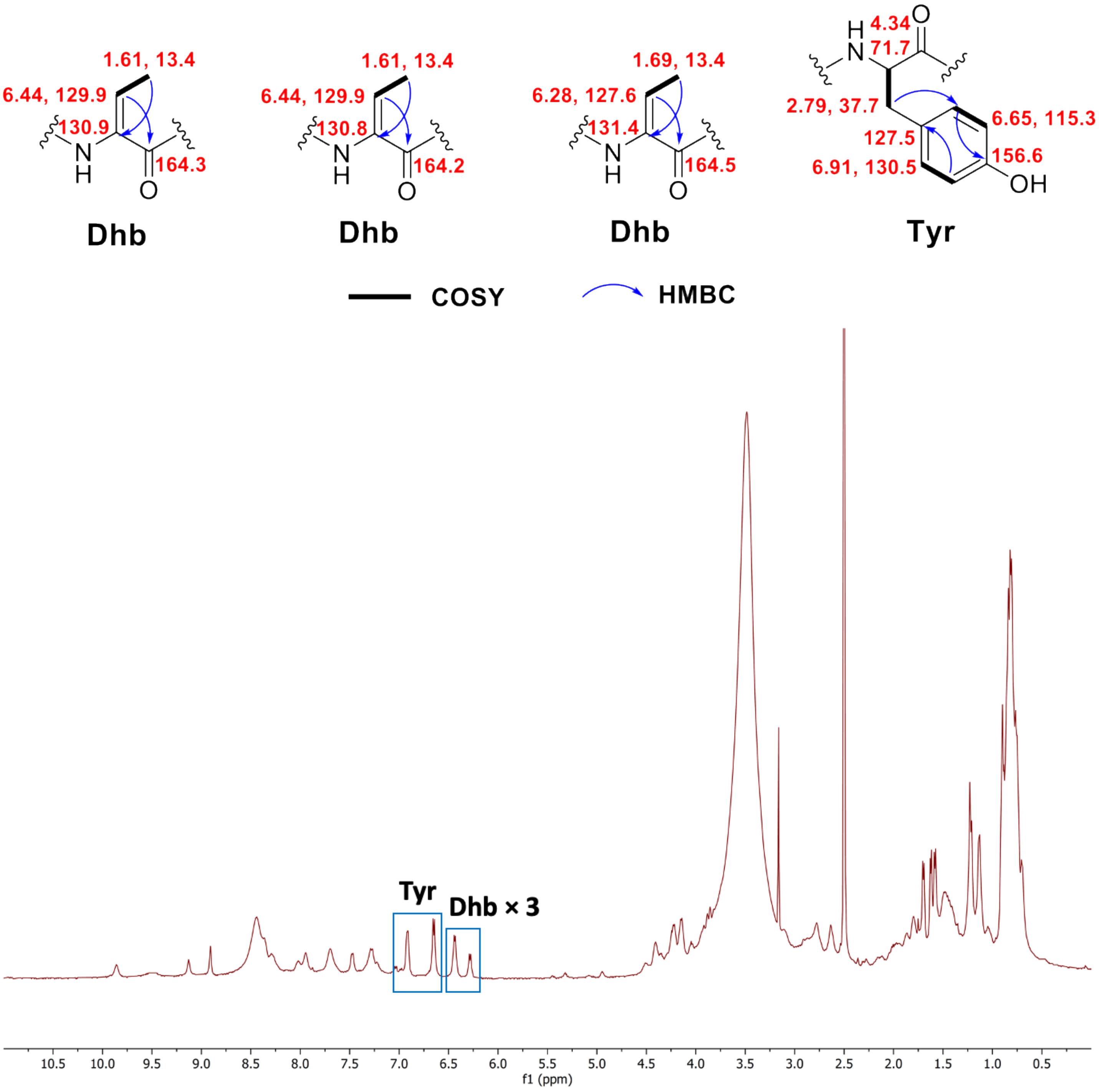

Supplementary Figure 15: HSQC NMR (500 MHz, DMSO-d_6) spectrum of paenithopeptin A

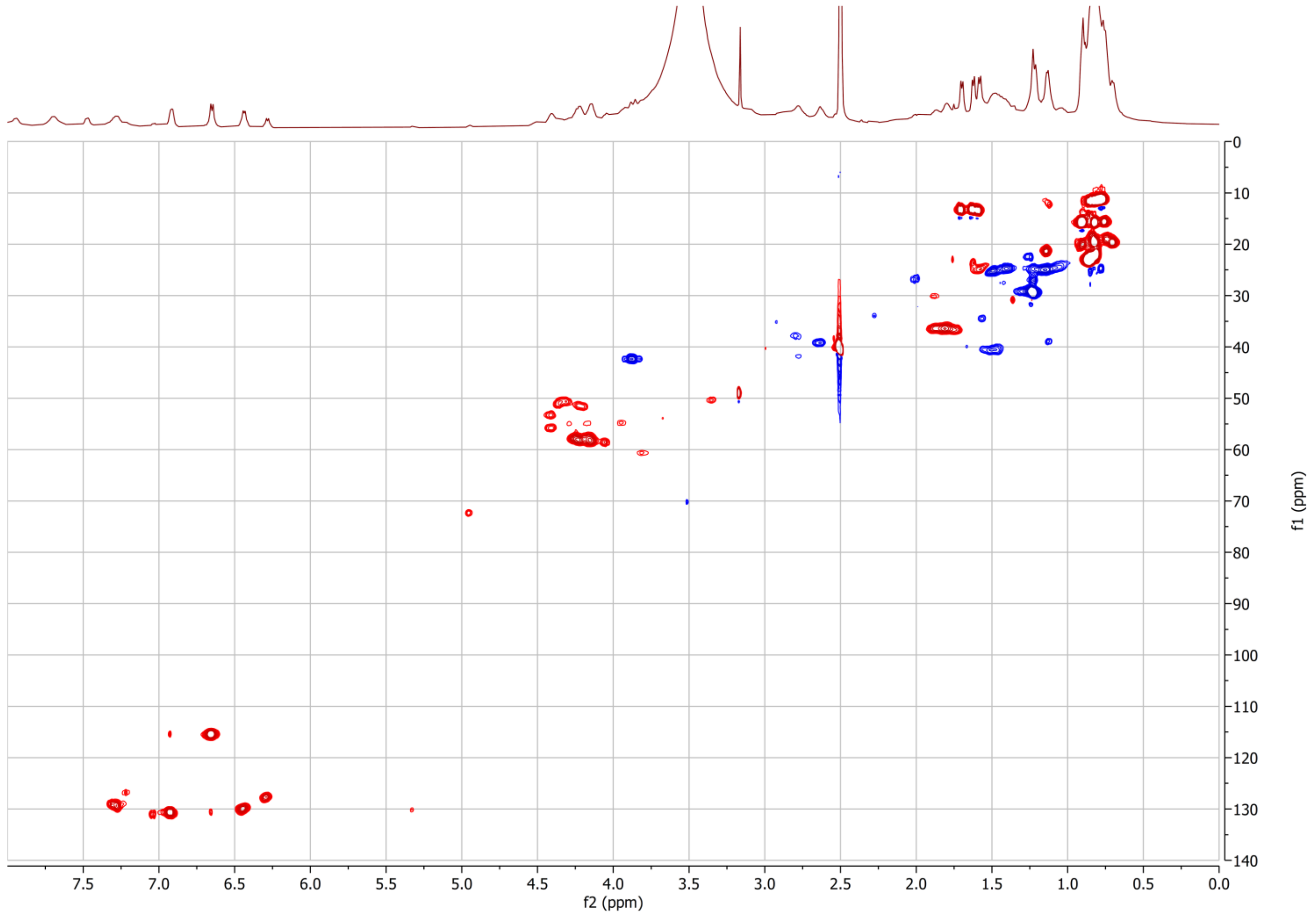

Supplementary Figure 16: HMBC NMR (500 MHz, DMSO-d_6) spectrum of paenithopeptin A

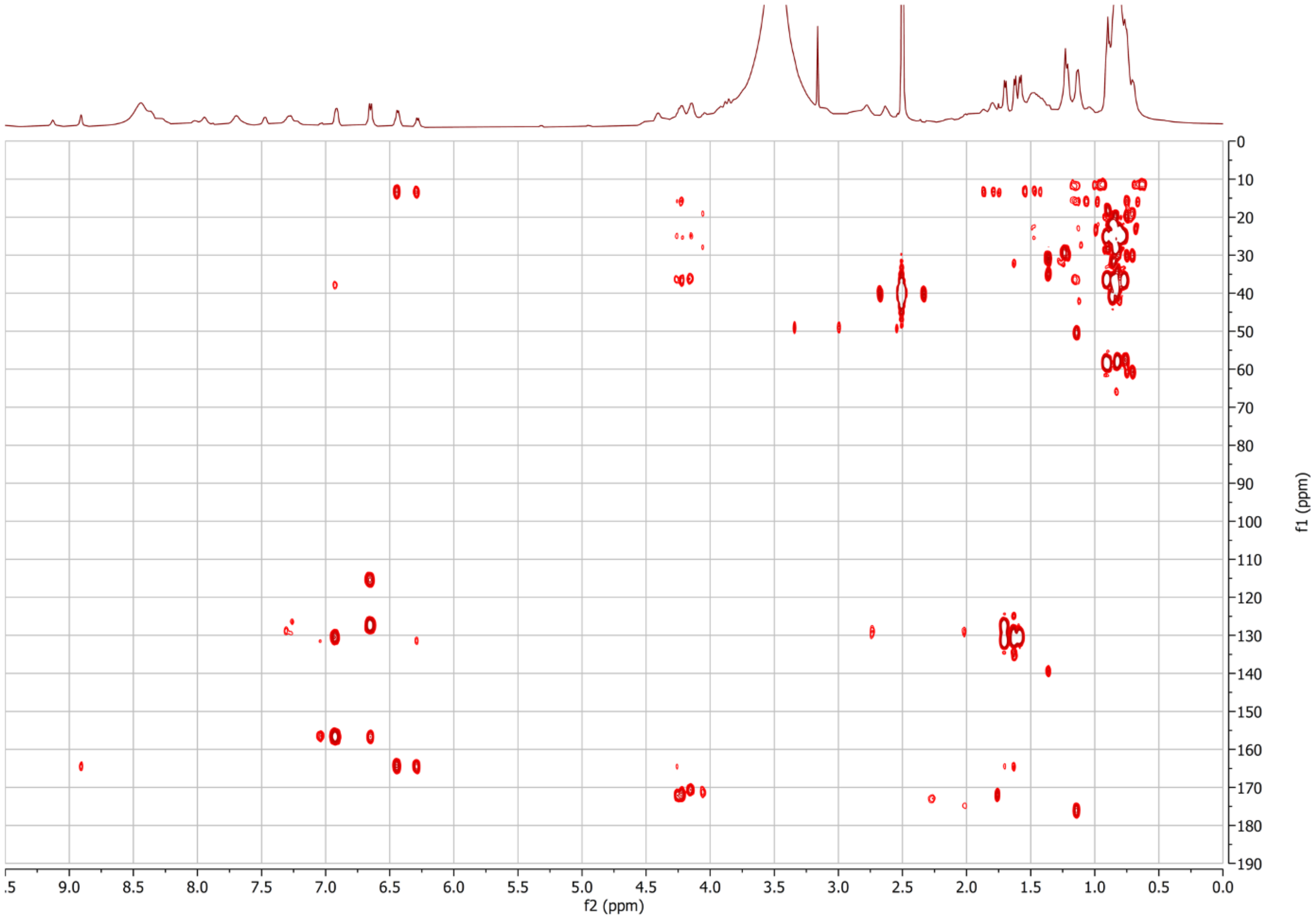

Supplementary Figure 17: ^{1}H-^{1}H COSY NMR (500 MHz, DMSO-d_6) spectrum of paenithopeptin A

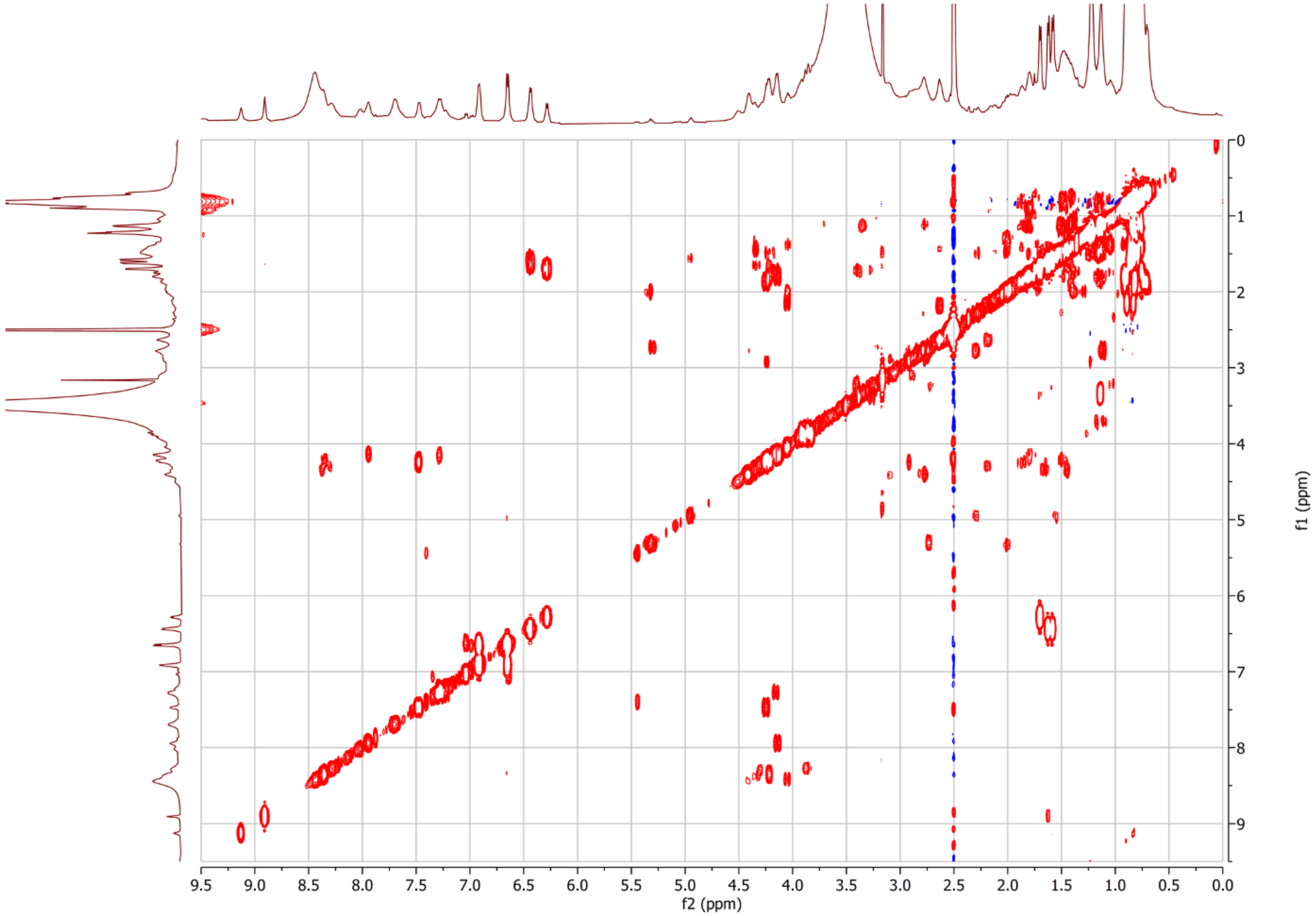

Supplementary Figure 18: ^{1}H-^{1}H TOCSY NMR (500 MHz, DMSO-d_6) spectrum of paenithopeptin A

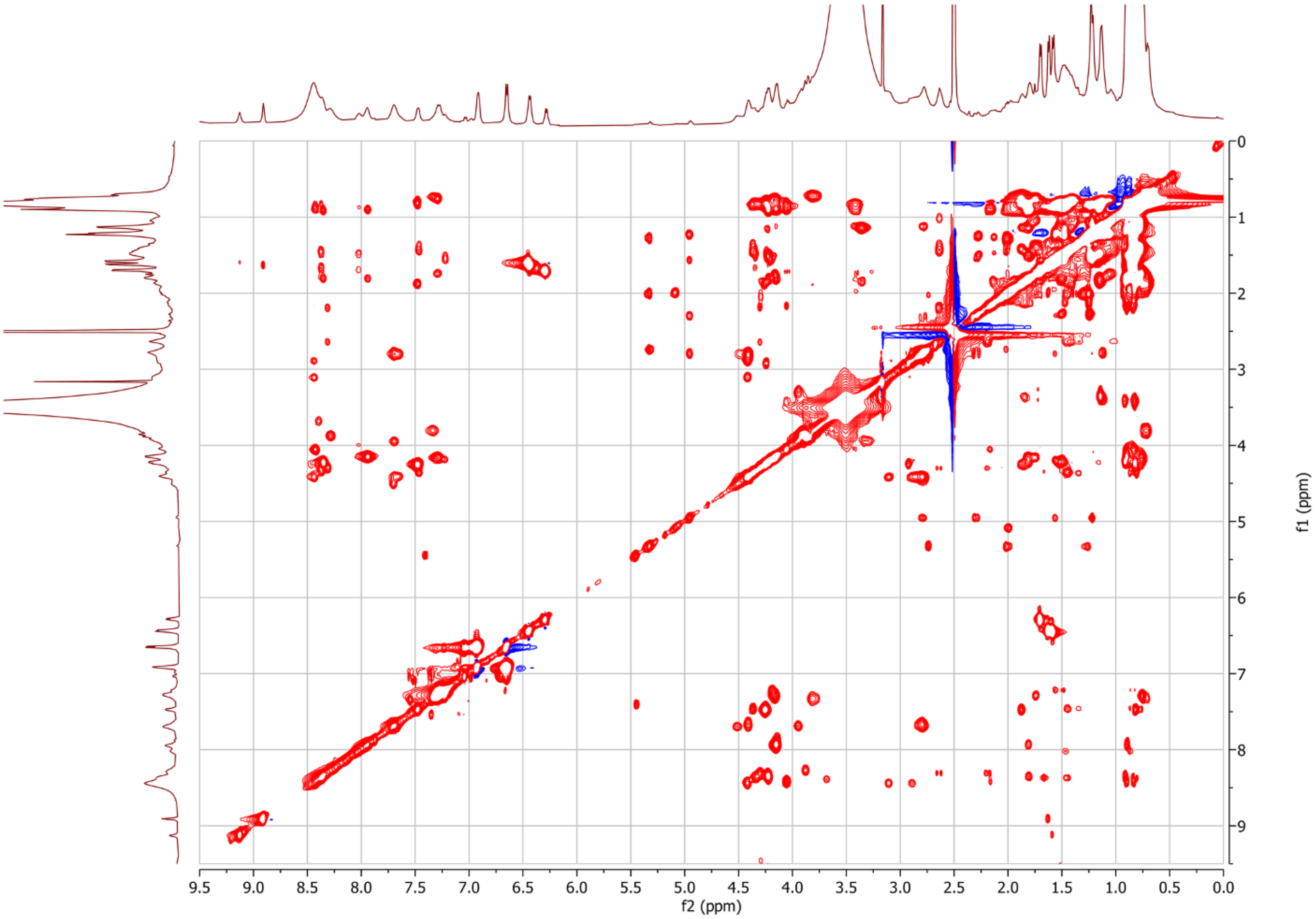

Supplementary Figure 19: HSQC-TOCSY NMR (500 MHz, DMSO-d_6) spectrum of paenithopeptin

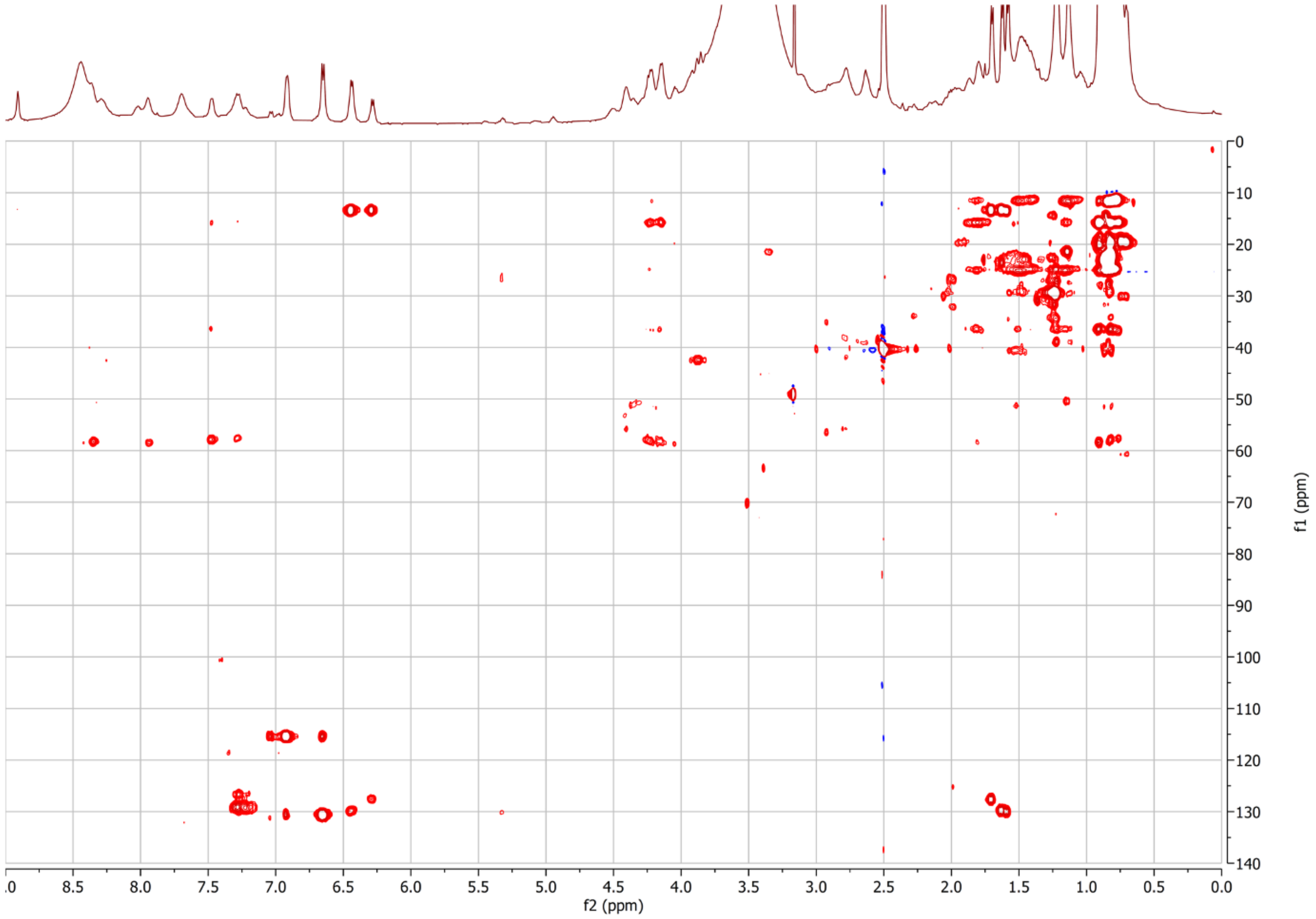

Supplementary Figure 20: ROESY NMR (500 MHz, DMSO-d_6) spectrum of paenithopeptin A

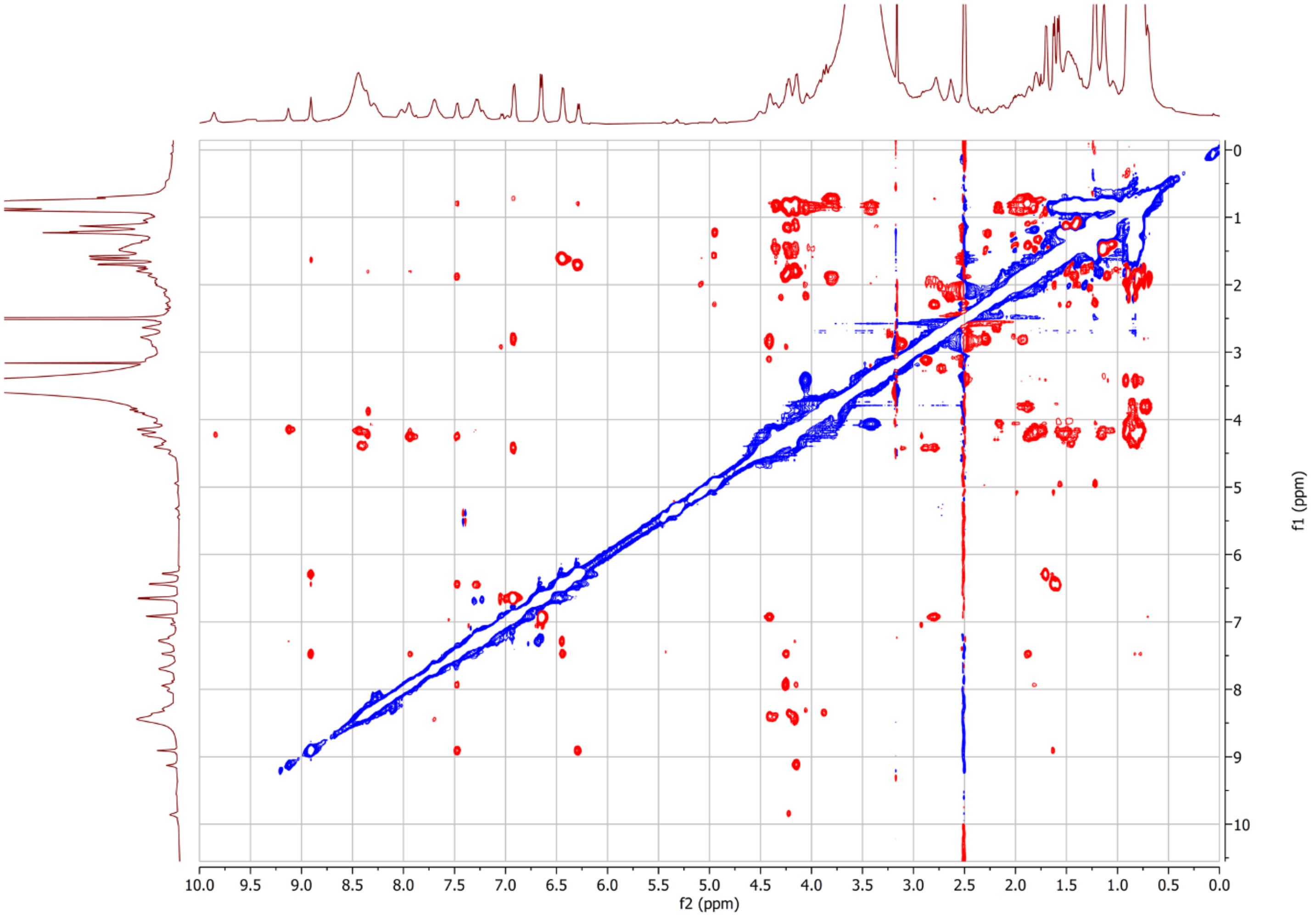

Supplementary Figure 21: MS/MS spectrum of paenithopeptin B

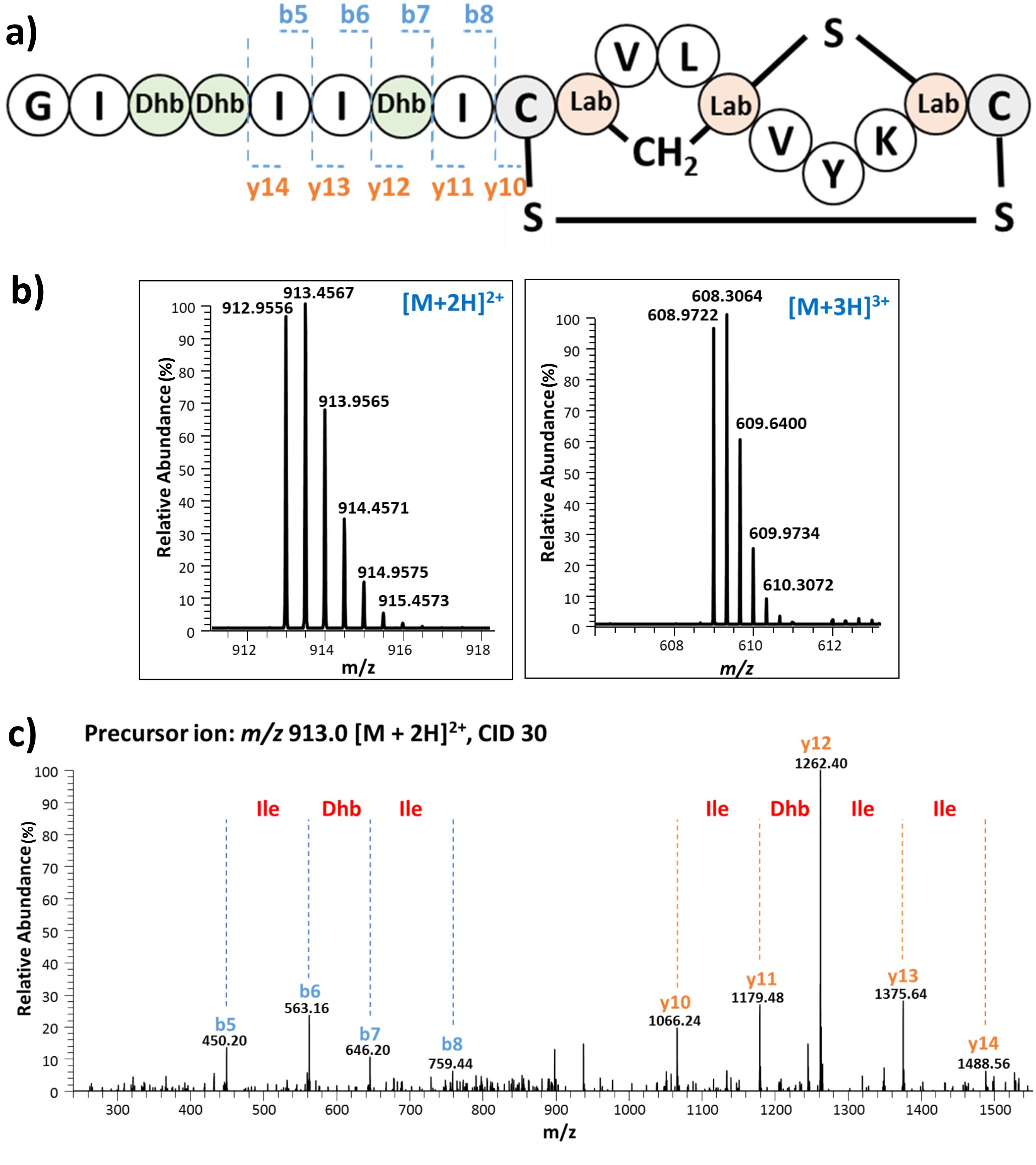

MS/MS was used to fragment paenithopeptin B to confirm the amino acid sequence by examination of the fragmentation patterns. **a)** The structure of paenithopeptin B with fragmentation points of corresponding b and y ions marked as well as the location of the labionin ring and disulfide bond. **b)** High resolution mass spectra representing the doubly and triply charged states of paenithopeptin B **c)**. The doubly charged precursor ion, *m/z* 913.0, was selected for collision induced dissociation (CID) at 30 eV. Major fragment ions are annotated with their b or y ion identity and the amino acid residues deduced from fragment ions are labelled in red.

Supplementary Figure 22: MS/MS spectrum of paenithopeptin C

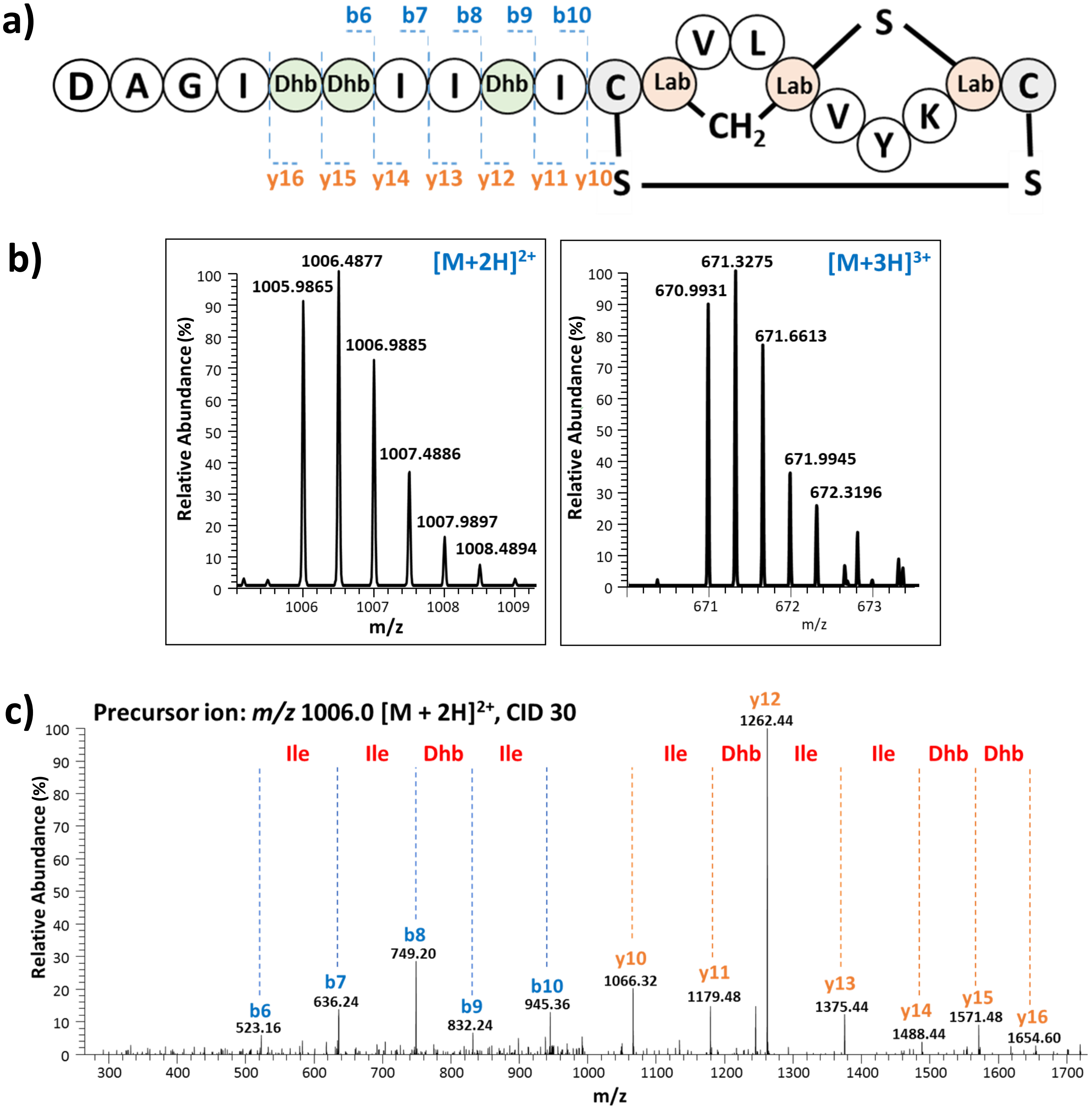

MS/MS was used to fragment paenithopeptin C to confirm the amino acid sequence by examination of the fragmentation patterns. **a)** The structure of paenithopeptin C with fragmentation points of corresponding b and y ions marked as well as the location of the labionin ring and disulfide bond. **b)** High resolution mass spectra representing the doubly and triply charged states of paenithopeptin C **c)**. The doubly charged precursor ion, *m/z* 1006.0, was selected for collision induced dissociation (CID) at 30 eV. Major fragment ions are annotated with their b or y ion identity and the amino acid residues deduced from fragment ions are labelled in red.

Supplementary Figure 23: MS/MS spectrum of paenithopeptin D

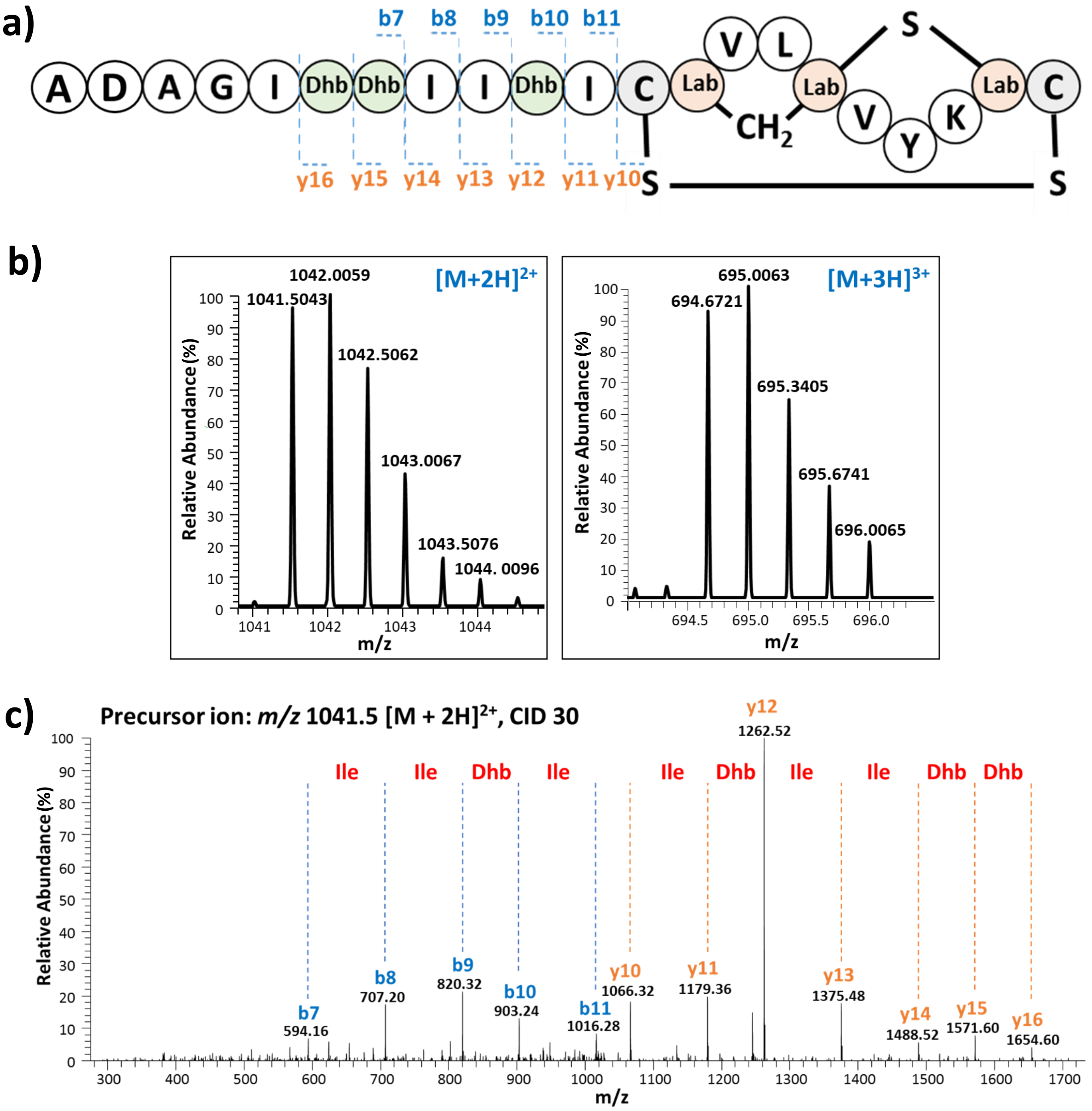

MS/MS was used to fragment paenithopeptin D to confirm the amino acid sequence by examination of the fragmentation patterns. **a)** The structure of paenithopeptin D with fragmentation points of corresponding b and y ions marked as well as the location of the labionin ring and disulfide bond. **b)** High resolution mass spectra representing the doubly and triply charged states of paenithopeptin D **c)**. The doubly charged precursor ion, *m/z* 1041.5, was selected for collision induced dissociation (CID) at 30 eV. Major fragment ions are annotated with their b or y ion identity and the amino acid residues deduced from fragment ions are labelled in red.

Supplementary Figure 24: MS/MS spectrum of paenithopeptin E

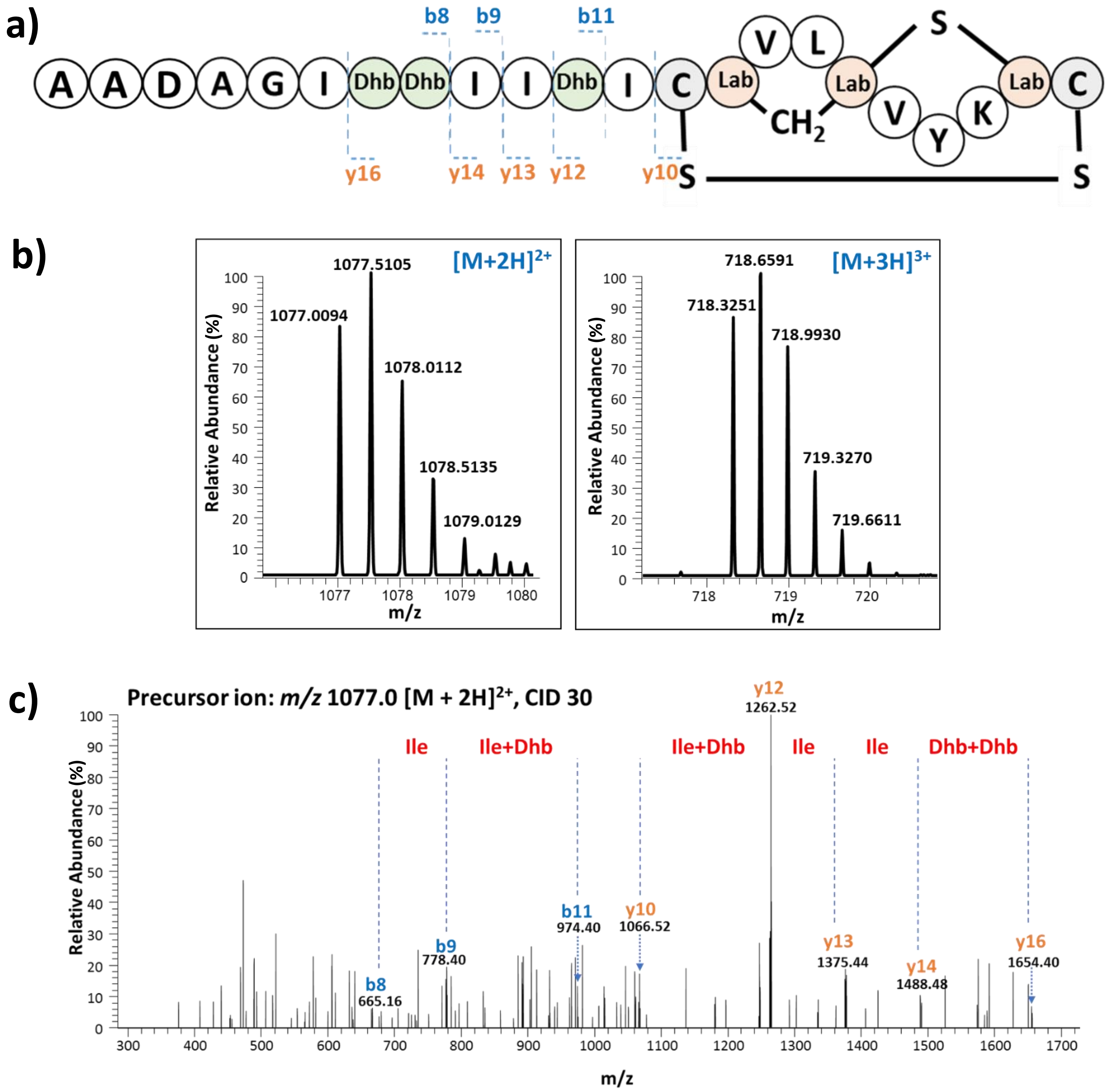

MS/MS was used to fragment paenithopeptin E to confirm the amino acid sequence by examination of the fragmentation patterns. **a)** The structure of paenithopeptin E with fragmentation points of corresponding b and y ions marked as well as the location of the labionin ring and disulfide bond. **b)** High resolution mass spectra representing the doubly and triply charged states of paenithopeptin E **c)**. The doubly charged precursor ion, *m/z* 1077.0, was selected for collision induced dissociation (CID) at 30 eV. Major fragment ions are annotated with their b or y ion identity and the amino acid residues deduced from fragment ions are labelled in red.

Supplementary Figure 25: Homology modeling of Bcn-gP1 and Bcn-gP2

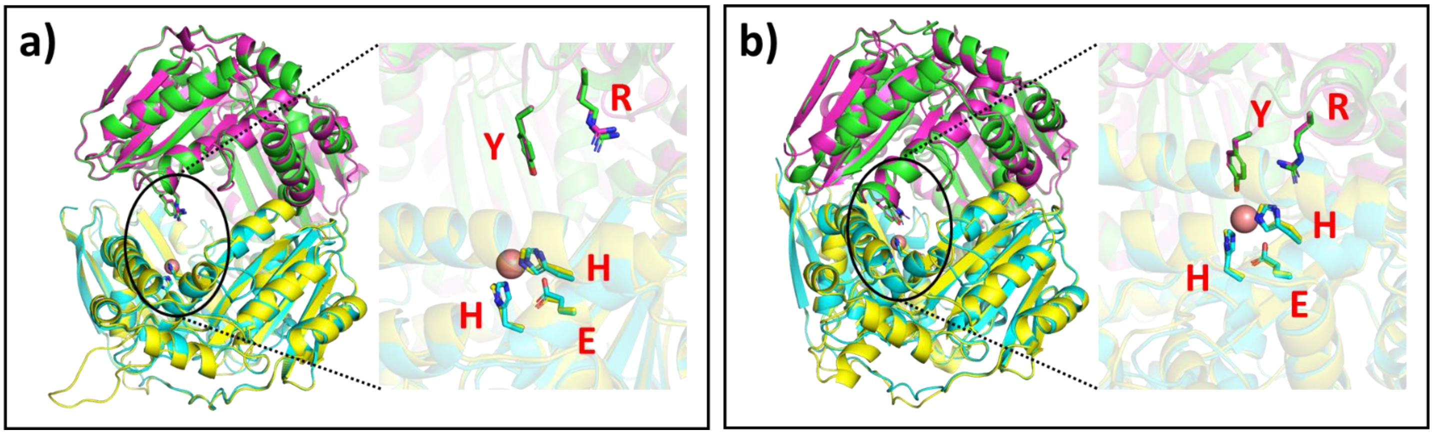

The sequences of Bcn-gP1 and Bcn-gP2 were used for homology modeling using the SWISS-MODEL platform. Sequences were aligned to the heterodimeric template sequence of Sph2681/Sph2682 (PDB code 3amj). Bcn-gP1 and Bcn-gP2 shared 28% and 30% sequence similarity with Sph2682 and Sph2681, respectively. Alignment of the heterodimeric model to the template structure in the open conformation revealed 0.43 RMSD for Bcn-gP2(yellow)/Sph2681(cyan) and 9.69 RMSD for Bcn-gP1(green)/Sph2682(pink). In the closed conformation, alignment revealed 0.40 RMSD for Bcn-gP2(yellow)/Sph2681(cyan) and 9.88 RMSD for Bcn-gP1(green)/Sph2682(pink). In both representations, inset zoomed views indicate residues of HxxEH motif and R/Y pair displayed in sticks and highlighted in red. **(a)** Models of Bcn-gP1/Bcn-gP2 overlaid on Sph2681/Sph2682 in the open conformation **(b)** Models of Bcn-gP1/Bcn-gP2 overlaid on Sph2681/Sph2682 in the closed conformation.

Supplementary Figure 26: Homology modeling of PttP1 and PttP2

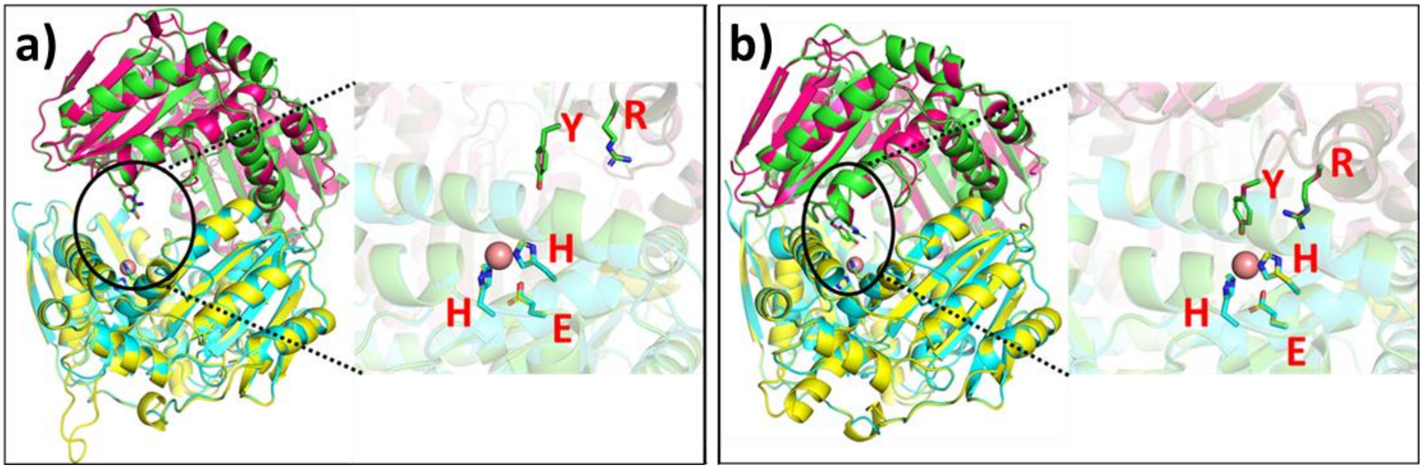

The sequences of PttP1 and PttP2 were used for homology modeling using the SWISS-MODEL platform. Sequences were aligned to the heterodimeric template sequence of Sph2681/Sph2682 (PDB code 3amj). PttP1 and PttP2 shared 29% and 30% sequence similarity with Sph2682 and Sph2681, respectively. Modeling of the heterodimeric structure revealed 0.17 RMSD for PttP2(yellow)/Sph2681(cyan) and 0.55 RMSD for PttP1(green)/Sph2682(pink). Alignment of the heterodimeric model to the template structure in the open conformation revealed 0.17 RMSD for PttP2(yellow)/Sph2681(cyan) and 0.55 RMSD for PttP1(green)/Sph2682(pink). In the closed conformation, alignment revealed 0.17 RMSD for PttP2(yellow)/Sph2681(cyan) and 0.49 RMSD for PttP1(green)/Sph2682(pink). In both representations, inset zoomed views indicate residues of HxxEH motif and R/Y pair displayed in sticks and highlighted in red. **(a)** Models of PttP1/PttP2 overlaid on Sph2681/Sph2682 in the open conformation **(b)** Models of PttP1/PttP2 overlaid on Sph2681/Sph2682 in the closed conformation.

Supplementary Figure 27: Pull-down assay and in vivo proteolytic activity of PttP1/PttP2

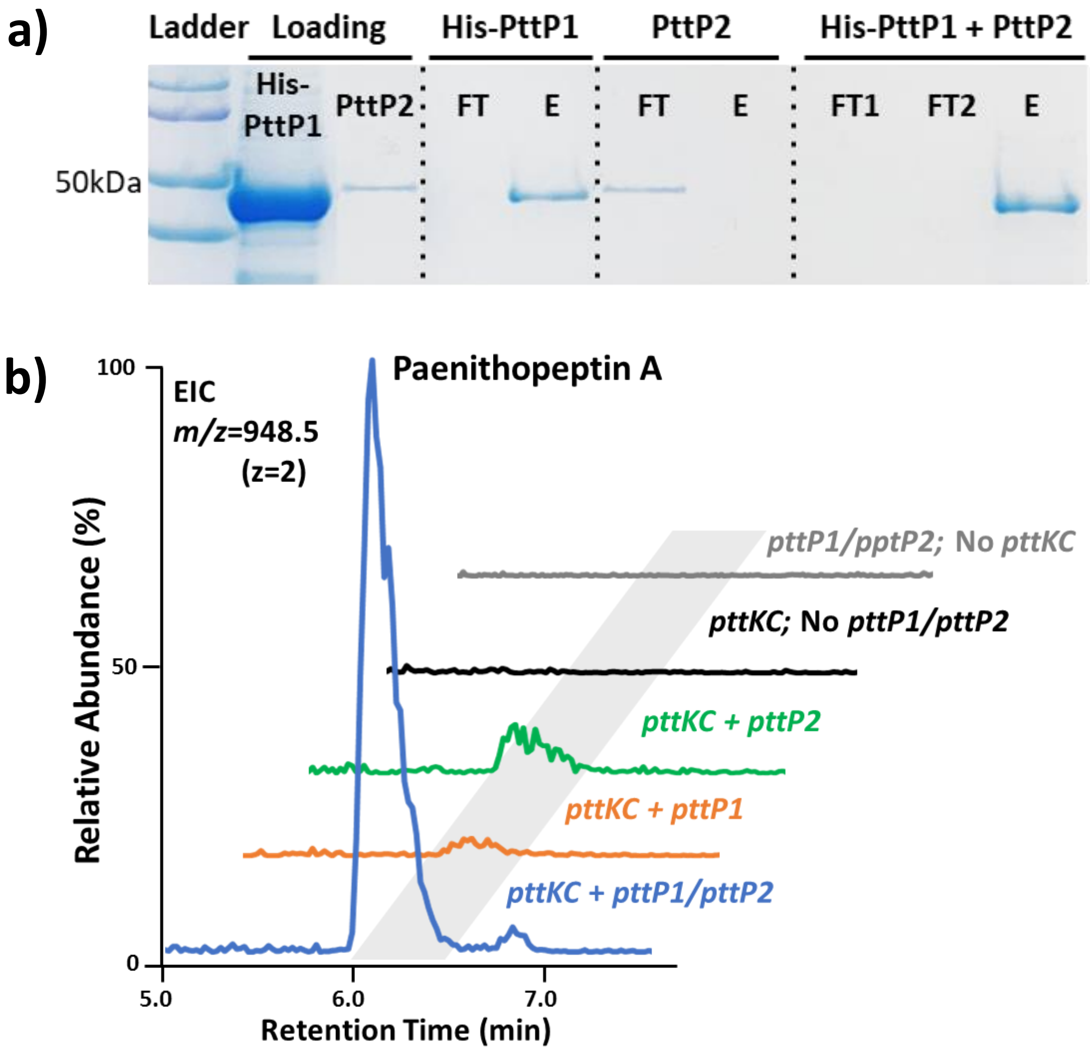

a) A pull-down assay showing protein-protein interaction between PttP1 and PttP2. His$_8$-tag-free PttP2 was immobilized in the nickel affinity column only when His$_8$-tagged PttP1 was present, as reflected by examining the flowthrough using sodium dodecyl sulfate polyacrylamide gel electrophoresis (SDS-PAGE). The experiment was repeated three times independently with similar results. FT: flowthrough; E: elute. **b)** Different combinations of four genes, *pttA1*, *pttKC*, *pttP1*, and *pttP2* were constructed into a series of pDR111-based integrative plasmids and expressed in an engineered *B. subtilis* 168 heterologous expression host lacking the native *pttP1/pttP2* homologs *ymfF*/*ymfH*. Extracted ion chromatograms (EIC) of m/z 948.5 ($[M+2H]^{2+}$), representing the doubly charged state of paenithopeptin A, are overlaid for comparison. In blue, *pttKC*, *pttP1*, and *pttP2* were all required for full production. In orange, expression of *pttKC* and *pttP1* resulted in a barely detectable amount of production. In green, *pttKC* and *pttP2* were enough for minor production. In black, lack of *pttP1/pttP2* protease pair resulted in no production. In grey, lack of *pttKC* resulted in no production.

Supplementary Figure 28: Different efficiencies of Prot_819/Prot_176 proteases against PttKC-modified PttA1

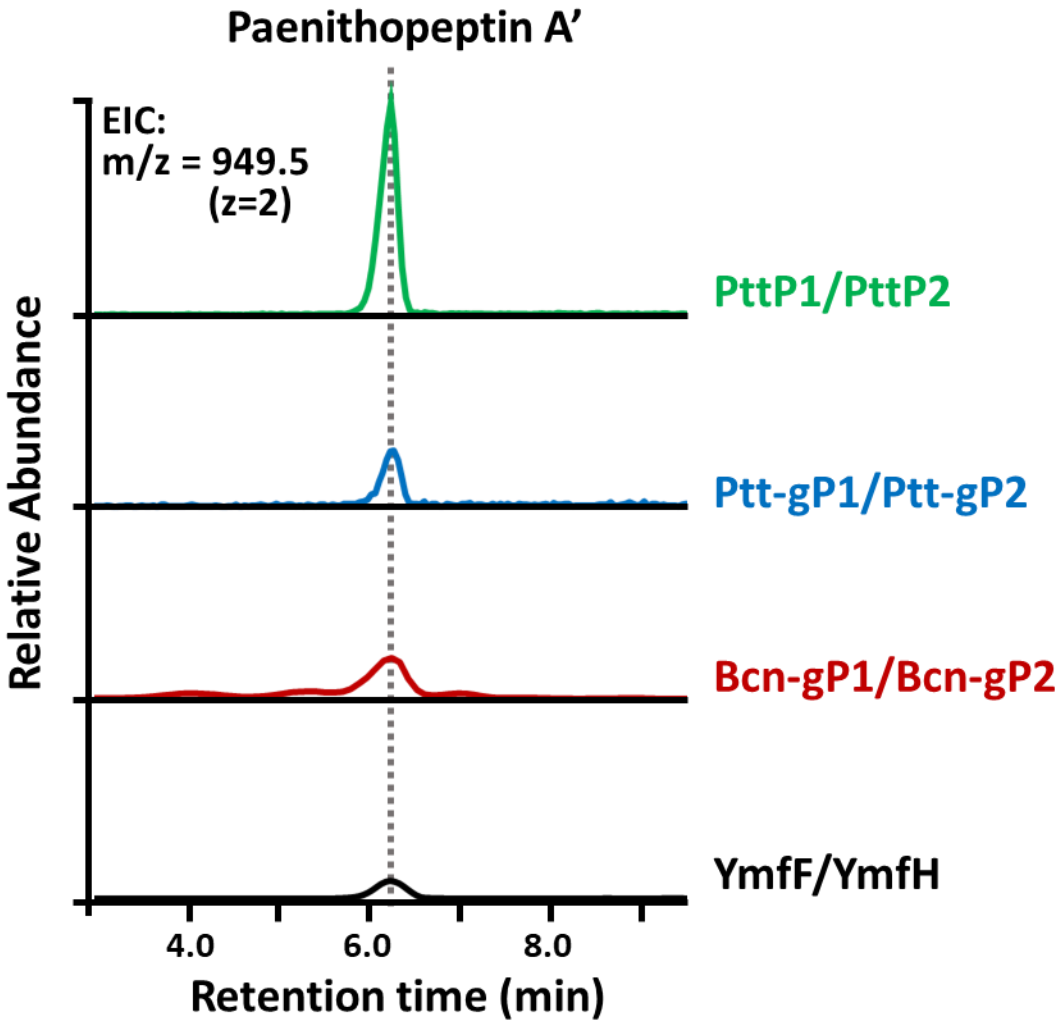

Proteolytic efficiency of Prot_819/Prot_176 proteases was compared using in vitro proteolysis of PttKC-modified PttA1. Extracted ion chromatograms (EIC) of m/z 949.5 ($[M+2H]^{2+}$), representing the doubly charged state of paenithopeptin A′, are overlaid for comparison. In green, production of paenithopeptin A′ with native PttP1/PttP2 proteases. In blue, Ptt-gP1/Ptt-gP2 found in the genome of *P. thiaminolyticus* NRRL B-4156, exhibited proteolysis with less efficiency compared to PttP1/PttP2. In red, Bcn-gP1/Bcn-gP2 detected in *B. nakamurai* NRRL B-41092, containing another Pre_24-encoding lanthipeptide BGC, demonstrated proteolytic activity towards PttA1 with less efficiency than native PttP1/PttP2. In black, YmfF/YmfH found in the *B. subtilis* 168 heterologous host, showed some proteolytic activity towards PttA1.

Supplementary Figure 29: Different efficiencies of Prot_819/Prot_176 proteases against BcnKC-modified BcnA1.

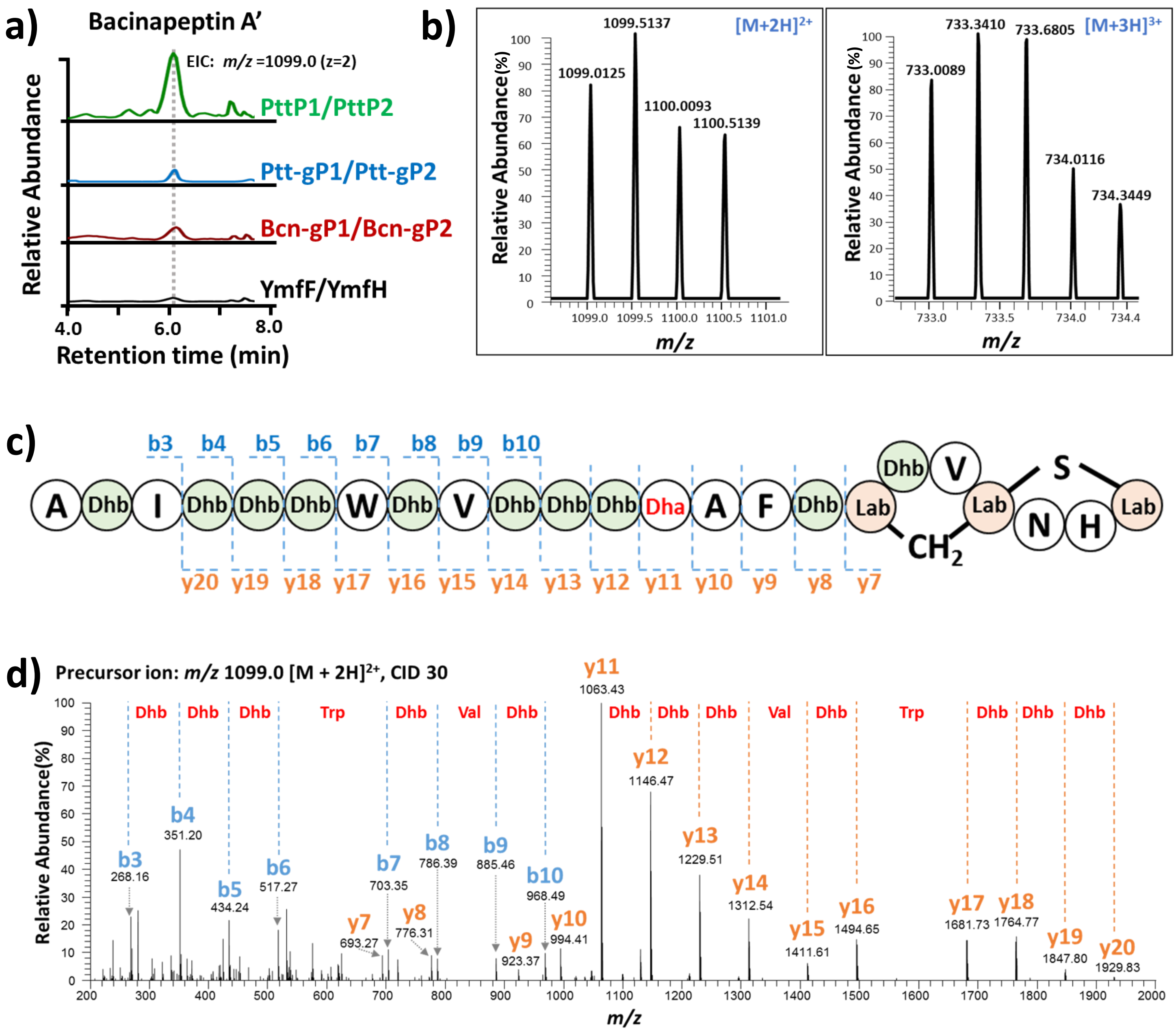

Proteolytic efficiency of Prot_819/Prot_176 proteases was compared using in vitro proteolysis of BcnKC-modified BcnA1. **a)** Extracted ion chromatograms (EICs) of m/z 1099.0 ($[M+2H]^{2+}$), representing the doubly charged state of bacinapeptin A′, are overlaid for comparison. In green, production of bacinapeptin A′ with PttP1/PttP2 proteases from the *ptt* BGC. In blue, Ptt-gP1/Ptt-gP2 exhibited proteolysis with less efficiency compared to PttP1/PttP2. In red, Bcn-gP1/Bcn-gP2 detected in *B. nakamurai* NRRL B-41092 demonstrated proteolytic activity towards BcnA1 with less efficiency than PttP1/PttP2. In black, YmfF/YmfH found in the *B. subtilis* 168 heterologous host, showed some proteolytic activity towards BcnA1. **b)** The high-resolution mass spectra of bacinapeptin A′ are included at right showing its doubly and triply charged states. **c)**.The structure of bacinapeptin A’. **d)** The doubly charged precursor ion, *m/z* 1099.0, was selected for collision induced dissociation (CID) at 30 eV. Major fragment ions are annotated with their b or y ion identity and the amino acid residues deduced from fragment ions are labelled in red.

Supplementary Figure 30: Phylogenetic tree of Prot_176 in *Paenibacillus*.

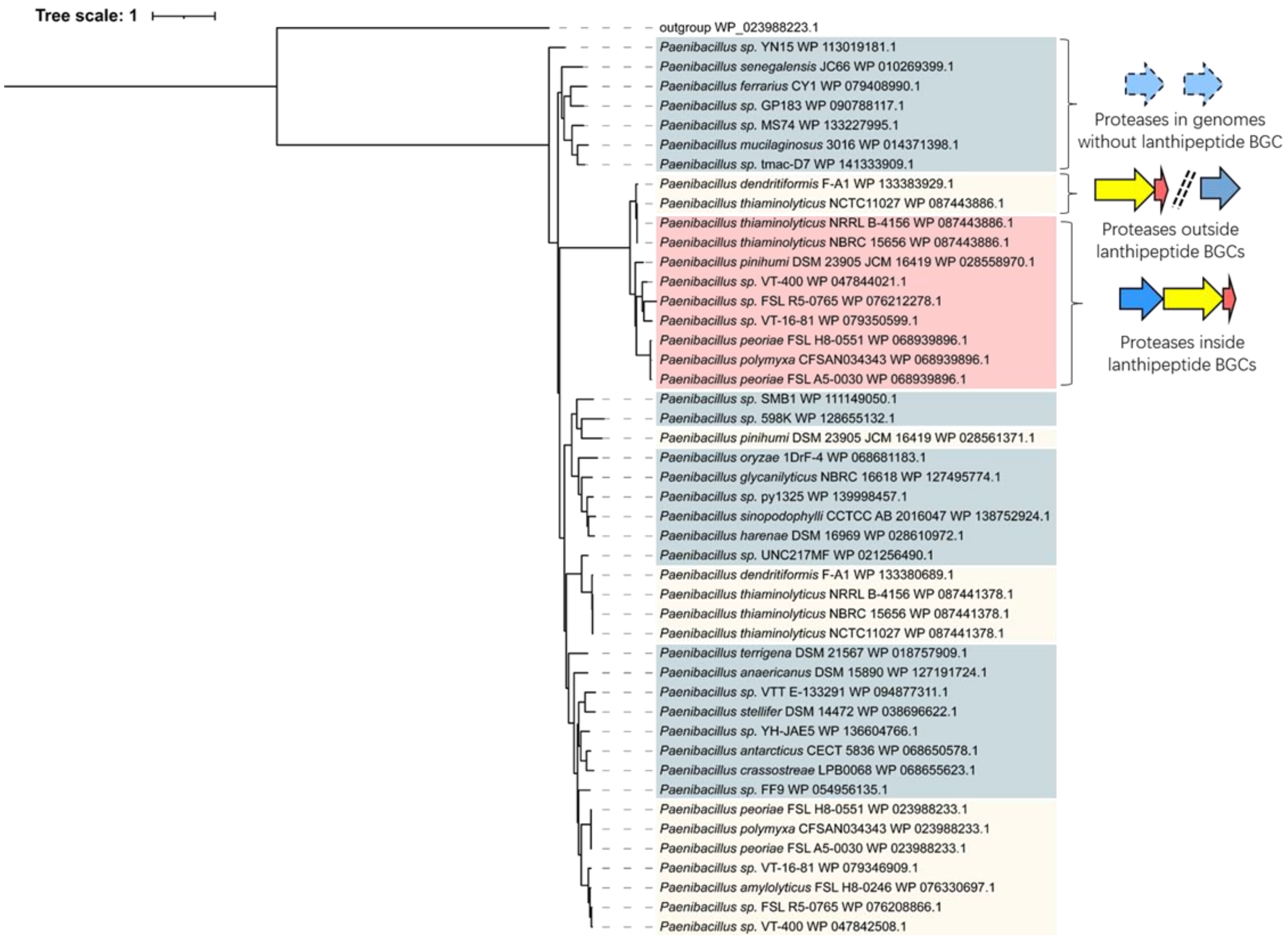

A phylogenetic tree was constructed using Prot_176 sequences in *Paenibacillus*. An outgroup WP_023988223.1, representing an M16B metallopeptidase found in *Paenibacillus* that does not belong to the Prot_176 group, was added and set as the root point for the tree. Proteases were categorized into three groups based on their location in their respective genome as indicated at right. Proteases in genomes without a lanthipeptide BGC are grouped in light blue, proteases in genomes harboring lanthipeptide BGCs but with the protease encoded outside of the lanthipeptide BGC are grouped in light yellow, and proteases encoded within lanthipeptide BGCs are grouped in red.

Supplementary Figure 31: In vitro activity of PttP1/PttP2 mutations.

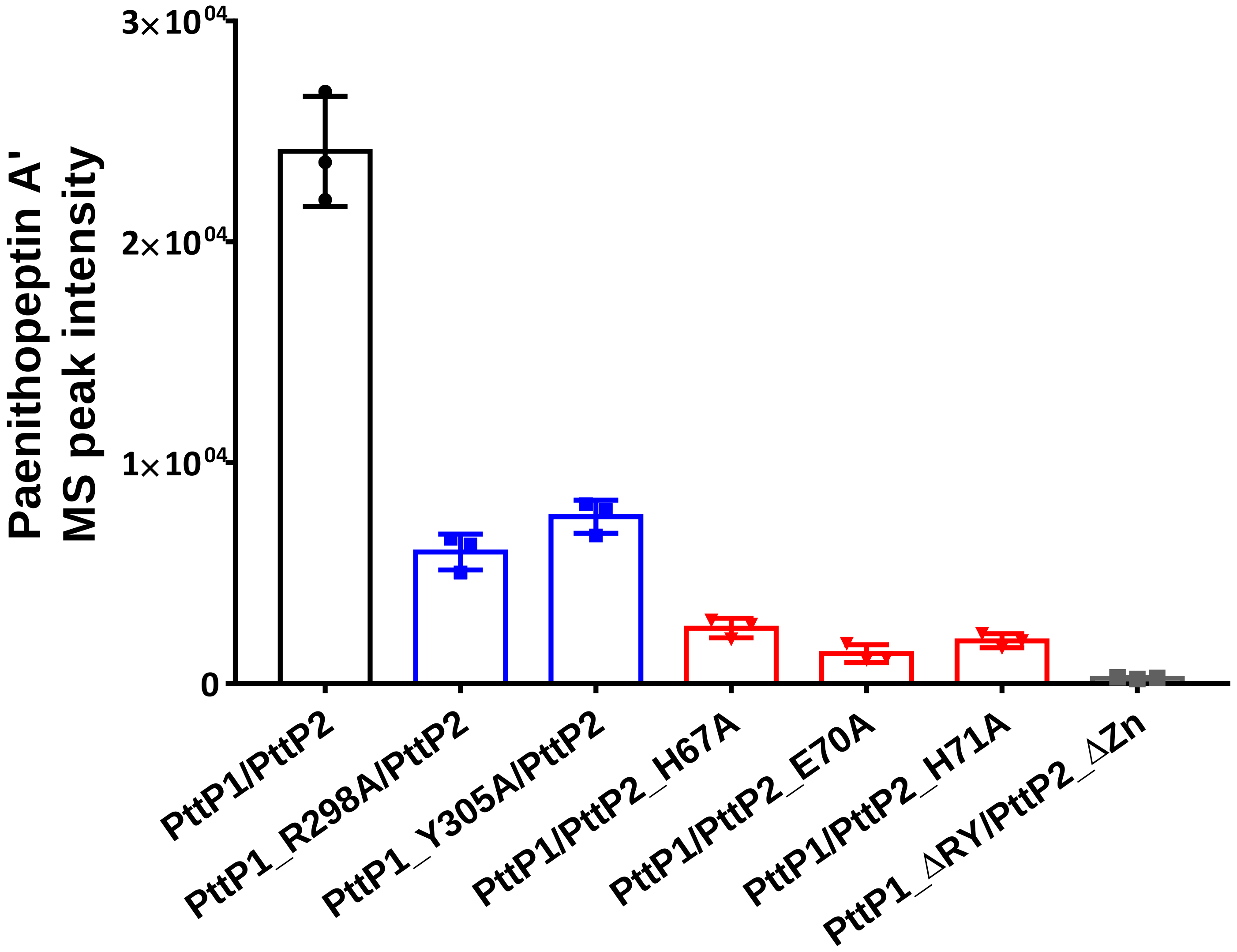

The H67, E70, and H71 of the HXXEH motif and R298 and Y305 of the R/Y pair were mutated individually to Ala, leading to decreased activity of PttP1/PttP2 in vitro for each mutation. Simultaneous mutation of all five residues to Ala completely abolished the production of paenithopeptin A′. The first column (black) indicates the production of paenithopeptin A′ using wild type PttP1/PttP2. The next two columns (blue) represent the production with varying mutations to the R/Y pair of PttP1. The next three columns (red) indicate the production with mutations to the HXXEH motif of PttP2. The last column (grey, obscured by relative height) represents lost production with mutations to both conserved motifs of PttP1 and PttP2. Production of paenithopeptins B′-E′ showed the same trend. Error bars indicate standard deviation across triplicates. Data shown as mean ± SD (each group n = 3; SD: standard deviation).

Supplementary Figure 32: PttP1/PttP2 activity is metal dependent

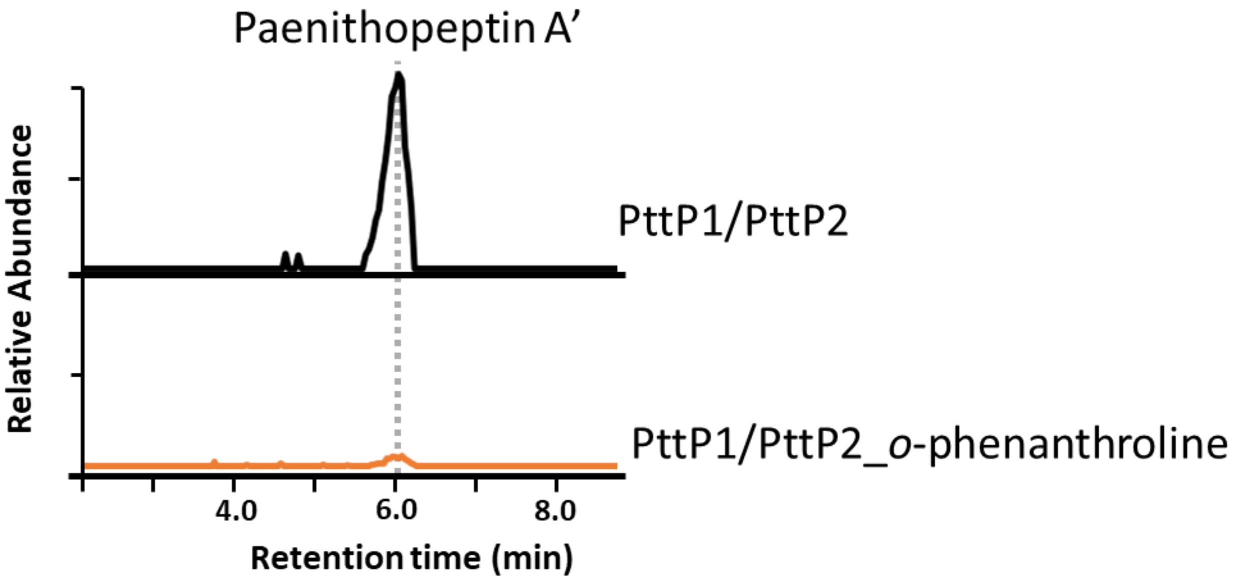

Addition of the metal-chelating compound, *o*-phenanthroline, to an in vitro assay containing PttKC modified PttA1 resulted in significant inhibition of the proteolytic activity of PttP1/PttP2. Extracted ion chromatograms (EIC) of m/z 949.5 ($[M+2H]^{2+}$), representing the doubly charged state of paenithopeptin A′, are overlaid for comparison. In black, PttP1/PttP2 processes PttKC modified PttA1 to produce paenithopeptin A′. In orange, PttP2 was incubated with *o*-phenanthroline prior to addition to the in vitro assay, significantly reducing the production of paenithopeptin A′.

Supplementary Figure 33: PttP1/PttP2 are responsible for the processing of paenithopeptin A2

a)

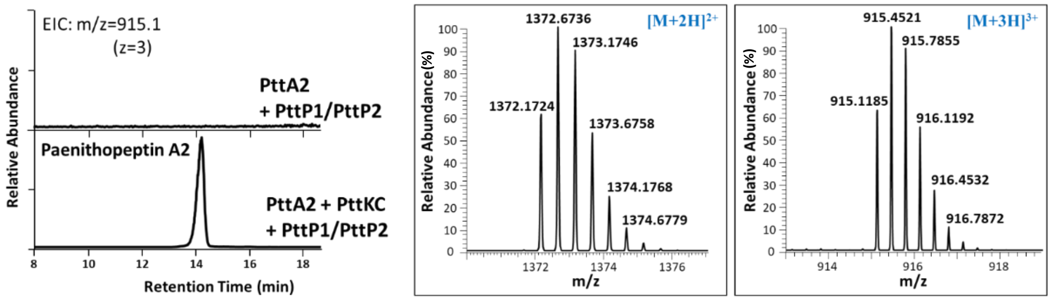

b)

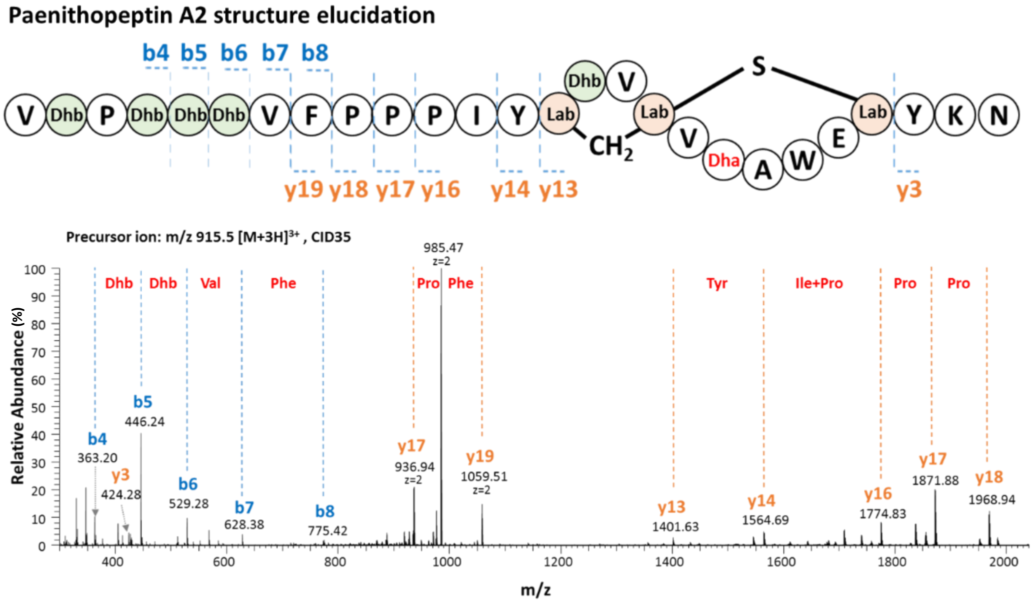

c)

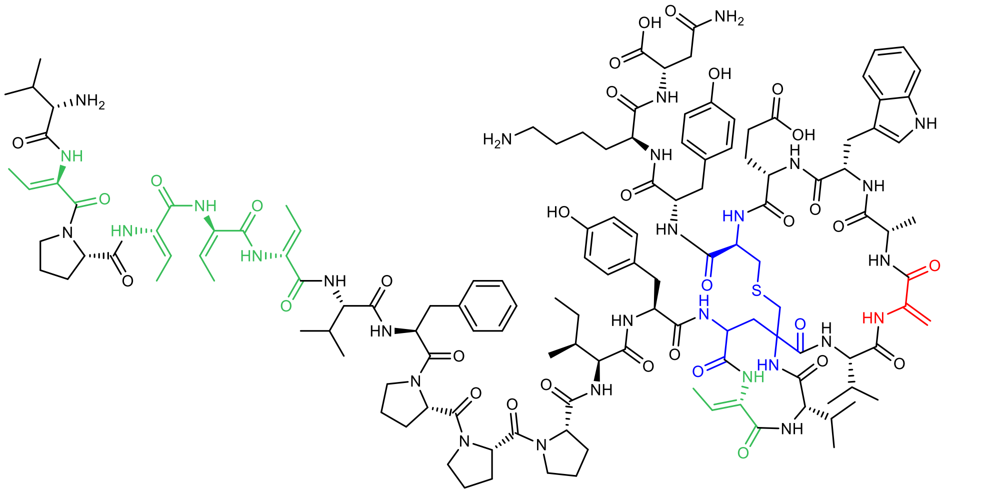

In vitro enzymatic assays revealed that PttP1/PttP2 could also cleave precursor peptide PttA2 which contains the conserved Q-A-(A/I)-(D/E) motif in the leader peptide sequence. **a)** An extracted ion chromatogram (EIC) of m/z 915.1 representing the triply charged state ($[M+3H]^{3+}$) is overlaid against a control in vitro assay for comparison of paenithopepin A2 production. At right, doubly and triply charged states are presented for paenithopeptin A2. **b)** The structure of paenithopeptin A2 is presented with fragmentation points of corresponding b and y ions as well as the location of the labionin ring marked. MS/MS was used to confirm the amino acid sequence by analysis of the fragmentation patterns. The precursor ion m/z 915.5 representing $[M+3H]^{3+}$ was used for collision induced dissociation (CID) at 35 eV. Major fragment ions are annotated with their b or y ion identity and the amino acid residues deduced from fragment ions are labelled in red. **c)** Chemical structure of paenithopeptin A2. Chemical formula: $C_{134}H_{183}N_{29}O_{32}S$. Dhb is in green. Dha is in red. Ser and Cys involved in the labionin ring are in blue.

Supplementary Figure 34: PttP1/PttP2 are responsible for the processing of paenithopeptin A3

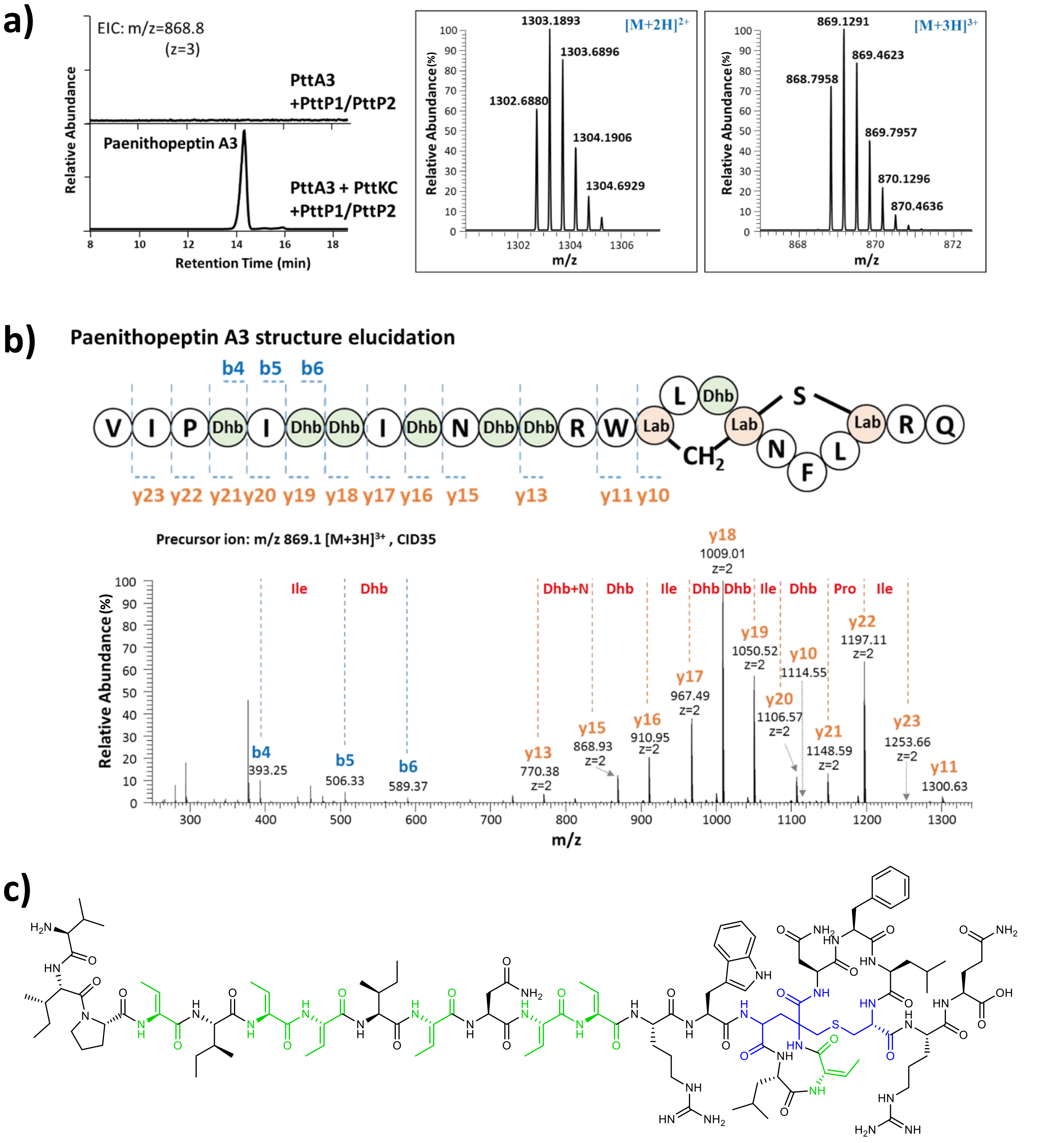

a) An extracted ion chromatogram (EIC) of m/z 868.8 representing the triply charged state ($[M+3H]^{3+}$) is overlaid against a control in vitro assay for comparison of paenithopeptin A3 production. At right, doubly and triply charged states are presented for paenithopeptin A3. **b)** The structure of paenithopeptin A3 is presented with fragmentation points of corresponding b and y ions as well as the location of the labionin ring marked. MS/MS was used to confirm the amino acid sequence by analysis of the fragmentation patterns. The precursor ion m/z 869.1 representing $[M+3H]^{3+}$ was used for collision induced dissociation (CID) at 35 eV. Major fragment ions are annotated with their b or y ion identity and the amino acid residues deduced from fragment ions are labelled in red. **c)** Chemical structure of paenithopeptin A3. Chemical formula: $C_{122}H_{182}N_{34}O_{28}S$. Dhb is in green. Ser and Cys involved in the labionin ring are in blue.

Supplementary Figure 35: PttP1/PttP2 are responsible for the processing of paenithopeptin A5

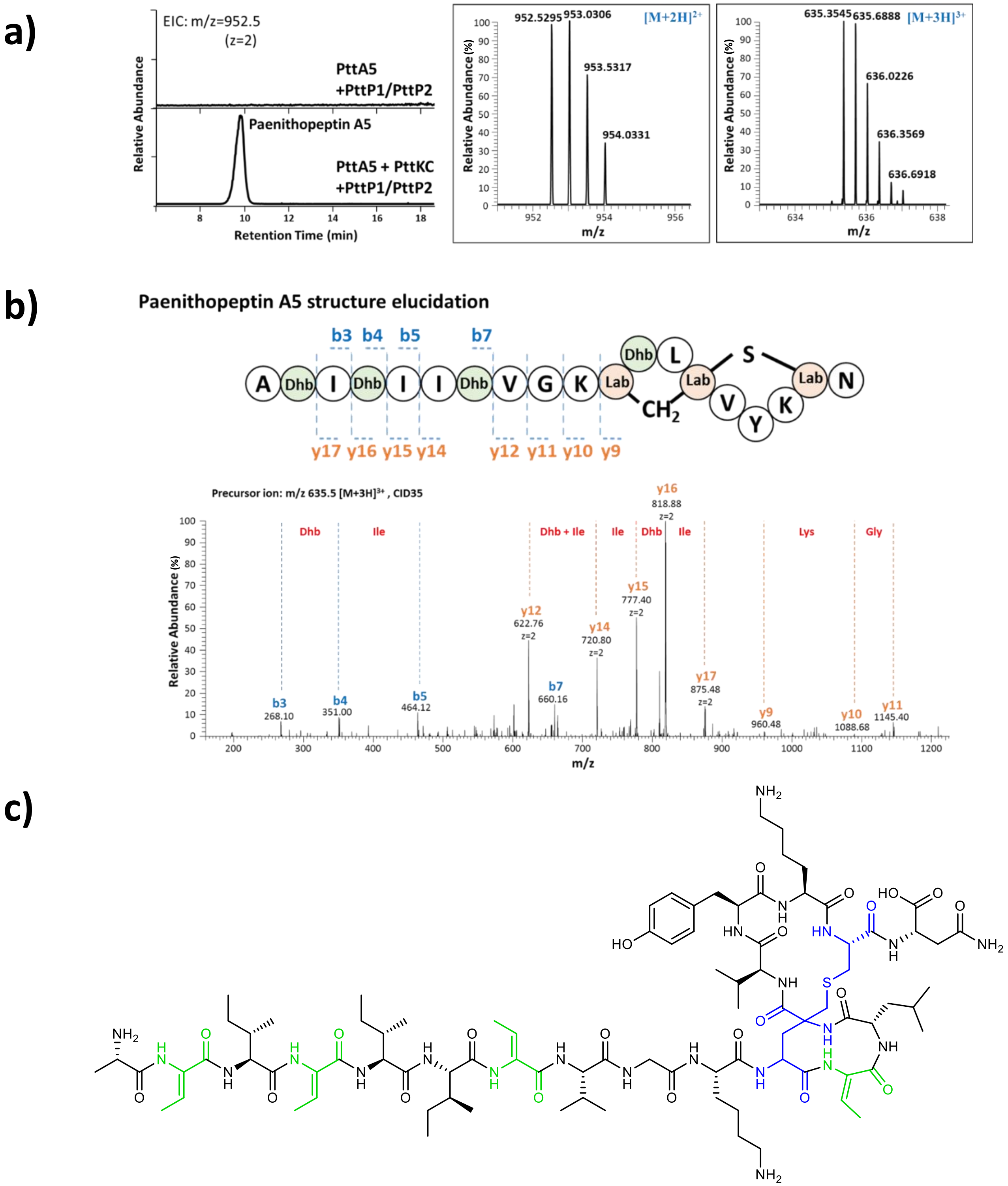

a) An extracted ion chromatogram (EIC) of m/z 952.5 representing the doubly charged state ($[M+2H]^{2+}$) is overlaid against a control in vitro assay for comparison of paenithopeptin A5 production. At right, doubly and triply charged states are presented for paenithopeptin A5. **b)** The structure of paenithopeptin A5 is presented with fragmentation points of corresponding b and y ions as well as the location of the labionin ring marked. MS/MS was used to confirm the amino acid sequence by analysis of the fragmentation patterns. The precursor ion m/z 635.5 representing the triply charged state ($[M+3H]^{3+}$) was used for collision induced dissociation (CID) at 35 eV. Major fragment ions are annotated with their b or y ion identity and the amino acid residues deduced from fragment ions are labelled in red. **c)** Chemical structure of paenithopeptin A5. Chemical formula: $C_{89}H_{142}N_{22}O_{22}S$. Dhb is in green. Ser and Cys involved in the labionin ring are in blue.

Supplementary Figure 36: PttP1/PttP2 are responsible for the processing of paenithopeptin A7

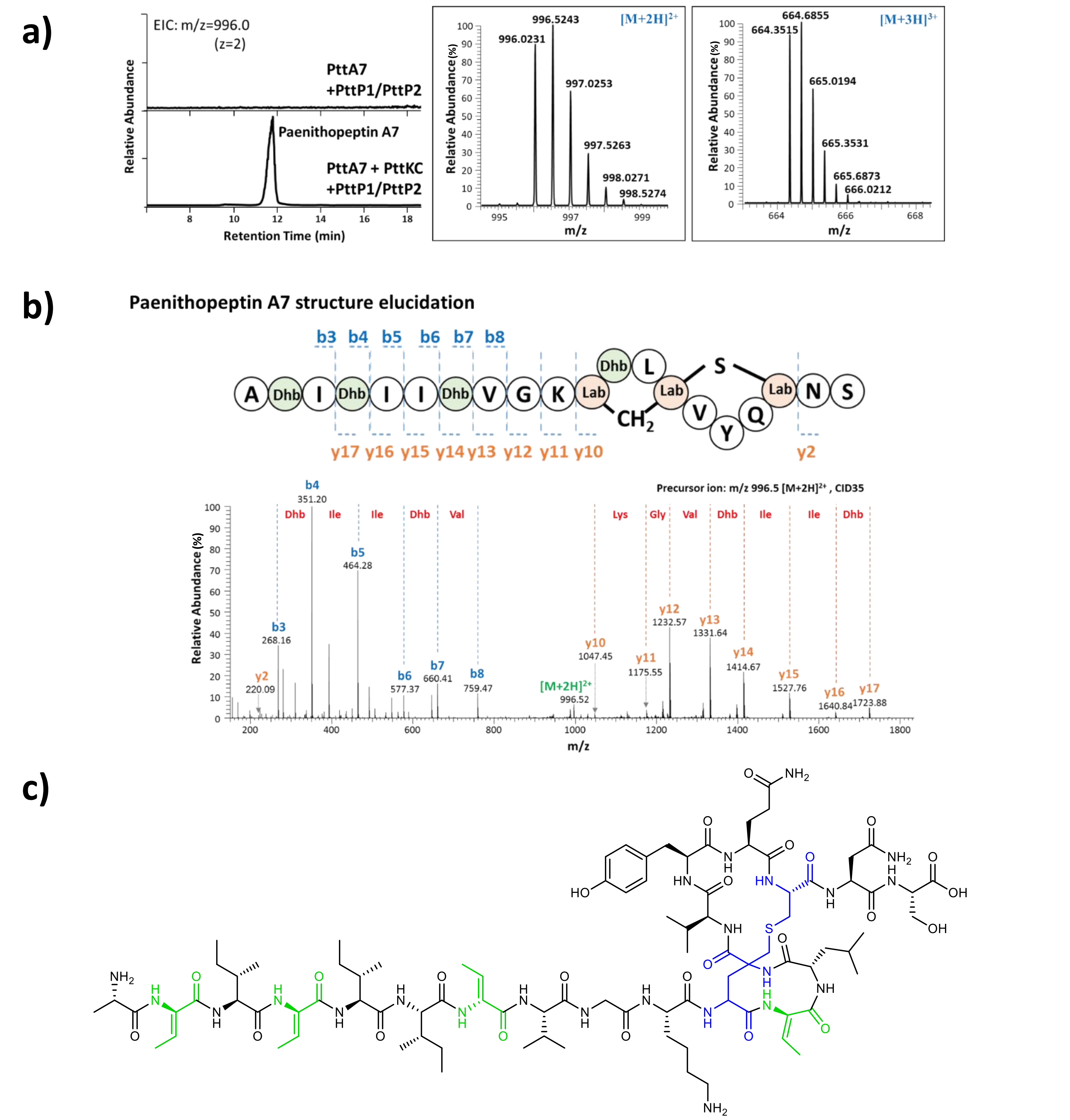

a) An extracted ion chromatogram (EIC) of m/z 996.0 representing the doubly charged state ($[M+2H]^{2+}$) is overlaid against a control in vitro assay for comparison of paenithopeptin A7 production. At right, doubly and triply charged states are presented for paenithopeptin A7. **b)** The structure of paenithopeptin A7 is presented with fragmentation points of corresponding b and y ions as well as the location of the labionin ring marked. MS/MS was used to confirm the amino acid sequence by analysis of the fragmentation patterns. The precursor ion m/z 996.5 representing $[M+2H]^{2+}$ was used for collision induced dissociation (CID) at 35 eV. Major fragment ions are annotated with their b or y ion identity and the amino acid residues deduced from fragment ions are labelled in red. **c)** Chemical structure of paenithopeptin A7. Chemical formula: $C_{91}H_{143}N_{23}O_{25}S$. Dhb is in green. Ser and Cys involved in the labionin ring are in blue.

Supplementary Figure 37: In vitro production and LC-MS analysis of paenithopeptin A2 and analogs by PttP1/PttP2

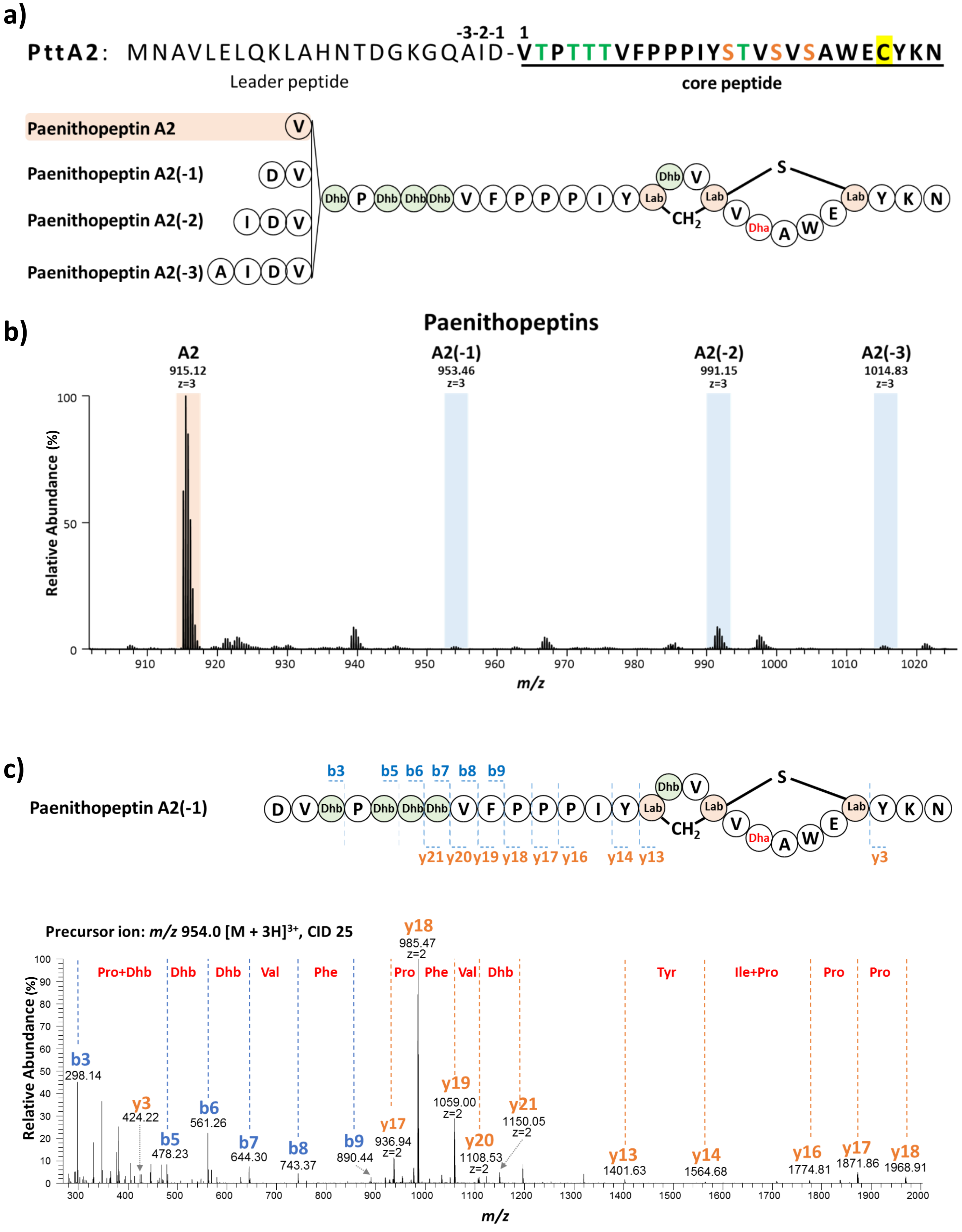

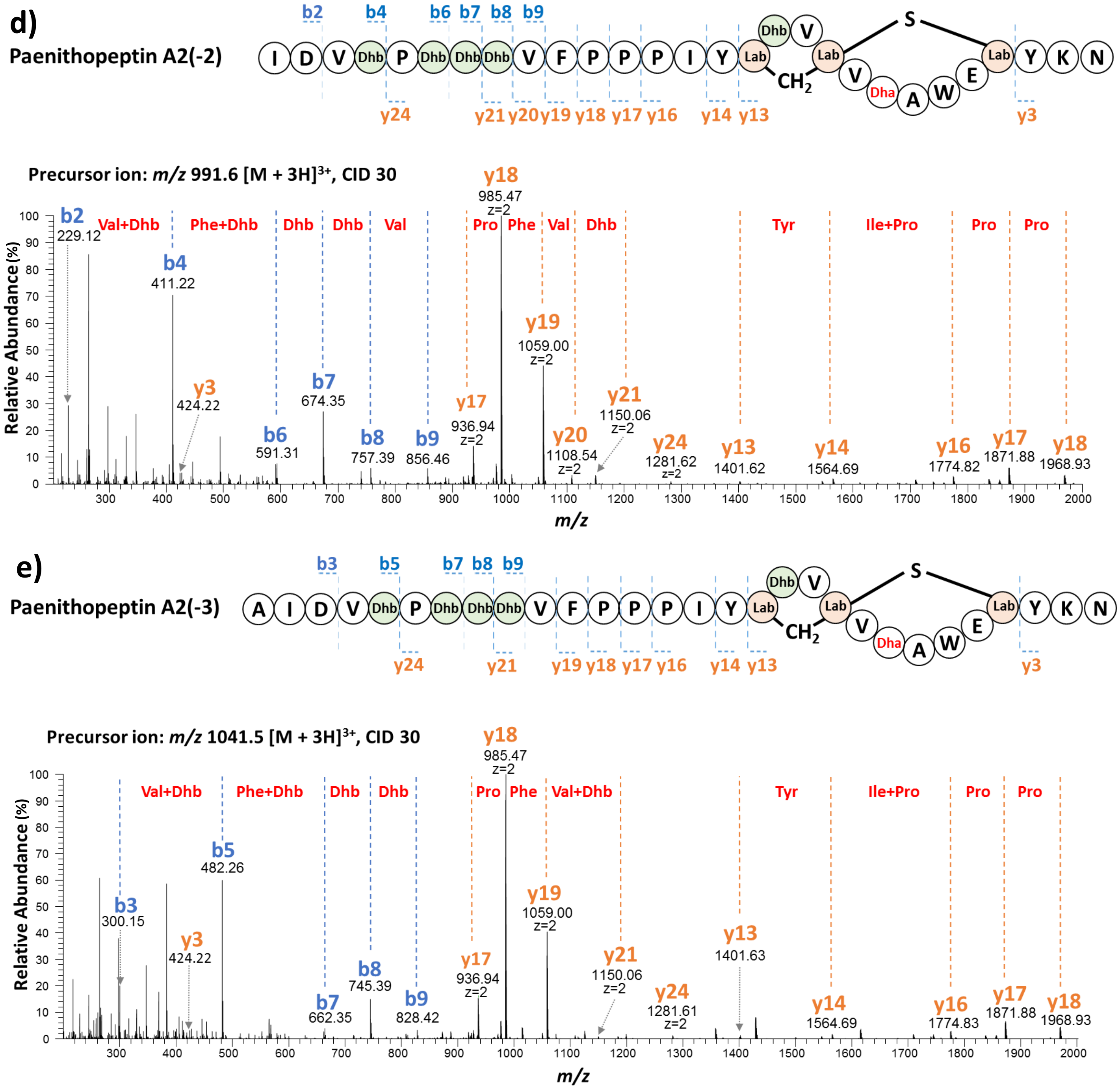

PttA2 produced lanthipeptides with different amino acids overhangs by PttP1/PttP2 in vitro. **a)** The structure of PttA2 with the leader peptide and core peptide regions indicated and separated by a hyphen. The aminopeptidase activity of the PttP1/PttP2 resulted in the production of a series of compounds differing by one amino acid residue as represented by paenithopeptins A2 – A2(-3). Increasing numbers of additional residues are indicated with increasing x in (-x) denoting the distance from the cleavage site of the main product. **b)** High resolution mass spectrometry was used to analyze the resulting lanthipeptide products with triply charged states highlighted in the spectrum. Peaks are labeled corresponding to the structures presented in **(a)**. **c) - e)** The structures of paenithopeptin A2(-1) **(c)**, paenithopeptin A2(-2) **(d)**, and paenithopeptin A2(-3) **(e)** are presented with fragmentation points of corresponding b and y ions as well as the location of the labionin ring marked. MS/MS was used to confirm the amino acid sequence by analysis of the fragmentation patterns. Major fragment ions are annotated with their b or y ion identity, and the amino acid residues deduced from fragment ions are labelled in red.

Supplementary Figure 38: In vitro production and LC-MS analysis of paenithopeptin A3 and analogs by PttP1/PttP2

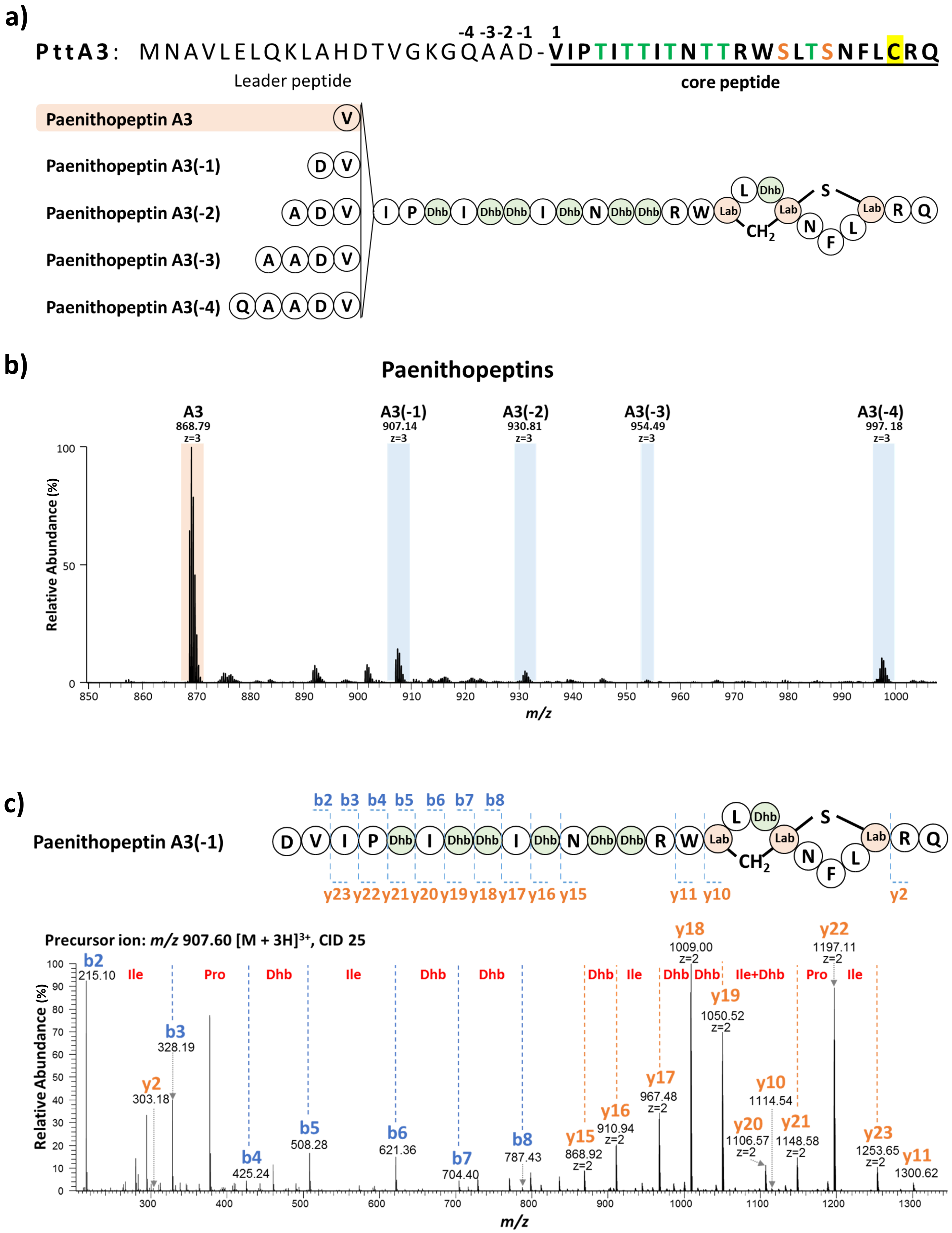

d)

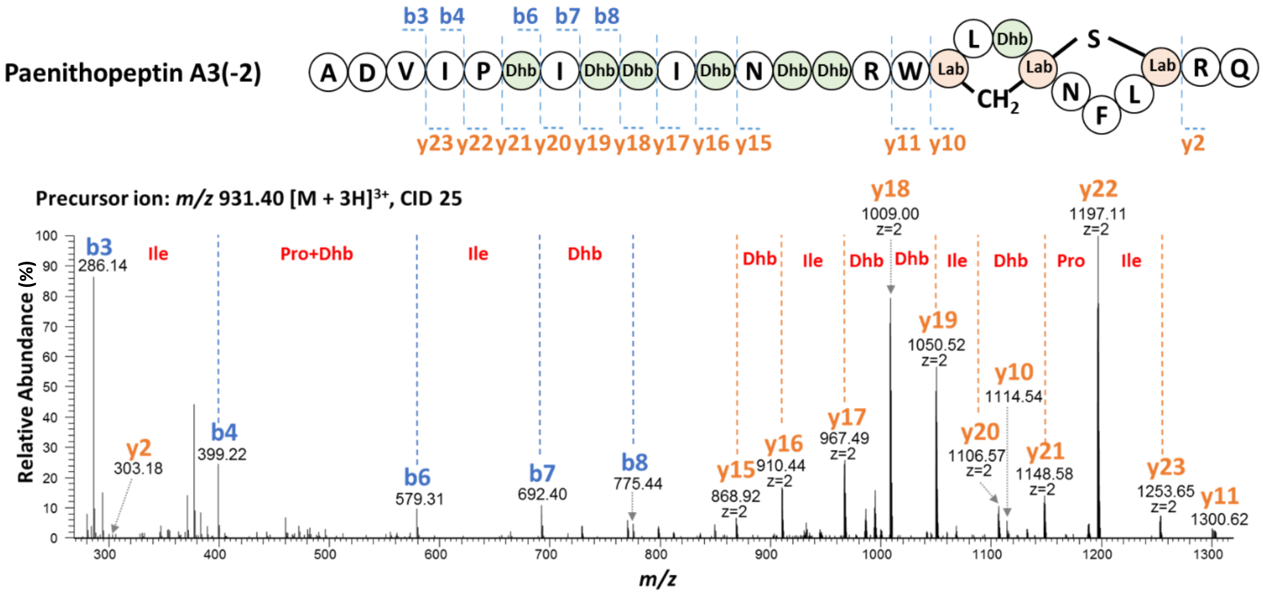
Paenithopeptin A3(-2)
A D V I P Dhb I Dhb Dhb I Dhb N Dhb Dhb R W Lab L Dhb Lab S Lab R Q N F L CH2
b3 b4 b6 b7 b8
y23 y22 y21 y20 y19 y18 y17 y16 y15 y11 y10 y2
Precursor ion: m/z 931.40 [M + 3H]3+, CID 25
Relative Abundance (%)
m/z
Ile Pro+Dhb Ile Dhb Dhb Ile Dhb Dhb Ile Dhb Pro Ile
b3 286.14
y2 303.18
b4 399.22
b6 579.31
b7 692.40
b8 775.44
y15 868.92 z=2
y16 910.44 z=2
y17 967.49 z=2
y18 1009.00 z=2
y19 1050.52 z=2
y20 1106.57 z=2
y10 1114.54
y21 1148.58 z=2
y22 1197.11 z=2
y23 1253.65 z=2
y11 1300.62

e)

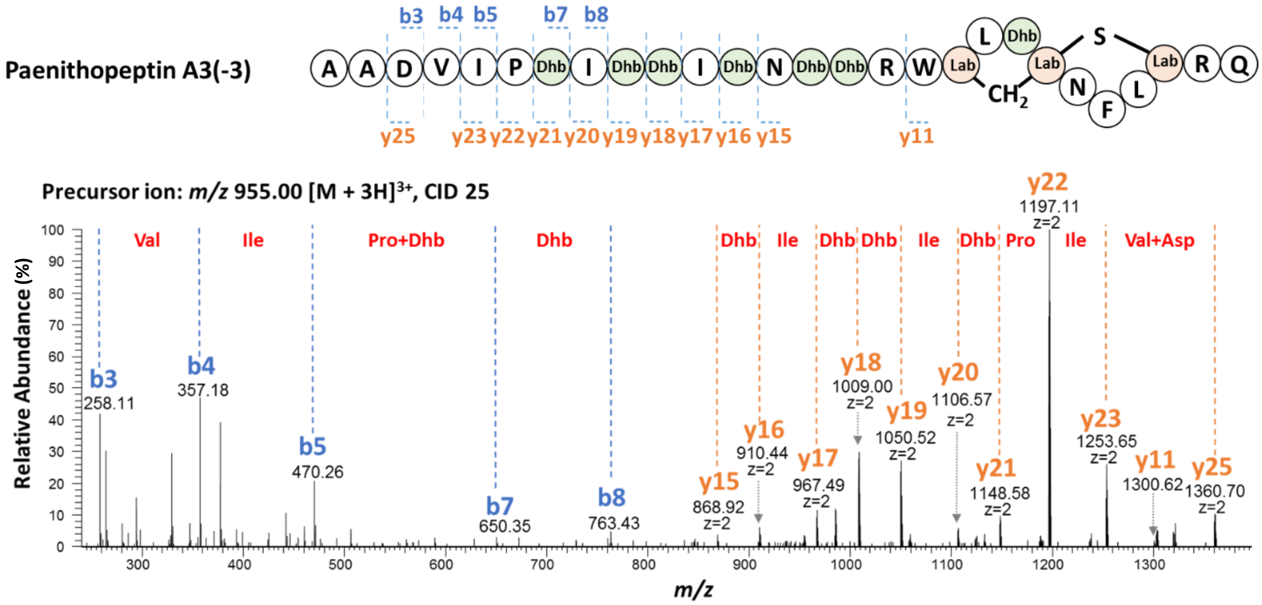
Paenithopeptin A3(-3)
A A D V I P Dhb I Dhb Dhb I Dhb N Dhb Dhb R W Lab L Dhb Lab S Lab R Q N F L CH2
b3 b4 b5 b7 b8
y25 y23 y22 y21 y20 y19 y18 y17 y16 y15 y11
Precursor ion: m/z 955.00 [M + 3H]3+, CID 25
Relative Abundance (%)
m/z
Val Ile Pro+Dhb Dhb Dhb Ile Dhb Dhb Ile Dhb Pro Ile Val+Asp
b3 258.11
b4 357.18
b5 470.26
b7 650.35
b8 763.43
y15 868.92 z=2
y16 910.44 z=2
y17 967.49 z=2
y18 1009.00 z=2
y19 1050.52 z=2
y20 1106.57 z=2
y21 1148.58 z=2
y22 1197.11 z=2
y23 1253.65 z=2
y11 1300.62
y25 1360.70 z=2

f)

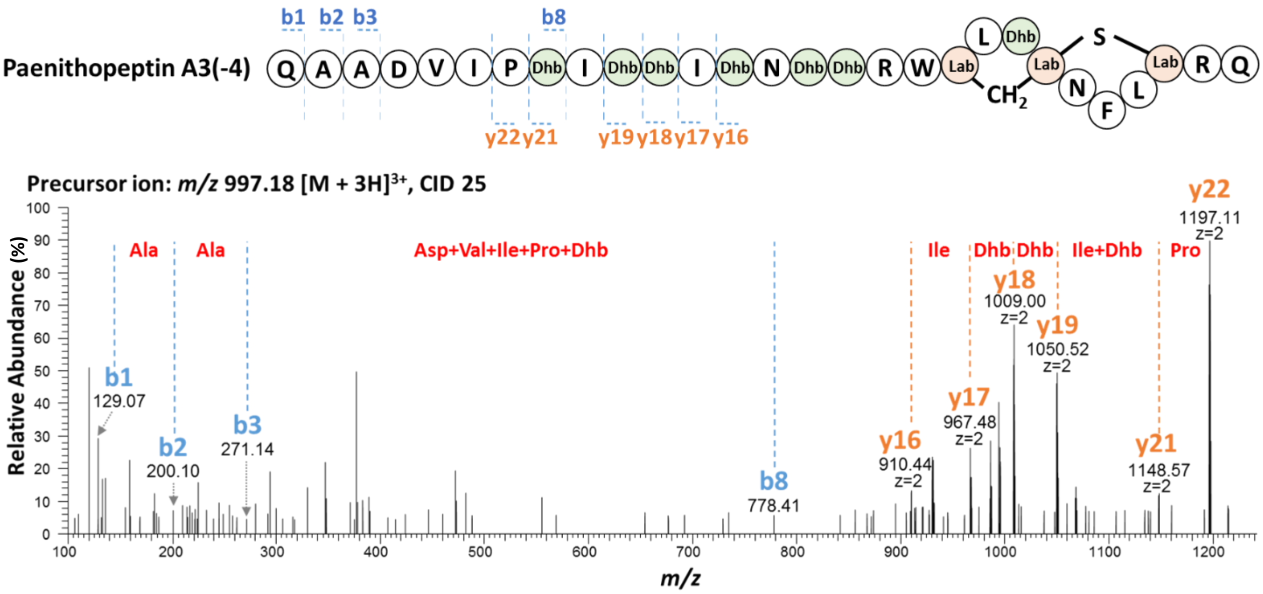
Paenithopeptin A3(-4)
Q A A D V I P Dhb I Dhb Dhb I Dhb N Dhb Dhb R W Lab L Dhb Lab S Lab R Q N F L CH2
b1 b2 b3 b8
y22 y21 y19 y18 y17 y16
Precursor ion: m/z 997.18 [M + 3H]3+, CID 25
Relative Abundance (%)
m/z
Ala Ala Asp+Val+Ile+Pro+Dhb Ile Dhb Dhb Ile+Dhb Pro
b1 129.07
b2 200.10
b3 271.14
b8 778.41
y16 910.44 z=2
y17 967.48 z=2
y18 1009.00 z=2
y19 1050.52 z=2
y21 1148.57 z=2
y22 1197.11 z=2

PttA3 produced lanthipeptides with different amino acids overhangs by PttP1/PttP2 in vitro. **a)** The structure of PttA3 with the leader peptide and core peptide regions indicated and separated by a hyphen. The aminopeptidase activity of the PttP1/PttP2 should result in the production of a series of compounds differing by one amino acid residue as represented by paenithopeptins A3 – A3(-4). Increasing numbers of additional residues are indicated with increasing x in (-x) denoting the distance from the cleavage site of the main product. **b)** High resolution mass spectrometry was used to analyze the resulting lanthipeptide products with triply charged states highlighted in the spectrum. Peaks are labeled corresponding to the structures presented in **(a)**. **c) - f)** The structures of paenithopeptin A3(-1) **(c)**, paenithopeptin A3(-2) **(d)**, paenithopeptin A3(-3) **(e)**, and paenithopeptin A3(-4) **(f)** are presented with fragmentation points of corresponding b and y ions as well as the location of the labionin ring marked. MS/MS was used to confirm the amino acid sequence by analysis of the fragmentation patterns. Major fragment ions are annotated with their b or y ion identity, and the amino acid residues deduced from fragment ions are labelled in red.

Supplementary Figure 39: In vitro production and LC-MS analysis of paenithopeptin A5 and analogs by PttP1/PttP2

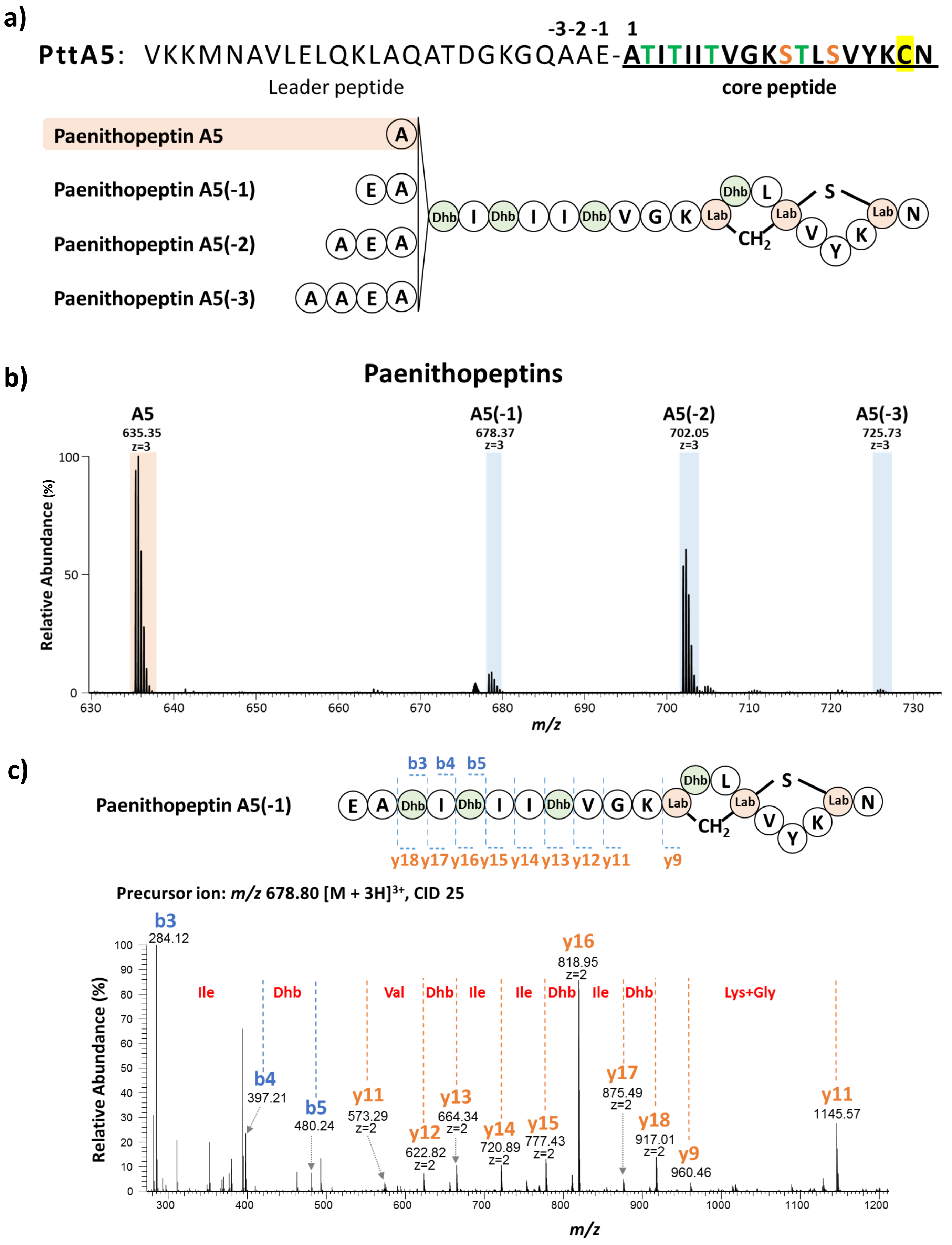

d)

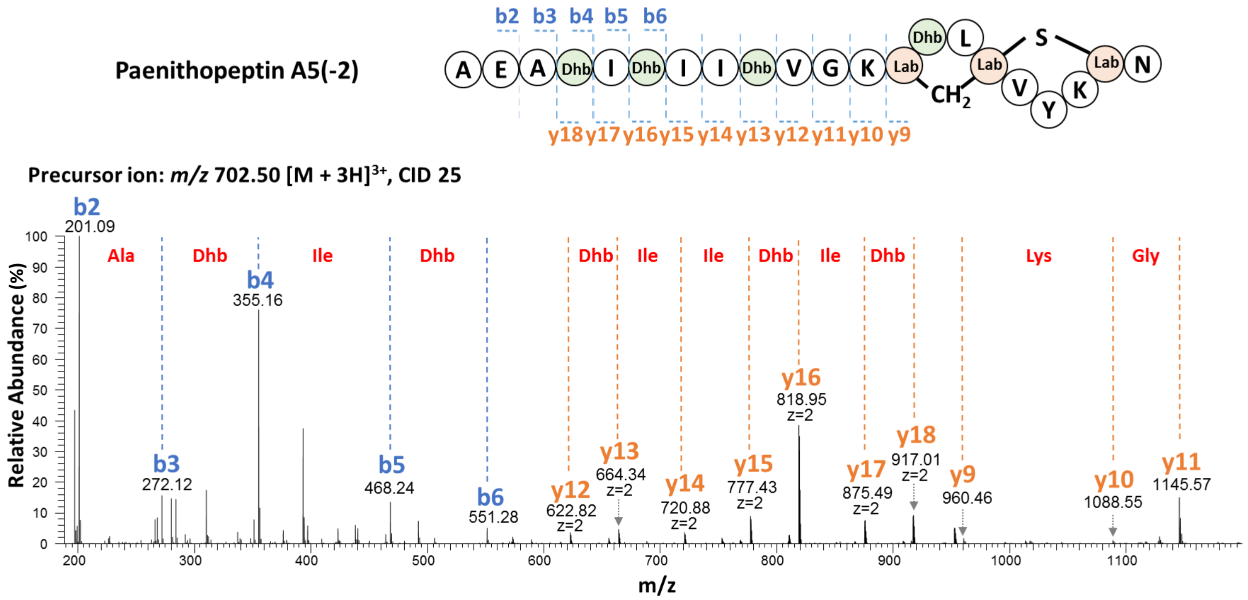

e)

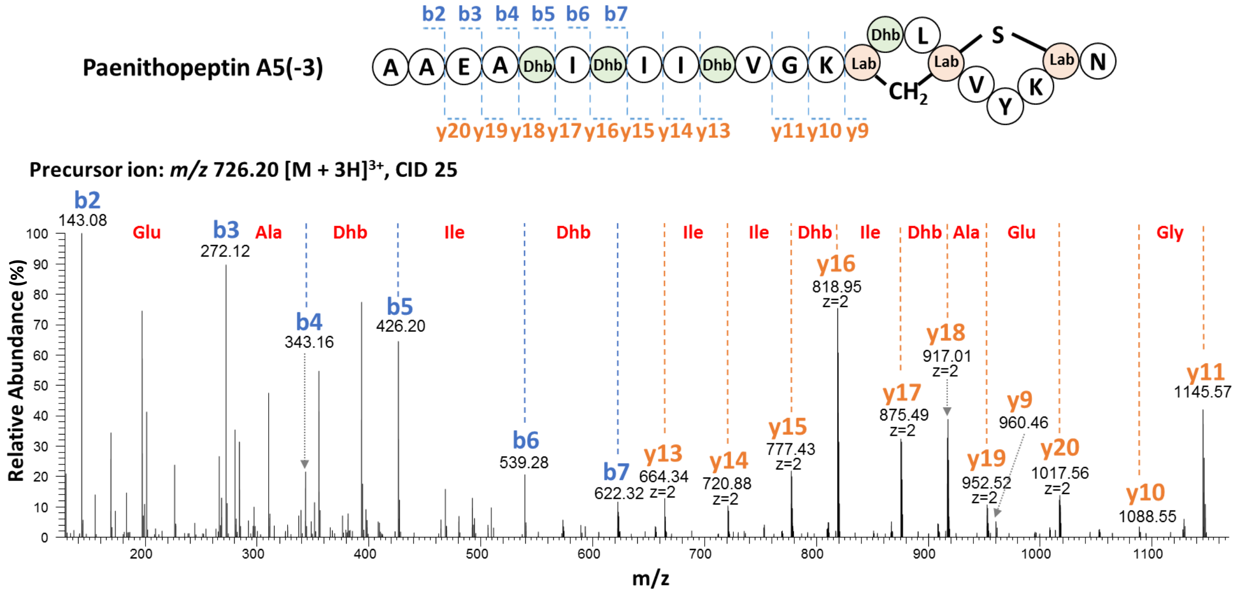

PttA5 produced lanthipeptides with different amino acids overhangs by PttP1/PttP2 in vitro. **a)** The structure of PttA5 with the leader peptide and core peptide regions indicated and separated by a hyphen. The aminopeptidase activity of the PttP1/PttP2 should result in the production of a series of compounds differing by one amino acid residue as represented by paenithopeptins A5 – A5(-3). Increasing numbers of additional residues are indicated with increasing x in (-x) denoting the distance from the cleavage site of the main product. **b)** High resolution mass spectrometry was used to analyze the resulting lanthipeptide products with triply charged states highlighted in the spectrum. Peaks are labeled corresponding to the structures presented in **(a)**. **c) - e)** The structures of paenithopeptin A5(-1) **(c)**, paenithopeptin A5(-2) **(d)**, and paenithopeptin A5(-3) **(e)** are presented with fragmentation points of corresponding b and y ions as well as the location of the labionin ring marked. MS/MS was used to confirm the amino acid sequence by analysis of the fragmentation patterns. Major fragment ions are annotated with their b or y ion identity, and the amino acid residues deduced from fragment ions are labelled in red.

Supplementary Figure 40: In vitro production and LC-MS analysis of paenithopeptin A7 and analogs by PttP1/PttP2

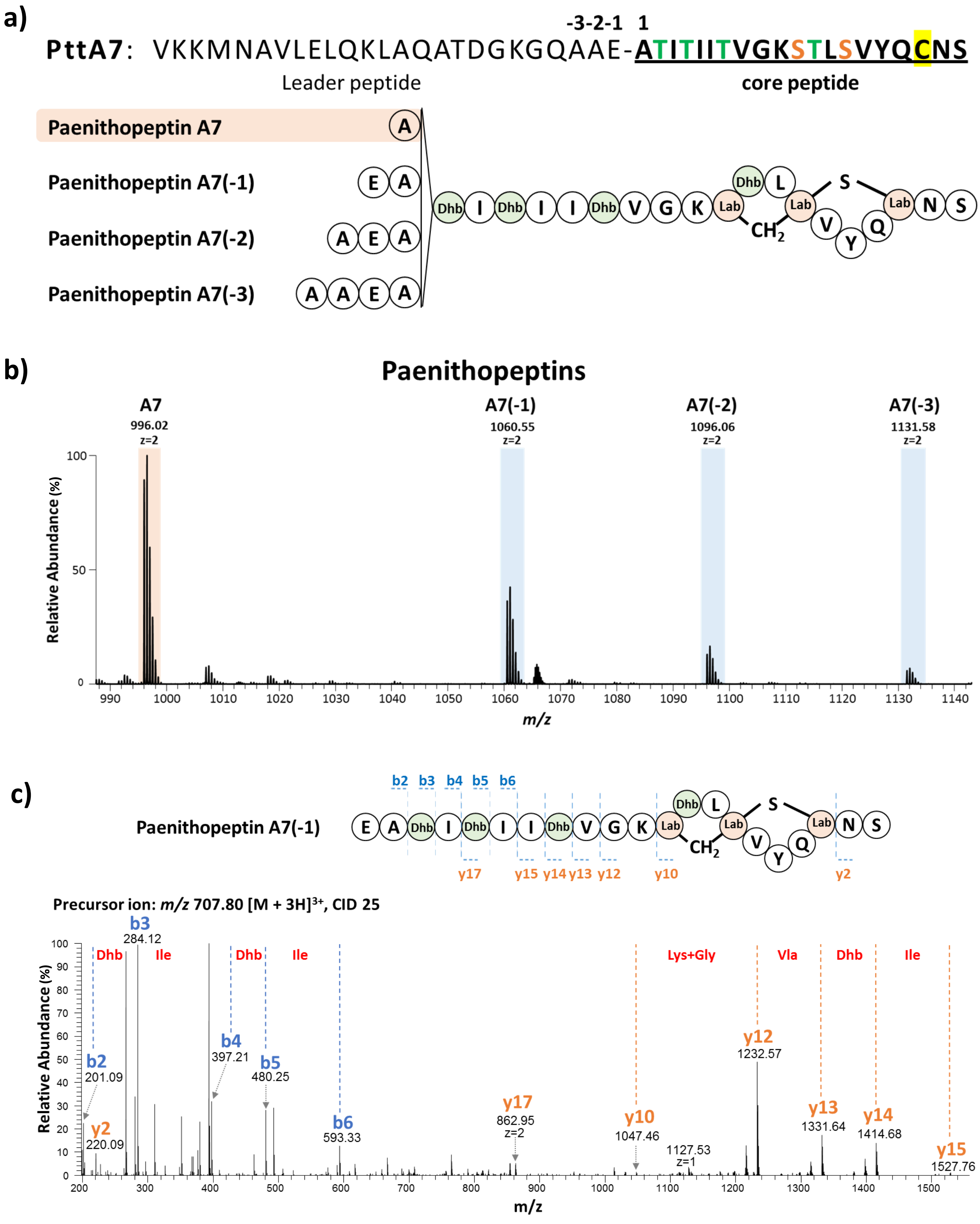

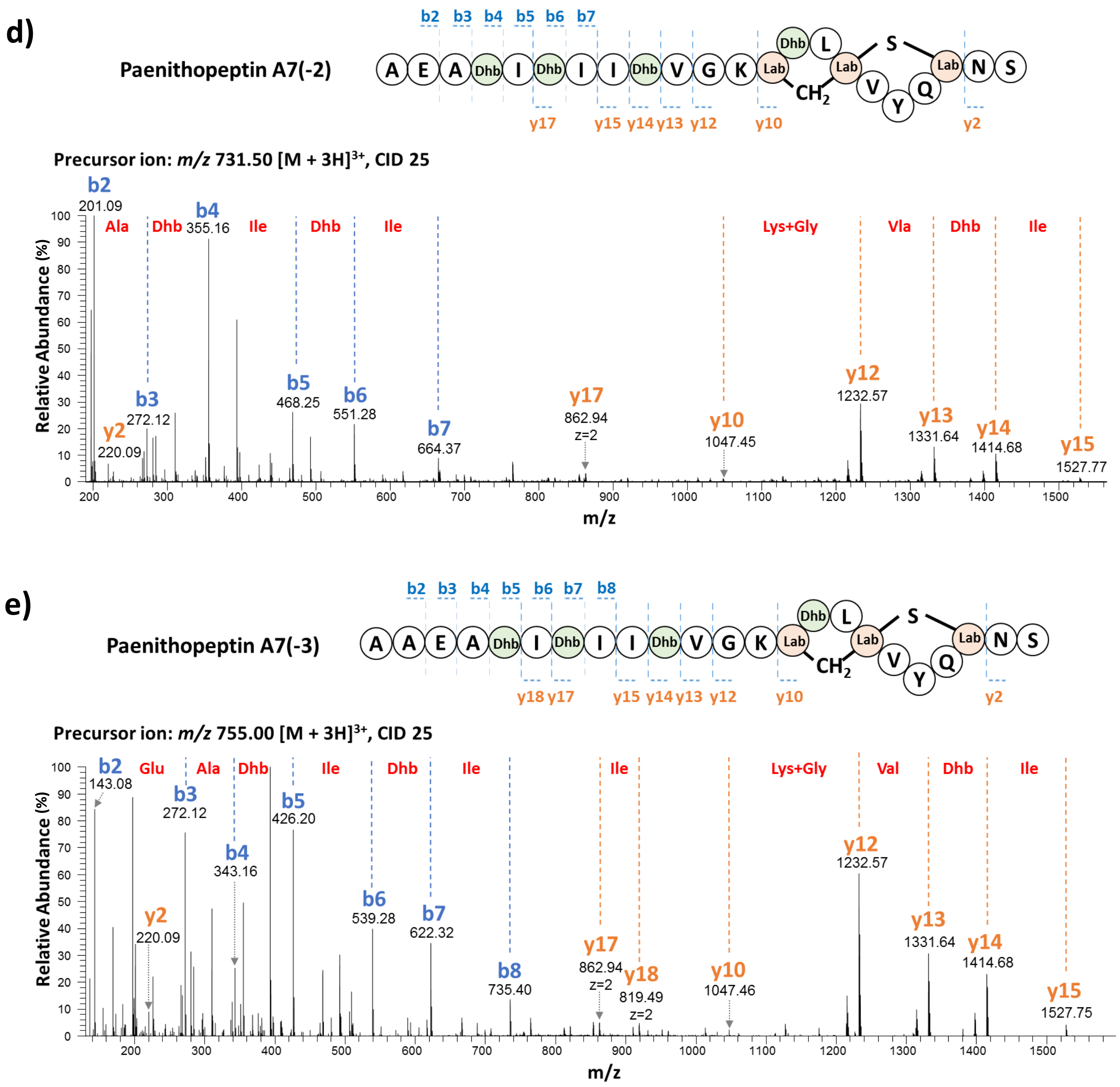

PttA7 produced lanthipeptides with different amino acids overhangs by PttP1/PttP2 in vitro. **a)** The structure of PttA7 with the leader peptide and core peptide regions indicated and separated by a hyphen. The aminopeptidase activity of the PttP1/PttP2 should result in the production of a series of compounds differing by one amino acid residue as represented by paenithopeptins A7 – A7(-3). Increasing numbers of additional residues are indicated with increasing x in (-x) denoting the distance from the cleavage site of the main product. **b)** High resolution mass spectrometry was used to analyze the resulting lanthipeptide products with triply charged states highlighted in the spectrum. Peaks are labeled corresponding to the structures presented in **(a)**. **c) - e)** The structures of paenithopeptin A7(-1) **(c)**, paenithopeptin A7(-2) **(d)**, and paenithopeptin A7(-3) **(e)** are presented with fragmentation points of corresponding b and y ions as well as the location of the labionin ring marked. MS/MS was used to confirm the amino acid sequence by analysis of the fragmentation patterns. Major fragment ions are annotated with their b or y ion identity, and the amino acid residues deduced from fragment ions are labelled in red.

Supplementary Tables

Supplementary Table 1

The 120 unique Pfam domains of our library of 44,260 prospective lanthipeptide proteases used to search for cryptic proteases. The first three lines of the table represent the 6 Pfam domains contained by the known lanthipeptide proteases LanP (PF00082), LanT (PF03412), FlaP (PF02897 and PF00326), and AplP (PF01433 and PF17900).

Pfam	Description	Pfam	Description
PF00082	Subtilase family	PF03412	Peptidase C39 family
PF02897	Prolyl oligopeptidase, N-terminal beta-propeller domain	PF00326	Prolyl oligopeptidase family
PF01433	Peptidase family M1 domain	PF17900	Peptidase M1 N-terminal domain
PF18818	Zincin-like metallopeptidase	PF03413	Peptidase propeptide and YPEB domain
PF00089	Trypsin	PF03572	Peptidase family S41
PF00112	Papain family cysteine protease	PF03575	Peptidase family S51
PF00144	Beta-lactamase	PF03576	Peptidase family S58
PF00246	Zinc carboxypeptidase	PF03577	Peptidase family C69
PF02983	Alpha-lytic protease prodomain	PF03734	L,D-transpeptidase catalytic domain
PF00413	Matrixin	PF03959	Serine hydrolase (FSH1)
PF00557	Metallopeptidase family M24	PF04151	Bacterial pre-peptidase C-terminal domain
PF00561	alpha/beta hydrolase fold	PF04307	LexA-binding, inner membrane-associated putative hydrolase
PF00574	Clp protease	PF04327	Cysteine protease Prp
PF00675	Insulinase (Peptidase family M16)	PF04389	Peptidase family M28
PF00753	Metallo-beta-lactamase superfamily	PF04909	Amidohydrolase
PF00756	Putative esterase	PF05170	AsmA family
PF00768	D-alanyl-D-alanine carboxypeptidase	PF05193	Peptidase M16 inactive domain
PF00795	Carbon-nitrogen hydrolase	PF05195	Aminopeptidase P, N-terminal domain
PF00883	Cytosol aminopeptidase family, catalytic domain	PF05343	M42 glutamyl aminopeptidase
PF00905	Penicillin binding protein transpeptidase domain	PF05362	Lon protease (S16) C-terminal proteolytic domain
PF00930	Dipeptidyl peptidase IV (DPP IV) N-terminal region	PF05547	Immune inhibitor A peptidase M6
PF00949	Peptidase S7, Flavivirus NS3 serine protease	PF05572	Pregnancy-associated plasma protein-A
PF01019	Gamma-glutamyltranspeptidase	PF05576	PS-10 peptidase S37
PF01136	Peptidase family U32	PF05649	Peptidase family M13
PF01156	Inosine-uridine preferring nucleoside hydrolase	PF06114	IrrE N-terminal-like domain
PF01244	Membrane dipeptidase (Peptidase family M19)	PF06280	Fn3-like domain
PF01252	Signal peptidase (SPase) II	PF06821	Serine hydrolase
PF01321	Creatinase/Prolidase N-terminal domain	PF07486	Cell Wall Hydrolase
PF01343	Peptidase family S49	PF07504	Fungalysin/Thermolysin Propeptide Motif
PF01427	D-ala-D-ala dipeptidase	PF07687	Peptidase dimerisation domain
PF01431	Peptidase family M13	PF07722	Peptidase C26

PF01432	Peptidase family M3	PF07859	alpha/beta hydrolase fold
PF18294	Peptidase S41 N-terminal domain	PF07943	Penicillin-binding protein 5, C-terminal domain
PF01434	Peptidase family M41	PF07969	Amidohydrolase family
PF01435	Peptidase family M48	PF08439	Oligopeptidase F
PF01447	Thermolysin metallopeptidase, catalytic domain	PF08530	X-Pro dipeptidyl-peptidase C-terminal non-catalytic domain
PF01470	Pyroglutamyl peptidase	PF09375	Imelysin
PF01478	Type IV leader peptidase family	PF09752	Abhydrolase domain containing 18
PF01483	Proprotein convertase P-domain	PF10026	Predicted Zn-dependent protease (DUF2268)
PF01546	Peptidase family M20/M25/M40	PF10118	Predicted metal-dependent hydrolase
PF01551	Peptidase family M23	PF10502	Signal peptidase, peptidase S26
PF01750	Hydrogenase maturation protease	PF10503	Esterase PHB depolymerase
PF01955	Adenosylcobinamide amidohydrolase	PF12146	Serine aminopeptidase, S33
PF01979	Amidohydrolase family	PF12392	Collagenase
PF01981	Peptidyl-tRNA hydrolase PTH2	PF12695	Alpha/beta hydrolase family
PF02016	LD-carboxypeptidase N-terminal domain	PF12697	Alpha/beta hydrolase family
PF02031	Streptomyces extracellular neutral proteinase (M7) family	PF12706	Beta-lactamase superfamily domain
PF02073	Thermophilic metalloprotease (M29)	PF13242	HAD-hyrolase-like
PF02113	D-Ala-D-Ala carboxypeptidase 3 (S13) family	PF13354	Beta-lactamase enzyme family
PF02129	X-Pro dipeptidyl-peptidase (S15 family)	PF13365	Trypsin-like peptidase domain
PF02163	Peptidase family M50	PF13398	Peptidase M50B-like
PF02190	ATP-dependent protease La (LON) substrate-binding domain	PF13483	Beta-lactamase superfamily domain
PF02275	Linear amide C-N hydrolases, choloylglycine hydrolase family	PF13529	Peptidase_C39 like family
PF02517	CPBP intramembrane metalloprotease	PF13539	D-alanyl-D-alanine carboxypeptidase
PF02557	D-alanyl-D-alanine carboxypeptidase	PF13620	Carboxypeptidase regulatory-like domain
PF02617	ATP-dependent Clp protease adaptor protein ClpS	PF13715	CarboxypepD_reg-like domain
PF02633	Creatinine amidohydrolase	PF14684	Tricorn protease C1 domain
PF02789	Cytosol aminopeptidase family, N-terminal domain	PF16188	C-terminal region of peptidase_M24
PF02868	Thermolysin metallopeptidase, alpha-helical domain	PF16189	Creatinase/Prolidase N-terminal domain
PF03051	Peptidase C1-like family	PF17676	LD-carboxypeptidase C-terminal domain

Supplementary Table 2

The 91 prioritized significant correlations (ρ>0.3, pAdj<1E-5, one-sided *t*-test, adjusted by false-discovery rate, I>=10) of class III lanthipeptides, corresponding to Fig. 1C. Where ρ is Spearman's rank correlation coefficient, pAdj is false-discovery-rate adjusted P-value, I is the number of genomes that contain the correlation. Correlations were calculated for all genomes in each genus.

Genus	Precursor group	Protease group	ρ	pAdj	Protease name
Amycolatopsis	Pre_77	Prot_38	0.57	1.5E-07	class A beta-lactamase
		Prot_107	0.58	1.2E-07	carbon-nitrogen hydrolase family protein
		Prot_135	0.53	2.1E-06	alpha/beta hydrolase
		Prot_186	0.55	9.3E-07	S8 family peptidase
		Prot_188	0.52	6.7E-06	alpha/beta fold hydrolase
		Prot_251	0.53	2.3E-06	signal peptidase II
		Prot_404	0.61	1.1E-08	alpha/beta hydrolase
		Prot_616	0.61	8.2E-09	serine hydrolase
		Prot_678	0.63	1.5E-09	aminopeptidase P N-terminal domain-containing protein
		Prot_1177	0.70	2.1E-12	alpha-lytic protease prodomain-containing protein
		Prot_1416	0.59	5.5E-08	alpha/beta hydrolase
		Prot_2057	0.55	5.9E-07	alpha/beta fold hydrolase
		Prot_2172	0.60	1.7E-08	zinc carboxypeptidase
		Prot_2385	0.52	6.5E-06	hypothetical protein
		Prot_2470	0.56	3.7E-07	epoxide hydrolase
		Prot_2474	0.57	1.4E-07	trypsin-like serine protease
		Prot_2722	0.57	2.1E-07	serine hydrolase
		Prot_2758	0.54	1.7E-06	alpha/beta hydrolase
		Prot_3502	0.62	5.5E-09	serine protease
		Prot_3670	0.55	7.3E-07	M1 family metallopeptidase
		Prot_3737	0.65	2.3E-10	alpha/beta hydrolase
		Prot_3772	0.62	2.8E-09	C39 family peptidase
		Prot_3780	0.66	1.5E-10	snapalysin family zinc-dependent metalloprotease
		Prot_4468	0.54	1.4E-06	peptidase M28
		Prot_4613	0.72	2.4E-13	hypothetical protein AMED_6141
		Prot_4734	0.66	1.6E-10	amidohydrolase
		Prot_5282	0.56	4.7E-07	serine protease
		Prot_5512	0.61	8.1E-09	M23 family metallopeptidase
		Prot_6154	0.61	1.3E-08	serine hydrolase
		Prot_6257	0.65	2.0E-10	hypothetical protein
		Prot_6423	0.54	1.4E-06	prolyl oligopeptidase family serine peptidase
		Prot_6790	0.62	3.7E-09	alpha/beta hydrolase
		Prot_6852	0.54	1.1E-06	alpha/beta hydrolase
		Prot_7005	0.63	1.7E-09	M20/M25/M40 family metallo-hydrolase
		Prot_7969	0.53	2.4E-06	apolipoprotein acyltransferase
		Prot_8241	0.56	5.0E-07	peptidase M14
		Prot_8434	0.61	1.3E-08	S8 family serine peptidase
		Prot_8910	0.58	9.3E-08	CPBP family intramembrane metalloprotease
		Prot_9021	0.63	1.7E-09	alpha/beta hydrolase
		Prot_9217	0.56	4.7E-07	M36 family metallopeptidase
		Prot_9321	0.66	9.3E-11	hypothetical protein
Streptomyces	Pre_5	Prot_241	0.31	1.1E-29	metal-dependent hydrolase
		Prot_1365	0.33	1.6E-35	carbon-nitrogen hydrolase

	Pre_117	Prot_2222	0.36	6.3E-42	amidohydrolase family protein
		Prot_3737	0.34	4.4E-36	alpha/beta hydrolase
		Prot_8924	0.42	2.2E-57	alpha/beta hydrolase
		Prot_9754	0.42	7.4E-58	hypothetical protein
Lentzea	Pre_115	Prot_3654	1.00	0	M6 family metalloprotease domain-containing protein
Rhodococcus	Pre_228	Prot_162	0.40	1.8E-13	acetylornithine deacetylase
		Prot_471	0.35	2.5E-10	hypothetical protein
		Prot_526	0.34	7.6E-10	haloalkane dehalogenase
		Prot_616	0.30	1.9E-07	serine hydrolase
		Prot_777	0.36	6.2E-11	M20/M25/M40 family metallo-hydrolase
		Prot_1077	0.32	2.3E-08	alpha/beta fold hydrolase
		Prot_1090	0.38	2.4E-12	dipeptidase
		Prot_1187	0.33	3.5E-09	alpha/beta hydrolase
		Prot_1470	0.34	9.4E-10	alpha/beta hydrolase
		Prot_2030	0.39	1.6E-12	alpha/beta hydrolase
		Prot_2258	0.36	5.7E-11	alpha/beta hydrolase
		Prot_2365	0.31	6.2E-08	L,D-transpeptidase family protein
		Prot_2583	0.37	1.3E-11	serine hydrolase
		Prot_2588	0.38	7.7E-12	serine hydrolase
		Prot_2653	0.38	7.5E-12	amidohydrolase family protein
		Prot_2758	0.38	2.4E-12	alpha/beta hydrolase
		Prot_3414	0.39	7.3E-13	alpha/beta hydrolase
		Prot_3500	0.39	1.3E-12	dienelactone hydrolase family protein
		Prot_4387	0.40	1.7E-13	serine hydrolase
		Prot_4495	0.32	3.1E-08	signal peptidase I
		Prot_4734	0.31	1.1E-07	amidohydrolase
		Prot_4735	0.35	3.4E-10	CPBP family intramembrane metalloprotease
		Prot_5425	0.32	2.5E-08	dipeptidyl aminopeptidase
		Prot_5611	0.34	7.8E-10	esterase
		Prot_6285	0.35	3.4E-10	MFS transporter
		Prot_6362	0.40	1.0E-13	amidohydrolase family protein
		Prot_6528	0.34	1.4E-09	acetylxylan esterase
		Prot_6953	0.43	1.8E-15	serine hydrolase
		Prot_6968	0.37	2.9E-11	MBL fold metallo-hydrolase
		Prot_7970	0.35	5.2E-10	esterase family protein
		Prot_8326	0.38	2.4E-12	serine hydrolase, partial
		Prot_9463	0.36	1.6E-10	alpha/beta hydrolase
Alkalihalobacillus	Pre_134	Prot_12	0.62	4.1E-06	signal peptidase I
		Prot_1169	0.68	8.3E-08	DUF2268 domain-containing protein
		Prot_2308	0.60	9.6E-06	M20/M25/M40 family metallo-hydrolase
		Prot_6771	0.61	8.1E-06	alpha/beta hydrolase
		Prot_9033	0.77	2.8E-11	serine hydrolase
		Prot_9513	0.61	8.1E-06	CPBP family intramembrane metalloprotease
Lactobacillus	Pre_181	Prot_106	0.49	7.0E-31	Xaa-Pro peptidase family protein
		Prot_786	0.38	1.5E-17	hypothetical protein
		Prot_3778	0.41	1.9E-21	matrixin family metalloprotease
Paenibacillus	Pre_24	Prot_176	0.69	4.1E-64	insulinase family protein
		Prot_819	0.69	4.1E-64	insulinase family protein

Supplementary Table 3

Correlations between some known lanthipeptide precursors and their proteases. Correlations were calculated for all genomes in each genus. P-values were calculated by one-sided t-test and adjusted by false-discovery rate.

Genus	Peptide	Precursor	Precursor group	Protease	Protease group	Correlation
Staphylococcus	Epidermin	CAA44252.1	Pre_1	CAA44257.1	Prot_1211	ρ=0.97, pAdj=0, I=2955
	Gallidermin	ABC94902.1	Pre_1	ABC94907.1	Prot_1211	ρ=0.97, pAdj=0, I=2955
Bacillus	Thuricin	AHX39582.1	Pre_11	AHX39584.1	Prot_4437	ρ=0.90, pAdj=0, I=297
	Cerecidin	AHJ59543.1 AHJ59544.1 AHJ59545.1 AHJ59546.1 AHJ59547.1 AHJ59548.1 AHJ59549.1	Pre_12	AHJ59536.1	Prot_5982	ρ=0.72, pAdj=0, I=126
				AHJ59535.1	Prot_5818	ρ=0.85, pAdj=0, I=126
Enterococcus	Cytolysin	AAA62648.1	Pre_10	AAA62651.1	Prot_5642	ρ=0.90, pAdj=0, I=239
				AAA62652.1	Prot_5225	ρ=0.92, pAdj=0, I=250
		AAA62649.1	Pre_13	AAA62651.1	Prot_5642	ρ=0.90, pAdj=0, I=239
				AAA62652.1	Prot_5225	ρ=0.92, pAdj=0, I=250

Supplementary Table 4
Bacterial strains and plasmids in this study.

Strain	Strain/genome source
Paenibacillus thiaminolyticus NRRL B4156	Purchased from NRRL (ARS culture colletion, USA)<br>NCBI RefSeq accession: NZ_CP041405.1<br>[https://www.ncbi.nlm.nih.gov/nuccore/NZ_CP041405.1]
Bacillus nakamurai NRRL B41092	Purchased from NRRL (ARS culture colletion, USA)<br>NCBI RefSeq accession: NZ_LSBA00000000.1<br>[https://www.ncbi.nlm.nih.gov/nuccore/NZ_LSBA00000000.1]
Paenibacillus taiwanensis DSM18679	Purchased from DSMZ (German Collection of Microorganisms and Cell Cultures, Germany)<br>NCBI RefSeq accession: NZ_KE384306.1<br>[https://www.ncbi.nlm.nih.gov/nuccore/NZ_KE384306.1]
Catenulispora acidiphila DSM44928	Purchased from DSMZ (German Collection of Microorganisms and Cell Cultures, Germany)<br>NCBI RefSeq accession: NC_013131.1<br>[https://www.ncbi.nlm.nih.gov/nuccore/NC_013131.1]
Paenibacillus polymyxa ATCC842	Purchased from BGSC (Bacillus Genetic Stock Center, USA)<br>NCBI RefSeq accession: NZ_GL905390.1<br>[https://www.ncbi.nlm.nih.gov/nuccore/NZ_GL905390.1]
B. subtilis 168	Purchased from BGSC (Bacillus Genetic Stock Center, USA)
E. coli DH10B	Purchased from NEB (New England Biolabs, Inc., USA)
E. coli BL21 (DE3)	Purchased from NEB (New England Biolabs, Inc., USA)
B. subtilis 168Δ*ymfFH*	This study
B. subtilis 168_pDR111	This study
B. subtilis 168_*ptt*	This study
B. subtilis 168_*ptt*Δ*pttP1*/*pttP2*+*ptt-gP1*/*ptt-gP2*	This study
B. subtilis 168_*bcn+bnc-gP1/bcn-gP2*	This study
B. subtilis 168Δ*ymfFH_ptt*	This study
B. subtilis 168Δ*ymfFH_pttA1+pttKC+pttP1*	This study
B. subtilis 168Δ*ymfFH_pttA1+pttKC+pttP2*	This study
B. subtilis 168Δ*ymfFH_pttA1+pttKC+pttP1/pttP2*	This study
B. subtilis 168Δ*ymfFH_pttA1+pttP1/pttP2*	This study
**Plasmid**	**Source**
pDR111	Purchased from BGSC (Bacillus Genetic Stock Center, USA)
pHis8	Gift, Supplementary Reference 1
pET-28a(+)	Purchased from NEB (New England Biolabs, Inc., USA)
pJOE8999	Gift, Supplementary Reference 2
pJOE8999.1	This study
pJOE8999.2	This study
pDR111-*ptt*	This study
pDR111-*ptt*Δ*pttP1*/*pttP2*+*ptt-gP1*/*ptt-gP2*	This study
pDR111-*bcn+bcn-gP1/bcn-gP2*	This study
pDR111-*pttA1+pttKC*	This study
pDR111-*pttA1+pttKC+pttP1*	This study

pDR111-*pttA1+pttKC+pttP2*	This study
pDR111-*pttA1+pttKC+pttP1/pttP2*	This study
pDR111-*pttA1+pttP1/pttP2*	This study
pET28-*SUMO-pttA1*	This study
pET28-*SUMO-pttA2*	This study
pET28-*SUMO-pttA3*	This study
pET28-*SUMO-pttA5*	This study
pET28-*SUMO-pttA7*	This study
pHis8-*pttKC*	This study
pHis8-*pttP1*	This study
pHis8-*pttP2*	This study
pHis8-*ptt-gP1*	This study
pHis8-*ptt-gP2*	This study
pHis8-*ymfF*	This study
pHis8-*ymfH*	This study
pHis8-*homoAplP*	This study
pET28-*SUMO-bcnA1*	This study
pET28-*SUMO-bcnA2*	This study
pHis8-*bcnKC*	This study
pHis8-*bcn-gP1*	This study
pHis8-*bcn*-g*P2*	This study
pET28-*SUMO-pbtA*	This study
pHis8-*pbtKC*	This study
pHis8-*pbt-gP1*	This study
pHis8-*pbt-gP2*	This study
pHis8-*aciKC*	This study
pHis8-*aciP*	This study
pHis8-*aci*-gP1	This study
pHis8-*aci*-gP2	This study
pET28-SUMO-*pllA*	This study
pHis8-*pllB*	This study
pHis8-*pllC1*	This study
pHis8-*pllC2*	This study
pHis8-atcc842-*pro686*	This study
pHis8-p*ttP1*-R298A	This study
pHis8-*pttP1*-Y305A	This study
pHis8-*pttP1-ΔRY*	This study
pHis8-*pttP2*-H67A	This study
pHis8-*pttP2*-E70A	This study
pHis8-*pttP2*-H71A	This study
pHis8-*pttP1-ΔZn*	This study

Supplementary Table 5
Primers used in this study

Primers	Sequence (5'→3')
sgRNA-ymfFH_F	tacgagccagtcttgcttactacg
sgRNA-ymfFH_R	aaaccgtagtaagcaagactggct
pJOE8999.2_F	accgtgattagagaattgagtaaaatgtacctacgagccagtcttgcttactacgc
pJOE8999.2_R	agccttattttaacttgctatttctagctctaaaaccgtagtaagcaagactggct
ptt_F	attgtgagcggataacaattaagcttagtcgactcttttacgctttctttaagtatgga
ptt_R	tcgtttccaccgaattagcttgcatgctcacacattcccactaagaacca
pttΔpttP1/pttP2_R	ttagcagcacttataaacact
pttΔpttP1/pttP2+ptt-gP1/ptt-gP2_F	tcggtattaagtgtttataagtgctgctaatccatgacgaagaagcaatggt
pttΔpttP1/pttP2+ptt-gP1/ptt-gP2_ R	tctagtaatagagtactgttctctctagttcaatccggcttctcgacgatcga
pttΔpttP1/pttP2_F	actagagagaacagtactctattact
bcn_F	attgtgagcggataacaattaagcttagtcgacagtgagacggtatttattggtttct
bcn_R	tcatagcgccttctacttcttctctgccctagaagtcct
bcn-gP1/bcn-gP2_F	tctagggcagagaagaagtagaaggcgctatgatca
bcn-gP1/bcn-gP2_R	tcgtttccaccgaattagcttgcatgctgctgtcggattcatcactca
pttA1+pttKC_F	tgtattatataaaaaatttagcagttatatccccctcattggcaatta
pttA1+pttKC_R	agggggatataactgctaaattttttatataatacaaaactaga
pttA1+pttKC+pttP1_F	tatatcagaaaagtgttgatttactcctttctatttaaaaagtagattgt
pttA1+pttKC+pttP1_R	ttttaaatagaaaggagtaaatcaacacttttctgatatatccaga
pttA1+pttKC+pttP2_F	tttattctgtttatctgatcaactatctcctatatccccctcatt
pttA1+pttKC+pttP2_R	atataggagatagttgatcagataaacagaataaatagggt
pttA1+pttKC+pttP1/pttP2_F	tgtgagcggataacaattaagcttagtcgactcttttacgctttctttaagtatgga
pttA1+pttKC+pttP1/pttP2_ R	tcgtttccaccgaattagcttgcatgctcaccgttgttcactagtga
pttA1+pttP1/pttP2_F	tgtgagcggataacaattaagcttagtcgactcttttacgctttctttaagtatgga
pttA1+pttP1/pttP2_R	cgtttccaccgaattagcttgcatgcttaatccccgtttacaattaatgt
SUMO-pttA1_F	atccgaaaacctgtattttcagggcgtgaaaaaaatgaatgccgt
SUMO-pttA1_R	tgtcgacggagctcgaattcttagcagcacttataaacact
SUMO-pttA2_F	atccgaaaacctgtattttcagggcatgaacgccgtattggaattaca
SUMO-pttA2_R	tgtcgacggagctcgaattcctaatttttgtaacactcccaagca
SUMO-pttA3_F	atccgaaaacctgtattttcagggcatgaacgccgtattggaattaca
SUMO-pttA3_R	tgtcgacggagctcgaattctcattggcggcacaaaaaattact
SUMO-pttA5_F	atccgaaaacctgtattttcagggcgtgaaaaaaatgaacgcagt
SUMO-pttA5_R	tgtcgacggagctcgaattcttaattgcacttataaacacttaaggt
SUMO-pttA7_F	atccgaaaacctgtattttcagggcgtgaagaaaatgaacgcagt
SUMO-pttA7_R	tgtcgacggagctcgaattcttatgaattgcactgataaacact
His8-pttKC_F	tggtctggttccgcgtggttccatggaaggaaatatgctttatca
His8-pttKC_R	aagcttgtcgacggagctcgaattctcaccgttgttcactagtga

His8-pttP1_F	tggtctggttccgcgtggttccatggatgaaatagctattcgt
His8-pttP1_R	aagcttgtcgacggagctcgaattcttactcctttctatttaaaaagtaga
His8-pttP2_F	tggtctggttccgcgtggttccatggagtcaatggtctataaaaga
His8-pttP2_R	aagcttgtcgacggagctcgaattcttaatccccgtttacaattaatgt
His8-ptt-gP1_F	tggtctggttccgcgtggttccatggagaagcaaccgacgt
His8-ptt-gP1_R	aagcttgtcgacggagctcgaattcttatacctcctcccggttgc
His8-ptt-gP2_F	tggtctggttccgcgtggttccatggaacaacgccgctattccca
His8-ptt-gP2_R	aagcttgtcgacggagctcgaattctcaatccggcttctcgacgatcga
His8-ymfF_F	tggtctggttccgcgtggttccatgtcatatgttaatgaactcaaatca
His8-ymfF_R	aagcttgtcgacggagctcgaattcattggtttgatcaagatgca
His8-ymfH_F	tggtctggttccgcgtggttccatgatcaaaccaatcgaatatgaaca
His8-ymfH_R	aagcttgtcgacggagctcgaattcttatgatttaggaacaaccttgca
His8-homoAplP_F	tggtctggttccgcgtggttccatgaaaatgacaggacgccaactg
His8-homoAplP_R	aagcttgtcgacggagctcgaattcttattggaatacttgggaaatcggattttca
SUMO-bcnA1_F	atccgaaaacctgtattttcagggcatgaacgccgtattggaa
SUMO-bcnA1_R	tgtcgacggagctcgaattcttagcaatgattacttactgtcga
His8-bcnKC_F	tggtctggttccgcgtggttccatggaaggtaatatgctttatca
His8-bcnKC_R	aagcttgtcgacggagctcgaattcttatttaattagttcactagttactgataga
His8-bcn-gP1_F	tggtctggttccgcgtggttccatgacttacgtaaatgaaatgaagt
His8-bcn-gP1_R	aagcttgtcgacggagctcgaattctcggtttagtcaagatgct
His8-bcn-gP2_F	tggtctggttccgcgtggttccatgactaaaccgatagaattcga
His8-bcn-gP2_R	aagcttgtcgacggagctcgaattcttatgattttggcactactttgca
SUMO-pbtA_F	atccgaaaacctgtattttcagggcatgggtagaattctcaatcttca
SUMO-pbtA_R	tgtcgacggagctcgaattcaggacagtgtgaatcacact
His8-pbtKC_F	tggtctggttccgcgtggttccatgaagccggaatttctcaggt
His8-pbtKC_R	aagcttgtcgacggagctcgaattcttacaccgtaacgcttgcgt
His8-pbt-gP1_F	tggtctggttccgcgtggttccatgacaaatcggacgcctgct
His8-pbt-gP1_R	aagcttgtcgacggagctcgaattcctatacctcctctcgattgcgca
His8-pbt-gP2_F	tggtctggttccgcgtggttccatggaacaacgacagtttaaaca
His8-pbt-gP1_R	aagcttgtcgacggagctcgaattcttaaggctgctctacgatcga
His8-aciKC_F	tggtctggttccgcgtggttccatggacgaccgctacgagg
His8-aciKC_R	aagcttgtcgacggagctcgaattctcaccttcctgtttttggctgc
His8-aciP_F	tggtctggttccgcgtggttccgtgcccggtactaatctgacccgc
His8-aciP_R	aagcttgtcgacggagctcgaattctcaggccgcggagcggtc
His8-aci-gP1_F	tggtctggttccgcgtggttccatgagcaataccctgatttccga
His8-aci-gP1_R	aagcttgtcgacggagctcgaattctcaagcctggccctcgtc
His8-aci-gP2_F	tggtctggttccgcgtggttccgtggtcagccccgacccca
His8-aci-gP2_R	aagcttgtcgacggagctcgaattctcatgctccggcctcctc

SUMO-pllA_F	atccgaaaacctgtattttcagggcatgaaaaatcaatttgatctgga
SUMO-pllA_R	tgtcgacggagctcgaattcttaacacgttttgcagaaaga
His8-pllB_F	tggtctggttccgcgtggttccatgaagagacaactgcattaca
His8-pllB_R	aagcttgtcgacggagctcgaattctcattggtttgtcctttagact
His8-pllC1_F	tggtctggttccgcgtggttccatggttggagatttgcagga
His8-pllC1_R	aagcttgtcgacggagctcgaattcttacgaaattagaaacaattcactcca
His8-pllC2_F	tggtctggttccgcgtggttccatgaaaacggaccattttgt
His8-pllC2_R	aagcttgtcgacggagctcgaattcctaggatagcatgagcatgt
His8-atcc842-pro686_F	tggtctggttccgcgtggttccatggaacgtaaagtgcatatca
His8-atcc842-pro686_R	aagcttgtcgacggagctcgaattcttatttaaccttcaacgatgctgt
pttP1-R298A_F	attcattaatgtggcagaaaagaaaagtct
pttP1-R298A_R	agacttttcttttctgccacattaatgaat
pttP1-Y305A_F	agaaaagtcttgctgcatttgtattttccca
pttP1-Y305A_R	tgggaaaatacaaatgcagcaagacttttct
pttP2-H67A_F	agatggaatagcggcgtttttagaacataa
pttP2-H67A_R	ttatgttctaaaaacgccgctattccatct
pttP2-E70A_F	agcgcatttttttagcgcataaaatgtttga
pttP2-E70A_R	tcaaacattttatgcgctaaaaaaatgcgct
pttP2-H71A_F	catttttagaagcgaaaatgtttgaaagt
pttP2-H71A_R	actttcaaacattttcgcttctaaaaaatg

Supplementary Table 6
Retention times (t_R, min) of FDAA derivatives of amino acid (AA) standards and of the hydrolysates of paenithopeptin A

AA	MW (AA-FDAA)	AA standards		hydrolysate of **Paenithopeptin A**		
		t_R (L-AA-L-FDAA) or t_R (D-AA-D-FDAA) (min)	t_R (L-AA-D-FDAA) or t_R (D-AA-L-FDAA) (min)	t_R (AA-L-FDAA) (min)	t_R (AA-D-FDAA) (min)	Absolute configuration
Ala	341	13.9	15.6	13.8	15.6	**L**
Ile	383	19.0	21.5	19.0	21.5	**L**
Val	369	17.1	19.5	17.0	19.4	**L**
Leu	383	19.5	21.8	19.4	21.8	**L**
Tyr	433	22.9	24.9	23.0	25.0	**L**
Lys	398	20.1	21.1	20.2	21.1	**L**

Samples were analyzed on HPLC-DAD-ESIMS (Kinetex C18 HPLC column, 4.6 × 100 mm, 2.4 μm, 100 Å, 1.0 mL/min gradient elution from 95% to 38% H_2O/MeCN over 30 min with constant 0.1% formic acid; positive and negative ionization modes; UV 340 nm).

Supplementary Note

Structure elucidation of bacinapeptins A and B

The *n*-butanol extract of *B. nakamurai* NRRL B-41092 broth was analyzed by the high-performance liquid chromatography-electrospray ionization mass spectrometry (HPLC-ESI-MS). A doubly charged molecular ion at *m/z* 1113.0 $[M + 2H]^{2+}$ was detected, indicative of a molecular formula $C_{106}H_{141}N_{27}O_{25}S$ (Supplementary Fig. 6a). Subsequent collision-induced dissociation tandem mass spectrometry (CID-MS/MS) analysis suggested that the mature peptide, namely bacinapeptin A, contains 23 amino acid residues including 11 dehydrated amino acids (e.g. Dhb, Dha) as well as a *N*,*N*-dimethylated alanine (Supplementary Fig. 6b). Based on the diagnostic fragment ions (b and y ions) generated by MS/MS, partial sequences of bacinapeptin A were established to be Dhb-Dhb-Dhb-Trp-Dhb-Val-Dhb-Dhb-Dhb and Ala-Phe, which are consistent with the predicted core peptide sequences prior to post-translational modifications, i.e. Thr-Thr-Thr-Trp-Thr-Val-Thr-Thr-Thr (TTTWTVTTT) and Ala-Phe (AF), respectively. Moreover, the lack of diagnostic fragments ions for the remaining sequence Ser-Thr-Val-Ser-Asn-His-Cys (STVSNHC) at the C-terminus suggested the formation of lanthionine (LAN) or labionin (LAB) ring, which stabilizes the structure. Considering the nature of labionin formation, we proposed that a C-terminal bicyclic labionin ring was generated through Michael addition cyclization among Dha17, Dha20, and Cys23 in bacinapeptin A. Except for bacinapeptin A, another minor peak at *m/z* 1102.6 $[M + 2H]^{2+}$ was detected in the butanol extract of *B. nakamurai* NRRL B-41092(Supplementary Fig. 7a). It showed a similar MS/MS fragmentation pattern to bacinapeptin A, which we named bacinapeptin B. Detailed interpretation of MS/MS spectrum of bacinapeptin B returned a partial peptide sequence Dhb-Dhb-Dhb-Dha-Gly-Phe-Ile, corresponding to a part of predicted core peptide sequence Thr-Thr-Thr-Ser-Gly-Phe-Ile (TTTSGFI) prior to post-translational modifications, which is different from that for bacinapeptin A (TTTSAFT). Moreover, the y_7 ion in bacinapeptin B is 37 Da less than that in bacinapeptin A, suggesting a Dhb and a His in the labionin ring may be replaced by Dha and Asn in bacinapeptin B (Supplementary Fig. 7b). This hypothesis was further validated by the predicted core peptide sequence of bacinapeptin B, in which Thr18 was replaced by Ser18, and His22 was replaced by Asn22. The amino acid residues involved in labionin formation and *N*,*N*-dimethylation remain identical.

Paenithopeptin A structure elucidation

High-resolution mass spectrometry (HRMS) analysis of paenithopeptin A returned a doubly charged (*m/z* 948.4825) and a triply charged (*m/z* 632.6581) molecular ions, indicative of a molecular formula $C_{87}H_{138}N_{20}O_{21}S_3$ (Δppm -0.42) (Supplementary Fig. 9). Collision-induced dissociation tandem mass spectrometry (CID-MS/MS)-based peptide sequencing of paenithopeptin A revealed diagnostic fragment ions (b and y ions), matching to the molecular weight of single amino acid residue or their combinations, such as dehydrobutyrine (Dhb), isoleucine (Ile), and the dipeptide alanine-glycine (Ala-Gly) (Supplementary Fig. 10). Careful analysis of the fragment ions established the partial sequence of paenithopeptin A to be Ala-Gly-Ile-Dhb-Dhb-Ile-Ile-Dhb-Ile, which is consistent with the predicted core peptide sequence, Ala-Gly-Ile-Thr-Thr-Ile-Ile-Thr-Ile (AGITTIITI), prior to post-translational modification (e.g. dehydration of Thr to Dhb). However, the remaining core peptide sequence Cys-Ser-Val-Leu-Ser-Val-Tyr-Lys-Cys-Cys (CSVLSVYKCC) at the C-terminus cannot be deduced solely from MS/MS fragmentation analysis due to the lack of corresponding fragment ions, suggesting the formation of lanthionine (LAN), labionin (LAB), or disulfide ring that stabilizes the peptide structure. Co-incubation of paenithopeptin A with the redox reagent dithiothreitol (DTT) led to the yield of a reduced paenithopeptin A' with the mass of 2 Da larger than that of paenithopeptin A, highly suggesting the reduction of a disulfide bond to form two sulfhydryl groups (Supplementary Fig. 11). Subsequent MS/MS analysis of paenithopeptin A' revealed a similar fragmentation pattern to that of paenithopeptin A, but with significant differences being that (i) the molecular weights of all the y ions are 2 Da larger than those for paenithopeptin A; and (ii) a doubly charged b_{18} ion (*m/z* 889.0) deficient in the cysteine residue at the far end of C-terminus was observed (Supplementary Fig. 12). These observations indicated that a disulfide bridge is highly likely formed between Cys19 and Cys10. Moreover, the mutation of Cys19 to Ala19 abolished the formation of disulfide bridge as evidenced form the LC-MS and MS/MS analysis (Supplementary Fig. 13), reinforcing that Cys19 is involved in the formation of disulfide bond in paenithopeptin A. Analogous to labyrinthopeptin, we proposed that a labionin ring, characteristic of class III lanthipeptides, is formed among Cys18, Dha15 and Dha11 to generate a bicyclic ring system at the C-terminus.

To prove this hypothesis, the mutant C18A was established and showed the abolishment of peptide production (Supplementary Fig. 13), probably due to the instability of the peptide prior to labionin formation and thus supporting the labionin formation. 1D and 2D NMR analysis (DMSO-d_6) of paenithopeptin A (Supplementary Figs. 14-20) revealed two pairs of olefinic signals (δ_H 6.65, δ_C 115.3; δ_H 6.91, δ_C 130.5) representing the *para*-substituted phenolic ring in tyrosine residue, and three olefinic signals (δ_H 6.44, δ_C 129.9; δ_H 6.44, δ_C 129.9; δ_H 6.28, δ_C 127.6) for three Dhb residues. However, signals for Dha were not observed in the NMR spectra of paenithopeptin A, indicating all Dha residues are substituted and highly likely form labionin ring with Cys18. Marfey's analysis demonstrated all the proteinogenic amino acids in paenithopeptin A possess L-configuration (Supplementary Table 4).

Networking analysis via Spearman's rank-order correlation

We first attempted to adapt a co-occurrence networking analysis[2] by the presence or absence of protease group and precursor group in genomes, using Fisher's exact test. However, if we only considered the presence or absence of protease and precursor, the information of their counts in one genome was lost. We found that if almost all genomes have the protease "presence" as they harbored multiple copies of proteases, then Fisher's exact test could not find the association between precursors and this protease. Therefore, we used Spearman's rank-order correlation which takes the number of proteases and precursors into account. We rationalized that for the bacterial strains that harbor multiple copies of proteases, the additional proteases might be used as the precursor-specific protease. Thus, the number of proteases may increase as precursors present or increase, and this association could be captured by Spearman's rank correlation coefficient.

Bacterial strains with close phylogenetic distance (e.g. different strains in the same species, different species in the same genus, etc.) have very similar genomic contents, thus these strains usually carry the same composition of protease groups and precursor groups. If correlation analysis were done in all bacterial genomes, phylogenetic relatedness, instead of precursor-protease specificity, would become the major factor, which would consequently generate uninformative correlations. Therefore, we tried to perform our analysis on each phylogenetic level from species to phylum (i.e. only genomes within the same phylogenetic level were chosen to perform the analysis at one time, genomes outside this level were ignored). In higher phylogenetic levels such as Phylum or Class, uninformative correlations due to phylogenetic relatedness instead of precursor-protease specificity were still dominant, yet in lower phylogenetic levels such as Species, sample size becomes very small for some species. We finally chose genus as a compromise of both phylogenetic relatedness and sample size. At the genus level, genomes in the same genus can be considered relatively independent of each other, thus reducing uninformative correlations.

Supplementary References:

1. Teufel, R., Miyanaga, A., Michaudel, Q. *et al.* Flavin-mediated dual oxidation controls an enzymatic Favorskii-type rearrangement. *Nature* **503**, 552–556 (2013).
2. Li, YX., Zhong, Z., Hou, P. *et al.* Resistance to nonribosomal peptide antibiotics mediated by D-stereospecific peptidases. *Nat Chem Biol* **14,** 381–387 (2018).
