# Supplementary material for: Correlational networking guides the discovery of unclustered lanthipeptide protease-encoding genes
Source: Nat Commun. 2022 Mar 28;13:1647. doi: 10.1038/s41467-022-29325-1 (PMC8960859; doi:10.1038/s41467-022-29325-1)
Supplement: Supplementary file 3 — Description of Additional Supplementary Files [file 41467_2022_29325_MOESM3_ESM.pdf]

Description of Additional Supplementary files

File name: Supplementary Data 1

Description: Excel file containing all source data of correlational networks and co-expression analyses, as well as genome/transcriptome accession numbers.
Sheet1: 81,323 genus level correlations with $\rho>0$, corresponding to Fig. 1B
Sheet2: 5,209 genus level correlations with $\rho>0.3$, pAdj0.5, pAdj=10, corresponding to Fig. S3
Sheet4: genus level correlations between FlaA-like precursors and FlaP-like proteases, corresponding to Fig. S5
Sheet5: co-expression analysis of Pre_5 and its genomic correlated proteases, corresponding to Fig. 1C
Sheet6: co-expression analysis of Pre_49 and its genomic correlated proteases, corresponding to Fig. 2C
Sheet7: 10,263 protease group representative protein name and Pfam (with score)
Sheet8: 29,489 lanthipeptide precursor and their group
Sheet9: 80 SRA run accessions used in this study, and their run information
Sheet10: 161,954 RefSeq genome accessions used in this study, and their taxonomy information
